# Genetic Diagnosis and Discovery Enabled by Large Language Models

**DOI:** 10.1002/advs.202518656

**Published:** 2026-02-08

**Authors:** Tao Tu, Khaled Saab, Weida Liu, Zhouqing Fang, Zhuanfen Cheng, Svetolik Spasic, Maja Djurisic, Hiroaki Mohri, Wenlong Ren, Anil Palepu, Juraj Gottweis, Alan Karthikesalingam, Kavita Kulkarni, Annalisa Pawlosky, Devon Bonner, Elijah Kravets, Shruti Marwaha, Hector R. Mendez, Matthew T. Wheeler, Jonathan A. Bernstein, Undiagnosed Diseases Network, Cheng‐Yu Tsai, Chen‐Chi Wu, Konstantina M. Stankovic, Vivek Natarajan, Gary Peltz

**Affiliations:** ^1^ Google DeepMind Mountain View California USA; ^2^ Department of Anesthesiology Pain and Perioperative Medicine Stanford University School of Medicine Stanford California USA; ^3^ Department of Otolaryngology – Head and Neck Surgery Stanford University School of Medicine Stanford California USA; ^4^ Google Research Mountain View California USA; ^5^ Stanford Center For Undiagnosed Diseases Stanford California USA; ^6^ Department of Medicine Stanford University School of Medicine Stanford California USA; ^7^ Department of Pediatrics Stanford University School of Medicine Stanford California USA; ^8^ Department of Otolaryngology National Taiwan University College of Medicine Taipei Taiwan; ^9^ Department of Otolaryngology National Taiwan University Hospital Taipei Taiwan; ^10^ Department of Medical Research National Taiwan University Hospital Hsin‐Chu Branch Hsinchu Taiwan; ^11^ Department of Otolaryngology National Taiwan University Hospital Hsin‐Chu Branch Hsinchu Taiwan

**Keywords:** artificial intelligence, genetic discovery, large language model

## Abstract

Artificial intelligence (AI) has been used in many areas of medicine, and large language models (LLMs) have shown potential utility for various clinical applications. However, to determine if LLMs can accelerate the pace of genetic diagnosis and discovery, we examined whether recently developed LLMs (Med‐PaLM 2 and Gemini) could assist in solving four types of genetic problems with sequentially increasing complexity. First, in response to free‐text input, Med‐PaLM 2 correctly identified murine genes with experimentally verified causative genetic factors for six previously studied murine models of biomedical traits. Second, Med‐PaLM 2 identified a novel causative murine genetic factor for spontaneous hearing loss that was validated using knock‐in mice. Third, we developed a retrieval and grounding pipeline that enabled Gemini 2.5 Pro to analyze large lists of genes, which contained genetic variants that were identified in the genomic sequences of 20 human subjects with hearing loss, and demonstrated that it can assist in identifying causative genetic factors for hearing loss. Fourth, we modified the genetic analysis pipeline to enable Gemini 2.5 Pro without any task‐specific fine‐tuning to identify causative genetic factors for six subjects with rare genetic diseases, which required 14 to 34 different terms to describe their multi‐faceted symptom complexes. These results demonstrate that an AI pipeline can facilitate genetic diagnosis and discovery in mice and humans.

## Graphical abstract

We demonstrate that large language models (LLMs) can facilitate genetic diagnosis and discovery. LLMs were used to solve four types of genetic problems of sequentially increased complexity. An LLM‐based pipeline could analyze genetic variants in the genomic sequences of human hearing loss or rare genetic disease patients and assist in identifying causative genetic factors.

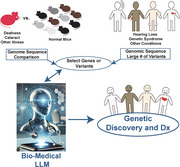

**Abbreviations**
ABR: Auditory brainstem response
AD: autosomal dominant
CoT: chain‐of‐thought
DPOAE: distortion product otoacoustic emission
LLM: large language model
MGD: monogenetic disease
SDS‐PAGE: SDS polyacrylamide gel electrophoresis
SGN: spinal ganglion neuron
SPL: sound pressure level
T3: triiodothyronine
VUS: variants of unknown significance

## 1 Introduction

A major challenge in biomedical science is to identify genetic factors in a population that influence the properties (i.e., phenotypes or traits) of an individual, especially those for disease susceptibility. Many genetic discoveries have been made using genome‐wide association study (GWAS) methods, which compare the pattern of allelic differences in mouse or human populations with variation in phenotypic responses. Irrespective of whether the subjects are mouse or human, a major barrier for genetic discovery is that GWAS results will identify a true causative genetic variant along with multiple other false positive associations because allelic patterns within commonly inherited genomic regions can randomly correlate with any phenotypic response pattern in a population. To address this issue, we recently developed an AI‐based computational pipeline for mouse genetic discovery that can sift through a set of candidate genes emerging from a GWAS and identify those most likely to be causal based upon assessment of candidate gene‐phenotype relationships in the published literature [1]. This pipeline analyzed publicly available datasets of biomedical responses measured in panels of inbred strains and identified novel causative genetic factors for various disease models. However, this pipeline could not respond to a free‐text query, and it could only analyze a specific dataset contained within a public database after its numeric label was entered.

The utility of a genetic discovery AI would be greatly expanded if it could answer free‐text queries and could utilize more complex analytic capabilities than were possible with our existing AI. Consistent with this possibility, large language models (LLMs) have recently been developed, which are general‐purpose AI systems that are commonly implemented with transformer‐based neural network architectures [2]. They are typically pretrained on internet‐scale text corpora [3] and have demonstrated a wide range of language understanding and processing capabilities, including information extraction, reasoning, summarizing data, and can use web and biomedical literature searches for information retrieval. While general‐purpose LLMs have demonstrated their capabilities across a wide range of applications, the uniqueness of the scientific and biomedical domain requires that they be further specialized and adapted. LLM task performance can be improved through either fine‐tuning, which uses high‐quality domain‐specific texts, or through integration into agentic systems that enable retrieval from external knowledge sources, tool‐use to perform specific tasks, and grounding in verified information sources to ensure factuality and supportable reasoning. For example, Med‐PaLM 2 is a medically aligned LLM that was fine‐tuned using high‐quality biomedical text corpora and was then aligned using clinician feedback. It demonstrated significantly improved performance when answering medical questions relative to general‐purpose LLMs, and its answers matched or exceeded those of physicians [4, 5]. A next‐generation multimodal Gemini model was developed with the ability to process images, audio, video, and text, and it has reasoning capabilities that could extend over millions of tokens. This Gemini model has achieved state‐of‐the‐art performance among frontier general‐purpose LLMs for many challenging tasks that include math, coding, and academic benchmarks [6]. Despite these advances and the large volume of biomedical and scientific knowledge encoded within LLMs, it remains to be determined if LLMs can generate novel hypotheses that facilitate genetic discovery.

**FIGURE 1:**
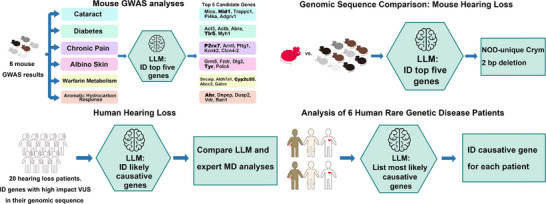
A schematic diagram of the 4 types of genetic problems analyzed by LLMs (Med‐PaLM 2 or Gemini 2.5 Pro) in this paper. First, we investigated whether Med‐PaLM 2 could analyze lists of genes generated from previously performed GWAS, which examined six murine models of biomedical traits, and correctly identify the murine genes with experimentally verified causative genetic factors. Second, we determined whether Med‐PaLM 2 could analyze genes with high‐impact alleles, which were identified by comparison of the genomic sequences of inbred mouse strains with and without hearing loss, and identify a genetic factor contributing to spontaneous hearing loss. Third, we developed a pipeline that enabled Gemini 2.5 Pro to analyze large lists of genes, which contained genetic variants of unknown significance (VUS), that were identified in the genomic sequences of 20 human subjects with hearing loss, and determined whether it could assist in identifying causative genetic factors for hearing loss. Fourth, we modified the genetic analysis pipeline and determined if Gemini 2.5 Pro could identify causative genetic factors in the genomic sequences of six human subjects with rare genetic diseases.

Here, we investigate whether these LLMs could facilitate genetic discovery and diagnosis by tasking them with four types of genetic problems with sequentially increasing complexity. Analyses were first performed using data obtained from mouse models, and then human genomic sequences obtained from patients were examined. Med‐PaLM 2 accurately interpreted free‐text queries about murine candidate genes and identified a novel causative murine genetic factor for hearing loss. To further augment the power of LLMs, we designed a novel tool‐use system based on Gemini 2.5 Pro that generates hypotheses based on analysis of the biomedical literature, and it then ranks the hypotheses that it generated. Besides streamlining the process of genetic analysis, the explanations provided by it increase confidence in its output. This system was used to identify potential genetic factors for hearing loss in 20 human patients, which is a common clinical condition. Lastly, a modified AI pipeline was used with this Gemini system to analyze genomic sequence data obtained from patients with rare genetic diseases that present with multiple and complex clinical symptoms (Figure 1).

## 2 Results

### 2.1 Assessing Murine Candidate Gene Sets

To test whether Med‐PaLM 2 could facilitate genetic discovery in response to free‐text input, it was asked to analyze sets of candidate genes, which were identified by analysis of mouse GWAS data for six previously studied biomedical traits [1, 7, 8, 9]. Med‐PaLM 2 correctly identified the gene with the experimentally verified causative factor (Table S1, Data File S1). These results indicate that Med‐PaLM 2 can analyze a list of genes and identify those most likely to be responsible for a studied trait by assessing gene‐phenotype relationships.

### 2.2 A Digenic Model for Hearing Loss

**FIGURE 2:**
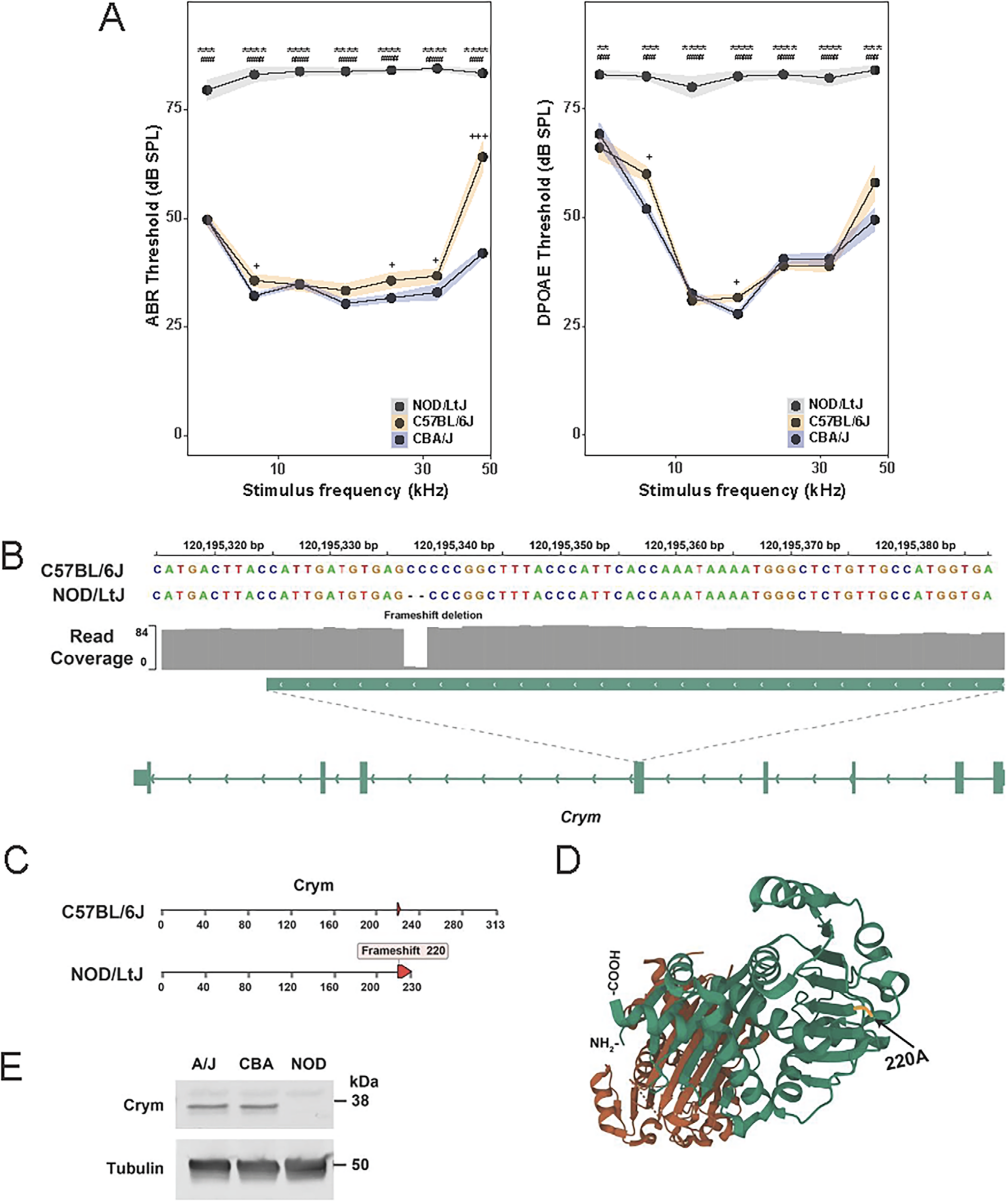
NOD/LtJ mice have a severe hearing loss due to a *Crym* mutation. (A) Auditory brainstem responses (ABR; left panel) and distortion product otoacoustic emissions (DPOAE; right panel) were measured in 7‐week‐old CBA/J (*n* = 10, blue), C57BL/6J (*n* = 11, orange), and NOD/LtJ (*n* = 7, gray) mice. Black symbols are threshold means for each frequency tested, and the standard error of the mean is demarcated by the colored ribbon. The ABR threshold levels for NOD/LtJ mice demonstrate that they have a profound hearing loss compared to that of CBA/J and C57BL/6J mice of the same age. The DPOAE thresholds in NOD/LtJ mice are substantially elevated across all frequencies tested in comparison to those of CBA/J and C57BL/6 mice. Interestingly, C57BL/6 mice have a hearing defect that is less severe than that of NOD/LtJ mice in the mid‐to‐higher frequency range than CBA/J mice. The p‐values for the NOD/LtJ vs. CBA/J comparisons are represented by asterisks: ^*^ *p* < 0.05, ^**^ *p* < 0.01, ^***^ *p* < 0.001, and ^****^ *p* < 0.0001. Similarly, the p‐values for NOD/LtJ vs. C57BL/6J comparisons represented by # and + are used to represent the p‐values for CBA/J vs. C57BL/6J comparisons. (B) NOD/LtJ mice have a 2 bp frameshift deletion in exon 4 of *Crym*, which is not present in 42 other strains. (C) The full‐length CRYM protein has 313 amino acids, but the NOD/LTJ frameshift deletion occurs within the codon for amino acid 220, which generates a premature termination codon at amino acid 230. (D) The CRYM protein structure (PDB: 4BVA) is shown, and the position of NOD/LtJ‐unique frameshift mutation that disrupts the COOH‐terminal region of CRYM is highlighted along with the location of the NH_2_‐ and COOH‐terminal amino acids. (E) CRYM protein is not present in NOD/LtJ mice. The proteins in lysates prepared from brain tissue obtained from a 5‐week‐old male A/J, CBA/J, or NOD/LtJ mice were separated by SDS‐PAGE and immunoblotted with mouse monoclonal anti‐CRYM or anti‐tubulin antibodies. The blots were scanned after incubation with dye‐labelled goat anti‐mouse IgG. While the lysates have comparable amounts of tubulin, CRYM is not present in the NOD/LtJ lysates.

We next investigated whether Med‐PaLM 2 could analyze a list of murine genes generated by genomic sequence comparison and facilitate finding a novel mouse genetic factor affecting hearing loss among inbred strains. Sixteen inbred mouse strains (of 80 tested) spontaneously develop an age‐related hearing loss by 3 months of age [10]. A murine *Cadherin 23* (*Cdh23 753G→A)* allele, which causes in‐frame skipping of exon 7 [11], was shown to contribute to hearing loss by reducing the stability of a cochlear sensory hair cell bundle protein [12, 13]. We examined our murine SNP database [14, 15] and found an extremely strong correlation between strains with early‐onset hearing loss and *Cdh23^753A^* alleles (Table S2). However, there must be other contributing genetic factors since only a subset of the strains with *Cdh23^753A^* alleles developed early‐onset hearing loss. The age‐related hearing loss of NOD/LtJ mice was of interest because it occurs by 3 weeks of age [10], and we found that NOD/LtJ cochlear sensitivity was dramatically reduced across all frequencies tested. C57BL/6 mice that also have *Cdh23^753A^* alleles develop a much less severe hearing loss at 7 weeks of age (Figure 2A), which is more fully manifested at a later age [10]. Thus, genetic factors other than *Cdh23^753A^* alleles must contribute to the NOD/LtJ hearing loss. To identify them, the genomic sequence of NOD/LtJ was compared with 10 other strains (A/HeJ, AKR, BALB/C, CBA, FVB, LG/J, PL/J, SJL, SM/J, SWR) that maintained normal hearing throughout their life [10], and variant alleles present in NOD/LtJ and any other strain were removed. Med‐PaLM 2 was asked to analyze the 14 genes with high‐impact NOD/LtJ‐specific SNP alleles, using chain of thought (CoT) prompting with self‐consistency, and it identified *Crystallin mu* (*Crym)* as the gene most likely to be associated with hearing loss (Data Files S2 and S3). When Med‐PaLM 2 analyses were repeated 100 times after the input gene order was randomized, *Crym* was identified as one of the top 5 candidates in 98 of the analyses (Table S3). NOD/LtJ mice have a homozygous 2‐bp frameshift deletion allele (rs216145143) within the amino acid 220 codon in *Crym* that was not present in 47 classic inbred strains [14, 15], including the closely related NOR/LtJ strain [16]. This generates a premature termination codon at position 230 (Figure 2B–D), and immunoblotting indicated that the CRYM protein is completely absent from NOD/LtJ tissue (Figure 2E), which results from either degradation of the mutated CRYM protein or its mRNA. Hence, NOD/LtJ mice have a *Crym* knockout. *Crym* was identified by Med‐PaLM 2 because it is highly expressed in a gradient along the length of the mouse cochlea [17], and point mutations in human *CRYM* cause autosomal dominant (AD) non‐syndromic deafness with early onset (DFNA40) [18, 19].

**FIGURE 3:**
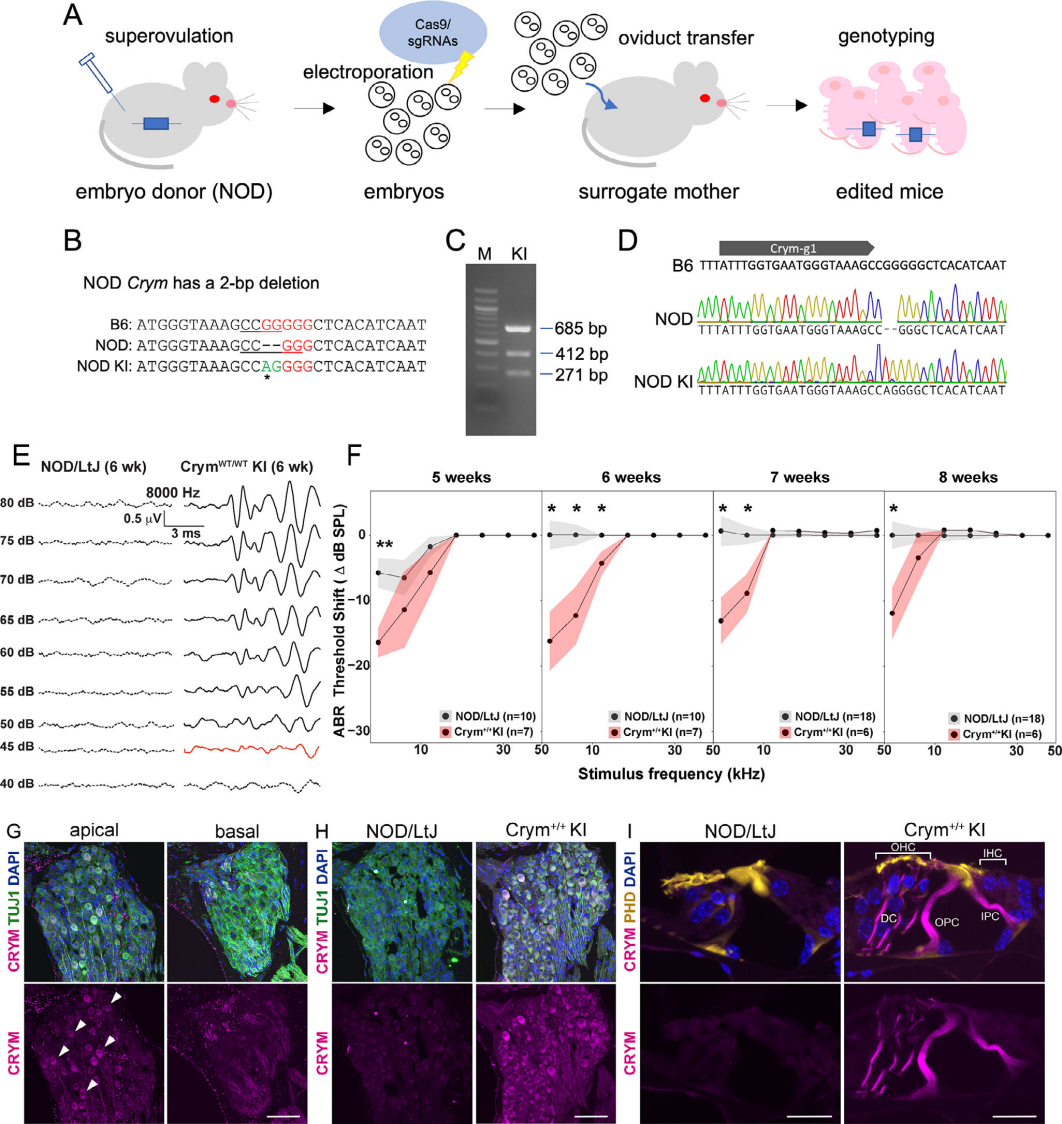
Generation and characterization of NOD KI mice with a CRISPR‐engineered reversion of the *Crym* mutation to wild type (WT). (A) NOD females were super‐ovulated and paired with NOD males to generate fertilized embryos, and pronucleus stage embryos were collected. CRISPR/Cas9, an sgRNA (Crym‐g1), and a single‐stranded donor oligonucleotide (ssODN) were electroporated into the embryos. Healthy embryos were then transferred into the oviducts of pseudo‐pregnant recipient females. (B) sgRNAs were designed to revert the 2‐bp deletion (GG) in NOD with a 2‐bp insert (AG) to restore the reading frame. A silent G to A mutation (indicated by *) was introduced to prevent re‐cutting by inactivating the PAM site of the guide RNA, and this removed an HpaII restriction site, which was used for genotyping of the pups. (C) Pups were screened by PCR amplification of the targeted region followed by HpaII digestion. The HpaII‐resistant 685 bp PCR amplicon indicates that the desired editing occurred, and the 412 and 271 bp bands are from the unreverted NOD *Crym* allele. (D) The 685 bp amplicon from a NOD *Crym* ^WT/−^ KI mouse was gel‐purified and sequenced to confirm that AG was inserted into the region with the 2‐bp deletion. (E) Auditory brainstem response (ABR) waveforms in NOD *Crym* ^WT/WT^ KI mice show improved hearing sensitivity. *Left*: ABR waveforms from an example NOD/LtJ mouse show no detectable auditory response for 8 kHz tone pips from 40 to 80 dB SPL (dashed lines). *Right*: An example ABR waveform from NOD *Crym* ^WT/WT^ KI mouse; the threshold at 45 dB SPL is marked with a red ABR trace. Trace at 40 dB (dashed line) is below threshold. (F) A plot of the ABR threshold shift (Δ dB SPL; NOD/LtJ—*Crym* ^WT/WT^ KI) vs. stimulus frequency (kHz) for 5‐ to 8‐week‐old mice. Each dot represents the mean threshold shift, while the shadows show the SE for NOD/LtJ (gray) or NOD *Crym^WT/WT^* KI (red) mice. NOD *Crym^WT/WT^* KI mice show a significant rescue of hearing sensitivity at the lower range of frequencies (5.66–8 kHz) from 5 to 8 weeks of age. Control background NOD/LtJ mice exhibit profound hearing loss by 6 weeks. Non‐parametric Mann‐Whitney test was used for pairwise comparisons: ^*^ *p* < 0.05, ^**^ *p* < 0.01 (number of mice per condition is shown in the graph legend). (G–I) Cochlear cryostat sections from 10‐week‐old CBA/J mice (G), NOD/LtJ mice, and NOD *Crym* ^WT/WT^ KI mice (H‐I) that were stained with an anti‐CRYM antibody (magenta), DAPI (blue), and an anti‐β‐III tubulin antibody (TUJ1) (G, H, green); or with phalloidin (PHAD) (I, yellow). (G) Spiral ganglion neurons (SGNs) of the apical turn show more CRYM expression than SGNs of the basal turn. Arrowheads indicate CRYM‐positive SGNs. (H, I) Magnified images of the SGN (shown in G) and the Organ of Corti (OC) (shown in I) at the apical turn show CRYM expression in NOD *Crym* ^WT/WT^ KI mice, but not in NOD/LtJ mice. Scale bars: G, H, 50 µm; I, 20 µm. OHC, outer hair cell; IHC, inner hair cell; DC, Deiters’ cells; OPC, outer pillar cell; IPC, inner pillar cell.

To determine whether the *Crym* mutation contributed to hearing loss, knock‐in (KI) mice with a homozygous reversion of the 2 bp *Crym* deletion to wild type were generated on a NOD/LtJ genetic background (NOD *Crym^WT/WT^* KI) (Figure 3A–D). Hearing tests performed weekly from 5 to 8 weeks of age revealed that the KI mice have significantly better hearing at the low‐frequency range (5.66–11.3 kHz) than age‐matched, control NOD/LtJ mice (Figure 3E,F). Histological analysis confirmed that Crym is expressed in the cochlea of NOD Crym^WT/WT^ KI mice but not in NOD/LtJ mice: CRYM is expressed in the spiral ganglion neurons (SGN) and supporting cells of NOD Crym^WT/WT^ KI mice but not NOD/LtJ mice (Figure 3H,I). Consistent with the selective rescue of low‐frequency hearing in the *Crym* revertant, CRYM in WT mice (CBA/CaJ) shows a gradient of expression in cochlear SGNs, with the highest levels detected at the apical turn (Figure 3G; Figure S1A); and this finding is consistent with a previous report using microarrays [17]. While CRYM expression is also detected in the supporting cells and lateral wall, its expression is uniform throughout the cochlear length, which does not explain the low‐frequency hearing rescue after the 2 bp correction (Figure S1A–C). Together, these results indicate that while *Cdh23^753A^ *alleles contribute to hearing loss, the NOD/LtJ *Crym* mutation also contributes to hearing loss through an effect on SGN neurons at the apical turn.

### 2.3 Identification of Genetic Factors for Human Hearing Loss

Our murine genetic analysis methods are analogous to those used to identify causal genetic factors for suspected human genetic diseases. Despite the increased use of genome sequencing, it is difficult to identify the true causative variant for a human genetic disease because (as in the mouse) many thousands of rare variants of unknown significance (VUS) are present in any individual's genome [20, 21, 22, 23, 24]. Therefore, we investigated whether Gemini can help with the identification of causative variants for 20 individuals with hearing loss, each of whom had thousands of VUS in their genome sequence (Table 1). To facilitate these analyses, a computational algorithm (described in Note S1) examined the information contained in multiple databases, and it used multiple criteria to select a subset of the genes with VUS for each patient (range: 375–435). The selected genes were then evaluated by a Gemini 2.5 Pro‐based hypothesis‐generating system and ranked for their potential to cause hearing impairment (HP:0000365, HP:0008527), or deafness (ORPHA:87884) (Data Files S4 and S5). Of importance, the hypotheses and supporting research papers used to rank the genes are output by the system. Then, the Gemini output and allele impact ratings were jointly examined to identify the likely causative genetic factor for each patient (Table 1; Table S5). For example, patient 1 had 16 variants in *Myosin Heavy Chain 14 (MYH14)*, which is an ATP‐dependent molecular motor that regulates cell motility [25]. *MYH14* mutations cause nonsyndromic deafness (DFNA4) with a post‐lingual onset [26, 27], which is consistent with deafness onset at 33 years of age in patient 1. Moreover, one *MYH14 GLU965LYS* variant that occurred at a highly conserved residue was classified as having supportive evidence (PP3) for pathogenicity using the American College of Medical Genetics and Genomics (ACMG) criteria [28], and protein structure modeling indicated that this variant could cause a significant functional change in MYH14 protein structure (Figure S2 and Note S2). Similarly, a *GJB2 Arg143Gln* variant was identified as the likely genetic cause for hearing loss in patient 3 because: *GJB2* was the second highest ranked gene by Gemini, the *Arg143Gln* variant was rated as strongly pathogenic; *GJB2* mutations cause an autosomal dominant (**AD**) form of deafness 3A (DFN3A) [29]; this mutation was associated with autosomal dominant deafness in several clinical studies [30, 31], and to have a dominant effect in functional assays [32]. Other causative *GJB2* mutations were identified in patients 6 and 9 (Table 2). A *Tyrosinase (TYR)* variant (*Arg422GLN*) was identified as the causative mutation for patient 4, who had both albinism and deafness. *TYR* mutations are mainly associated with Oculocutaneous albinism (OMIM 203100 and 606952), which is inherited in an autosomal recessive pattern. However, ocular albinism with congenital sensorineural hearing loss has been reported in the NIH Genetic Testing Registry (ID: CN028925), and the father of patient 4 father had this allele and deafness (Table S5). As another example, patient 7 has two missense variants in α‐tectorin (*TECTA*: *Val830Met* and *Tyr942Cys)*, and *Tyr942Cys* was rated as likely pathogenic. TECTA is a major component of the membrane that covers cochlear hair cells, which is required for sound amplification by outer hair cells. *TECTA* mutations are associated with AD hearing loss (DFNA8/12) in multiple populations [33, 34, 35]. For patient 12, Gemini identified a missense variant in *Gasdermin E* (*GSDME*) as the likely causative genetic factor because *GSDME* mutations cause an AD progressive sensorineural hearing loss (DFNA5) [36, 37, 38]. *GSDME Leu485Arg* had the second‐highest allelic impact rating of the 468 selected genes, and modeling predicts that this variant will cause a significant change in a key functional domain (Figure S3). The GSDME COOH‐terminal domain, which is encoded by exons 8–10, regulates the necrosis‐inducing activity of GSDME [39], and alterations in this domain promote cochlear hair cell apoptosis [37].

**TABLE 1:** The AI pipeline identified potential causative genes for hearing loss for 19 of the 20 patients whose data were analyzed. The total number of variants in each patient, the number of genes selected for analysis, and the identified causative gene (and variant allele) are shown for each patient. The LLM gene rank, the allele impact rank, and the Combined Annotation‐Dependent Depletion (*55*) (CADD PHRED) score are shown. The association of the gene with hearing loss identified by the AI (Hearing Association) does not necessarily provide a diagnosis for the patient. Of note, a CADD PHRED ranking >20 (or >27) indicates that the impact of that allele is within the top 1% (or 0.1%) of all possible variants in the genome [40]. A gene candidate was not identified for patient 19 (unknown), and two different alleles in one gene were identified for patient 10. NA, CADD PHRED score not available for frameshift variants. Two predictions that the clinical experts did not agree with are indicated (*); second variants were identified within the genes for patients 7 and 9 (&). The predicted variants for patients 14 and 20 were ruled out after family members were evaluated, and other variants are being pursued for patient 13 ($) (see Table S5).

	# Variants	Genes Selected	Gene (Var)	LLM Rank	Allele Impact	CADD PHRED	Hearing Association
1	94,117	409	*MYH14* p.Glu965Lys	66	1	31	Deafness, autosomal dominant 4A/4B
2	79,455	435	*ACTG1* p.Glu241Lys	45	3	25.8	Autosomal dominant with hearing loss
3	73,352	435	*GJB2* p.Arg143Gln	2	78	27.7	Deafness, autosomal Dominant 3A, 1A (DFNA3A)
4	90,295	406	*TYR* p.Arg422Gln	41	171	24.7	Albinism, oculocutaneous, 1A or 1B (OCA1A or OCA1B)
5	97,686	406	*PTPN11* p.Thr468Met	19	102	26.7	Noonan Syndrome 50% have hearing loss
6	26,559	425	*GJB2* p.Leu79CysfsTer3	2	37	NA	Deafness, autosomal recessive 1A, DFNB1
7	27,935	439	*TECTA* p.Tyr1942Cys	2	47	29.7	Deafness, autosomal recessive B21, DFNB21^&^
8	26,457	412	*KCNQ4* p.Gly285Ser	2	27	32	Deafness, autosomal dominant, 2A DFNA2A
9	25,239	424	*GJB2* p.Phe191Leu	2	50	29.6	Deafness, autosomal recessive 1A, DFNB1A^&^
10	92,648	375	CEP250^*^ p.Arg1385His p.Arg222His	*10*	*3* *5*	*22.7* *21.7*	Deafness, autosomal Recessive, Cone‐Rod Syndrome
11	94,648	377	*P2RX2* p.Arg46His	*7*	2	28.2	Deafness, autosomal dominant DFNA41
12	75,590	397	*GSDME* p.Leu485Arg	73	2	27.2	Deafness, autosomal dominant DFNA5
13	93,159	381	*COL11A1^$^* p.Phe1698Leu	10	3	26.8	Stickler syndrome, type II; Deafness Autosomal dominant
14	87,456	387	*MYO3A^$^* p.Val369Ile	4	375	NA	can cause AD hearing loss
15	90,930	407	MYO7A^*^ p.Asn1586Ser	3	273	22.9	Autosomal dominant or recessive deafness DFNAB7/B11
16	90,530	407	*TMC1* p.Asp684His	2	57	28.8	Autosomal recessive deafness (DFNB7/B11)
17	83,886	435	*POU4F3* p.Thr94Ile	2	2	23.1	Autosomal dominant deafness DFNA15
18	71,587	412	*CEACAM16* p.Gly289Arg	4	125	25.7	Autosomal dominant deafness, DFNA4B
19	84,096		UNKNOWN				
20	89,539	425	*MYO6^$^* p.Leu1283ValfsTer39	4	2	NA	Autosomal dominant deafness DFNA22

**TABLE 2:** Comparative analysis of the ability of CADD assessment of allelic impact, Exomiser rankings, or the Gemini LLM without and with prompts that include the CADD assessments of allelic impact to identify genes with the causative genetic factors for six patients with rare genetic diseases. The patient number and the symbol for the causative gene for each patient are shown. NR, not ranked; *the prompt did not contain the CADD ranking due to the inability of CADD to rank frameshift deletions.

#	Gene	CADD	Exomiser	LLM	LLM+CADD
1	*IRAK4*	2	27	2	1
2	*ODC1*	NR	1	25	14*
3	*CDLK5*	NR	1	39	1*
4	*ADSS1*	2	25	2	2
5	*RHOBTB2*	1	5	7	2
6	*POLR3A*	2	2	8	1

The genetic factors identified by the AI pipeline were evaluated by the otolaryngologist (CWW), taking care of these 20 patients, and a clinical geneticist (CYT) with expertise in hearing loss. These experts fully (n = 15) or tentatively (n = 3) agreed with 18 of the 20 predictions. Hence, this AI pipeline identified genetic factors for 15 subjects and probable variants for 3 subjects, which were concordant with the clinician's assessments. Although they did not agree with the predictions for patients 10 and 15, alternative candidates were identified by re‐evaluating the LLM and allelic impact rankings (Table S5). Also, subsequent follow‐up revealed that the identified genetic variants for patients 13, 14, and 20 were not causative, and other variants are being pursued using AI pipeline output (Table S5). While the AI pipeline results can be improved by changing the information provided in the prompts (see below), these results demonstrate that it has the potential to assist in identifying causative genetic factors for hearing loss.

### 2.4 Rare Genetic Diseases

**FIGURE 4:**
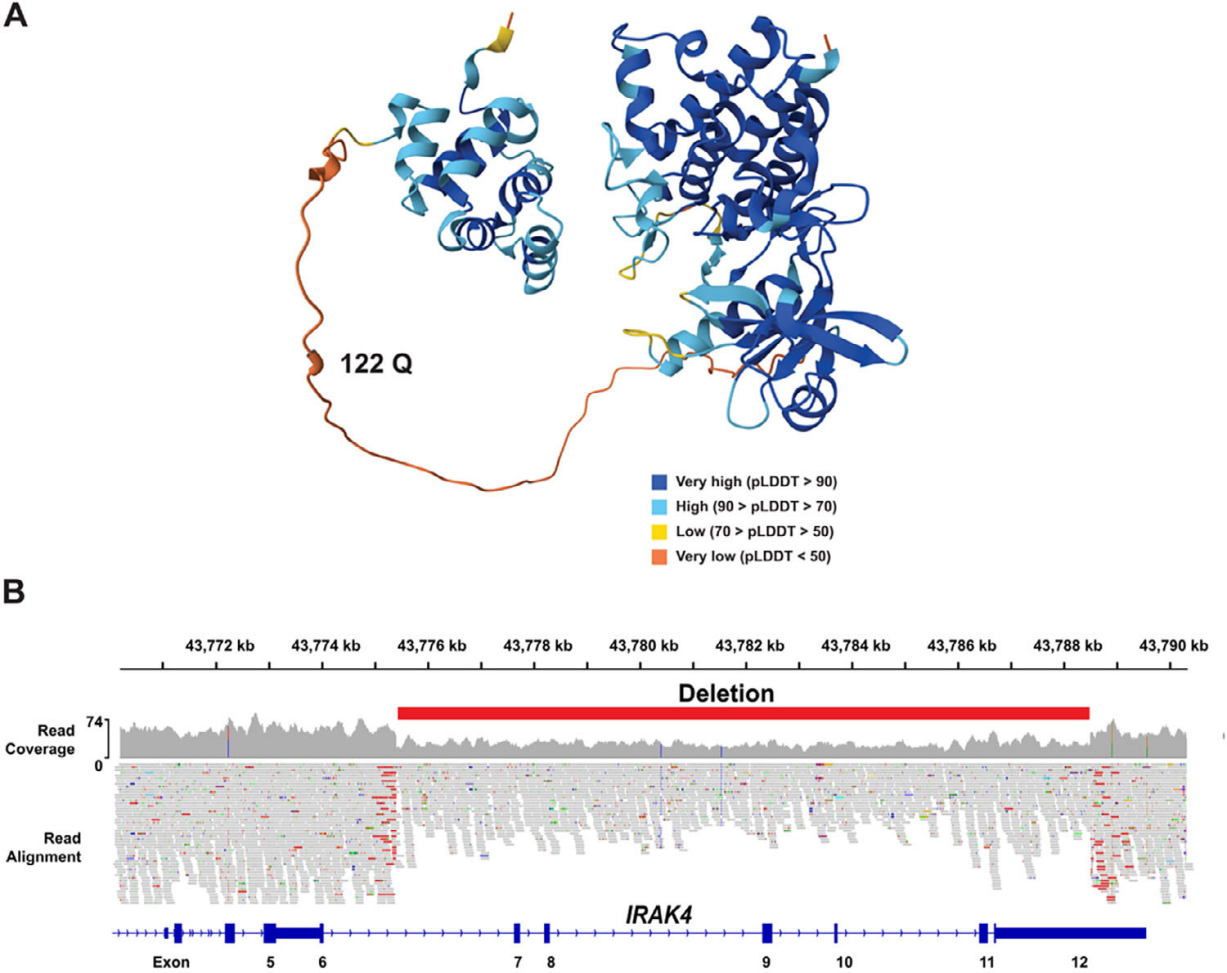
Bi‐allelic *IRAK4* mutations cause a genetic syndrome. (A) The IRAK4 protein structure as predicted by AlphaFold ^45,^ with the position of the *Q122X* mutation in Genetic Disease Patient 1. AlphaFold produces a confidence score (between 0 and 100) for each residue (pLDDT). Regions with a pLDDT<50 appear unstructured in the purified protein. The introduction of a premature termination codon at amino acid 122 will severely disrupt the function of IRAK4 and could prevent *IRAK4* mRNA from being translated due to nonsense‐mediated decay. (B) As visualized using the Integrative Genomics Viewer, this patient also has a 13.1 kB deletion (Chr12:g.43775404_43788500 (GRCh38)) that begins in intron 6 of *IRAK4*, and it removes exons 7–11 and part of exon 12.

We next investigated whether Gemini could identify causative variants for individuals with suspected rare genetic diseases, which present with multiple, complex clinical features. For the hearing loss patients, assessing the impact of the mutated allele on the protein sequence was critical for identifying the causative genetic factors. As discussed below, a comparative analysis indicated that provision of variant impact rankings, which were determined by the Combined Annotation–Dependent Depletion (CADD) program [40], with the prompt improved our system's ability to identify causative genetic factors (Table 2). Therefore, the analysis pipeline for the genetic disease patients included the CADD variant impact assessment with the gene list and human phenotype ontology (HPO) terms that were provided to Gemini (Data File S6). For each of these patients, the genetic factor identified by Gemini was verified by genetic experts caring for them. Genetic patient 1 experienced multiple infections at a young age, including a cryptogenic meningitis, and later had additional unexplained multi‐organ inflammation (uveitis, pericarditis, myocarditis). The 232 genes with candidate variants identified by genomic sequence analysis, the 14 HPO terms for the different clinical features, the input query, and the output of our system are shown in Data File S7. The LLM analysis identified candidate genes that were most likely to contain a causative genetic variant for the observed clinical features (Data File S8). *Interleukin‐1 Receptor‐Associated Kinase 4* (*IRAK4)* was the highest‐ranked gene by Gemini. *IRAK4* was the only highly ranked gene with a high‐impact mutation: a stop codon at Q122 (c.364C>T) truncated the 460 amino acid IRAK4 protein (Figure 4A). Subsequent analysis of this patient's genomic sequence identified a 13.1 kb deletion that removed exons 6–11 and part of exon 12 of *IRAK4 (*Figure 4B). Hence, this patient has high‐impact bi‐allelic mutations in a serine‐threonine kinase that triggers the release of proinflammatory cytokines (IL‐1, IL‐8, IL‐33, and type 1 interferons) required for pathogen defense (*37‐39*). Consistent with the clinical features, IRAK4‐deficient individuals are susceptible to pyogenic infections at an early age [41, 42, 43], and they develop encephalitis [44] and other inflammatory conditions later on.

Genetic patient 2 is a young boy with a history of global developmental delay since infancy, short stature, sagittal synostosis, unilateral nasal airway blockade, hypotonia, and hypotonicity in the newborn period. His exam was notable for sparse hair, down‐slanted palpebral fissures, facial hypotonia, overlapping toes, and small second and fifth nails of the hands. Gemini evaluated the selected gene candidates and the 34 HPO terms describing the clinical features, and it identified 47 candidate genes (Data File S8), but only two had high‐impact alleles. A frameshift deletion (Pro438Argfs*9) in *Ornithine decarboxylase 1* (*ODC1*) was identified as the likely cause of this unusual constellation of abnormalities because it altered the sequence of 9 amino acids (beginning at Pro438) before causing a truncation of the 462 amino acid ODC1 protein. Mutations at the 3’ end of *ODC1* cause a neurodevelopmental disorder [the Bachmann‐Bupp syndrome] that is characterized by developmental delay, hypotonia, and non‐congenital alopecia [45, 46, 47, 48]. ODC1 encodes the rate‐limiting enzyme for polyamine production, which is essential for embryonic development and cell proliferation [49]. A C‐terminal truncation mutation increases ODC1 activity by preventing its interaction with proteins that mediate its proteasomal degradation (Figure S4); this genetic effector mechanism produces skin wrinkling, enhanced nail growth, and hair loss in mice [50, 51].

Genetic patient 3 is a young girl who presented with an infantile‐onset developmental epileptic encephalopathy with associated intellectual disability and autism spectrum disorder features. Seizures were first noted at 7 months of age; she walked at 13 months and developed a pincer grasp at age two. She is non‐verbal. Her exam is also notable for a high palate, long eyelashes, a café au lait spot, joint hypermobility, small hands and feet, and a small head; and a normal brain MRI at age 7 months. Of the genes with variants and 14 HPO terms evaluated by Gemini (Data File S8), 48 gene candidates were identified (Data File S6), but only two had high‐impact frameshift variants. Based upon the clinical features, a Cyclin‐dependent kinase‐like 5 (*CDKL5)* frameshift variant (ENST00000623535.2:c.2828_2829del: p.(Arg943Asnfs*11)) was identified as the likely genetic cause (Data File S8). This variant altered the sequence of 11 amino acids beginning at Arg943 before truncating the 960 amino acid protein. CDKL5 is a serine threonine kinase that is highly expressed in the brain, and it modulates the postsynaptic localization and composition of N‐methyl‐D‐aspartate receptors [52]. Its long C‐terminal sequence regulates the nuclear to cytoplasmic shuttling of CDKL5 [53], which is how this C‐terminal frameshift mutation could reduce CDKL5 enzyme activity (Figure S5). Consistent with this patient's abnormalities, CDKL5 deficiency disorder is an X‐linked condition characterized by global developmental delay, intellectual disability, seizures, and autistic features, which are commonly reported, as well as hand stereotypies [54, 55, 56].

Genetic disease patient 4 is an adult male with a history of progressive proximal muscular weakness beginning at age 24, and an EMG showed a possible myositis that did not respond to prednisone treatment. The patient also had an elevated creatine phosphokinase, an echocardiogram showing an ejection fraction of 44%, a muscle biopsy showing possible myotilinopathy and dysferlinopathy, and proximal leg muscle atrophy on MRI. The patient later developed facial muscle weakness and had difficulty rising from a seated position. Gemini evaluated 9 HPO terms and identified an *Adenylosuccinate synthetase 1 (ADSS1 Asp261Asn*) mutation as the likely cause because it is within a protein domain where previously identified causative mutations for *ADSS1*‐linked myopathy were located (*56*) (Data File S8). ADSS1 catalyzes the conversion of IMP to AMP, which is essential for energy production in muscle. Consistent with this patient's presentation, *ADSS1* myopathy is a progressive myopathy with an onset in young adults, and muscle histopathology may include fiber splitting and focal fibrosis [57]. Since ADSS1 myopathy is an autosomal recessive disease, Gemini recommended looking for additional *ADSS1* mutations in this subject. Genetic patient 5 is a young male with global developmental delay that was first noted in early infancy, myoclonic epilepsy, central hypotonia, and gastrointestinal symptoms including reflux and constipation. Gemini evaluated the 9 HPO terms, and the second‐highest‐ranked gene had a missense variant (*Arg461His*) in *RHOBTB2. RHOBTB2* mutations are associated with early onset epilepsy, developmental delay, and gastrointestinal symptoms, which were noted in a small percentage of these patients [58, 59]. This patient's mutation is within a domain where other disease‐causing *RHOBTB2* mutations are located, and it is important for RHOBTB2 function [59].

Genetic disease patient 6 is a young girl with a history of dysmorphic features, hypotonia, developmental delay, and developmental regression. While she had head control at two months and started taking solids at 6 months, she subsequently lost these skills and has been feeding‐tube dependent. She also has late eruption of teeth with early deciduous tooth loss, a laryngeal cleft, and pulmonary insufficiency with oxygen dependence after multiple pneumonias. She had premature adrenarche at age 3 and has had two episodes of pancreatitis. An MRI had increased T2 signal in the basal ganglia and decreased white matter volume. She experienced a febrile seizure in toddlerhood. Her exam is notable for synophrys, low anterior hairline, a patch of hair at her right temple, an area of coarser hair at the posterior scalp, overlapping toes, prominent lower lip, prominent mandible, mild pectus excavatum, and long eyelashes. Gemini identified missense (*Ser467Leu*) and splice site (ENST00000372371.3:c.1771‐7C>G:p.?) mutations in *RNA polymerase III subunit alpha (POL3RA)* as most strongly associated with these abnormalities. *POLR3A* encodes an essential RNA polymerase III subunit for transcribing tRNAs and rRNAs. Compound heterozygous mutations are causative of a neurodegenerative disorder featuring deficient cerebral myelin formation (childhood‐onset hypo‐myelinating leukodystrophy 7, HLD7) [60, 61] with clinical features that align with the patient's profound neurological symptoms and the observed MRI findings.

### 2.5 Comparative Performance

We posed four scenarios of increasing complexity selected from those analyzed above to four LLMs: OpenAI GPT4.5, DeepSeek R1, OpenAI mini‐03‐hi, and Claude Sonnet 3.7. The first two scenarios analyzed mouse candidate genes for cataract and hearing loss, while the third and fourth scenarios were the data from Genetic disease patients 1 and 2. The prompts used for these comparisons were the same as those used above, and the output of each LLM is shown in Data File S9. In brief, OpenAI GPT4.5 provided wrong answers for all four scenarios; DeepSeek R1 and OpenAI mini‐03‐hi provided correct answers for both mouse scenarios but wrong answers for both genetic disease patients; and Claude Sonnet 3.7 provided correct answers for one of the two mouse scenarios and for one of the two genetic disease patients. Although Gemini 2.5 performed better than the other LLMs tested, analysis of only four cases does not fully evaluate the relative capabilities of the different LLMs. We next compared the relative performance of using the CADD allele impact rankings [40]; the rankings obtained using Exomiser [62], which is a suite of algorithms used for prioritizing genes or variants for diagnosing genetic diseases; or Gemini with clinical prompts with or without the CADD allelic rankings. The ability of each method to analyze the list of genes and variant information (Data File S6) and identify causative genes for the six genetic disease patients was assessed. CADD analyses ranked the causative gene as the first or second highest ranked gene for four genetic disease patients, but it could not prioritize two causative genes because of its inability to analyze frameshift mutations. Exomiser ranked the causative genes for two genetic disease patients as having the highest association with their clinical condition; two causative genes were ranked fifth, but the causative genes for two subjects were ranked 25th by Exomiser, which meant they were unlikely to be considered. Gemini analyses performed without the CADD rankings never identified a causative gene as the highest‐ranked gene, but two causative genes had the second‐highest ranking, two had the seventh or eighth‐highest ranking, and two were ranked 25th or 39th. In contrast, Gemini analyses with CADD information identified the causative gene as the top‐ranked gene for 5 of the 6 genetic disease patients. The causative gene for one genetic disease patient was ranked 15th because a frameshift variant could not receive a CADD ranking (Table 2). Our results also compare favorably with those obtained using a recently developed graph neural network‐based (GNN) method used for diagnosing subjects with rare genetic diseases, which the authors showed performed better than 10 other available gene ranking methods [63]. However, when that GNN analyzed gene lists containing a small number of expert curated genes (*n* = 13 ± 8), the causative gene was the top‐ranked gene only 40% of the time [63]. When it evaluated lists with a larger number of genes (*n* = 223 ± 244) generated by computational analysis of variants (*n* = 223 ± 244), the causative genes were the top‐ranked gene only 21% of the time and were among the top 10 ranked genes 48% of the time. Moreover, the hypothesis and supportive evidence output by Gemini provide a rationale for its gene rankings, which enables clinicians or investigators to better evaluate the results. In summary, an AI pipeline using the CADD variant rankings and the Gemini LLM improved our ability to correctly assign genetic causation, and the improved performance was especially apparent when multi‐faceted clinical scenarios were analyzed.

### 2.6 Statistical Analysis

For the mouse hearing experiments, the ABR and DPOAE thresholds were plotted either as absolute values (Figure 2) or as differences between Nod/LtJ and NOD *Crym* ^WT/WT^ KI mouse lines (Figure 3) to illustrate the degree of hearing rescue (i.e., threshold shifts). No additional transformation of the original traces or exclusion of outliers was performed. Data are presented as mean ± SEM, with sample sizes ranging from 6 to 18 mice per group. For each mouse, hearing thresholds were calculated as the average of the thresholds obtained from both ears at each tested frequency. Differences in hearing thresholds between mouse lines were assessed using non‐parametric Mann–Whitney tests, which were performed pairwise for each frequency tested. All plots were generated, and statistical analyses were conducted using R.

## 3 Discussion

We demonstrate that LLMs can assist in identifying causative genetic variants for mouse and human hearing loss, and for humans with rare genetic diseases. Based upon Med‐PaLM 2 output, we found that the spontaneous hearing loss of NOD/LtJ mice has (at minimum) a digenic origin where the NOD/LtJ *Cdh23* and *Crym* frameshift alleles jointly contribute. This digenic model explains four features of the hearing loss that develops in NOD/LtJ and in several other inbred strains. (i) Some inbred strains with *Cdh23^753A^* alleles do not develop a hearing loss. (ii) The time of onset and the severity of the hearing loss in strains with *Cdh23^753A^* alleles are variable. For example, the NOD/LtJ hearing loss occurs earlier and is more severe than in C57BL/6 mice, which have the *Cdh23^753A^* allele but do not have the NOD/LtJ *Crym* frameshift allele. (iii) Reversion of the *Cdh23^753A^* allele to wildtype only partially rescued hearing loss in a NOD substrain, and the rescue effect was more pronounced for low‐frequency sounds [64]. (iv) A *Crym* knockout mouse on a 129 Sv background had normal hearing [65], but this results from it having the *Cdh23^753G^* allele associated with maintenance of normal hearing [10]. These four features, along with the partial effect of the reversion of the *Crym* mutation in NOD/LtJ mice, indicate that at least two genetic factors, which are exon‐skipping and frameshift mutations in *Crym* and *Cdh23*, respectively, contribute to the NOD/LtJ hearing loss. This has important implications for understanding the genetics of hearing loss. In contrast to the many different types of human hearing loss with a monogenic cause, the NOD/LtJ hearing loss results from the contributions of at least two genetic factors.

We also demonstrate that general‐purpose LLMs can be used to identify the genetic basis for human diseases, especially when they are augmented with tools enabling access to external biomedical knowledge. Our retrieval and grounding pipeline directly addresses the interpretability of identified causative variants by citing evidence from the biomedical literature. The reasoning output of our system provides a straightforward means for human researchers to understand and verify the inner thinking mechanism of our system on this task. These results indicate that AI‐based methods could subsequently be used to efficiently determine the genetic basis for the estimated ∼350 M people globally [66] with suspected genetic diseases [67] or rare undiagnosed syndromes [68]. More broadly, since an increasing number of individuals will have their genomes sequenced, AI‐based methods could improve healthcare for billions of individuals by providing access to precision genomic health. Automated methods for the interpretation of clinical and genetic data will allow people to access definitive therapies in a timely manner. Furthermore, enabling individuals to better understand the impact of their genetic determinants could have a far‐reaching and transformative impact on public health: medical practice could shift from its current focus on disease treatment toward disease prevention; customized plans for disease prevention could be developed based upon genetic factors.

Our findings also indicate how LLM genetic discovery performance can be improved. First, by analyzing LLM predictions, which were not associated with an analyzed trait, we identified at least two factors that contribute to the false associations generated by LLMs (which are referred to as ‘*hallucinations*’). (i) The tokenizer (i.e., which breaks down a piece of text into tokens) has difficulty analyzing gene symbols, which consist of letters (often acronyms) and numbers. Tokenizers can incorrectly assign gene symbols to other genes (or words), which generate false associations based upon analysis of the related genes (or words). For example, an input gene (*Med1)* was falsely associated with hearing loss because mutations in another gene (*MED12)* cause congenital hearing impairment [69]. (ii) The next word prediction objective used to train the LLM sometimes leads to spurious associations between genes and phenotypes, which do not have causal relationships, due to their co‐occurrence within the same context window. In one case, a spurious association was caused by a series of >40 abstracts, each covering a different topic, that were published together as a conference summary. In another case, a diverse set of phenotypes exhibited by an inbred strain (which included hearing loss) was summarized in the introduction to a paper, which characterized the effect of a gene knockout on an unrelated phenotype [70]. Second, a scalable LLM‐based AI system for novel genetic discovery and diagnosis can be designed to overcome the current limitation of the knowledge encoded in LLMs. For example, an AI system could be integrated into an agentic framework that could not only access and process information from the biomedical literature but would also dynamically utilize specialized AI tools as needed. For instance, the LLM could leverage models like AlphaMissense [71] or AlphaProteo [72] for analysis of mutation effects upon protein structure, or could use search‐based retrieval augmentation to provide supporting evidence (from other genetic data or the published literature) and remove false positive associations. Also, by using high‐quality domain‐specific data, process supervision, and providing granular feedback to the AI system, we can further enable the AI system to analyze regulatory elements, protein‐protein interactions, or non‐coding RNA functions, which in turn will further expand the range of genetic hypotheses that these models can develop. The integration of diverse AI capabilities would enable a more comprehensive and nuanced understanding of complex genetic relationships, leading to more accurate and insightful predictions.

## Author Contributions

G.P. and K.M.S. formulated the project. T.T. and G.P. wrote the paper with input from all authors. J.B., M.W., D.B., T.T., Z.F., S.S., M.D., H.M., C.Y.T., C.C.W., and Z.C. generated experimental data. T.T., K.S., W.L., Z.F., W.R., Z.F., A. Palepu., A. Pawlosky, J.G., S.M., H.R.M., J.B., S.S., M.D., K.M.S., and G.P. analyzed the data. WL developed the gene/variant selection methods and performed the protein structural analyses. T.T., K.S., A. Palepu., K.K., A. Pawlosky, J.G., A.K., and V.N. developed the LLM and the techniques for enabling its genetic discovery applications. All authors have read and approved the manuscript.

## Code Availability

Both Med‐PaLM 2 (MedLM) and Gemini 2.5 Pro are available via public API. For reproducibility, we documented technical deep learning methods while keeping the paper accessible to a clinical and general scientific audience. Our work builds upon PaLM and Gemini, for which technical details have been described extensively, and our institution has open‐sourced several related LLMs to further the development of research methods in the field https://ai.google.dev/gemma and https://huggingface.co/google/flan‐t5‐xl.

## Conflicts of Interest

The Stanford University Medical School and National Taiwan University College of Medicine authors have no competing interests. T.T., K.S., J.G., A.K., K.K., V.N., A. Palepu, and A. Pawlosky. are employees of Alphabet and may own stock as part of their standard compensation package.

## Supporting information




**Supporting File 1**: advs74268‐sup‐0001‐SuppMat.docx.

**Supporting File 2**: advs74268‐sup‐0002‐SupplementalDataFile1.docx.

**Supporting File 3**: advs74268‐sup‐0003‐SupplementalDataFile2.docx.

**Supporting File 4**: advs74268‐sup‐0004‐SupplementalDataFile3.docx.

**Supporting File 5**: advs74268‐sup‐0005‐SupplementalDataFile4.xlsx.

**Supporting File 6**: advs74268‐sup‐0006‐SupplementalDataFile5.xlsx.

**Supporting File 7**: advs74268‐sup‐0007‐SupplementalDataFile6.xlsx.

**Supporting File 8**: advs74268‐sup‐0008‐SupplementalDataFile7.docx.

**Supporting File 9**: advs74268‐sup‐0009‐SupplementalDataFile8.xlsx.

**Supporting File 10**: advs74268‐sup‐0010‐SupplementalDataFile9.docx.

**Supporting File 11**: advs74268‐sup‐0011‐SupplementalDataFile10.docx.

## Data Availability

The mouse SNP allele data is available at the Mouse Phenome Database (GenomeMUSter https://mpd.jax.org/genotypes). The Taiwan gene and variant lists are available under an open‐source license (CC 4.0) at https://redivis.com/datasets/enk5‐0xc36zec3?v=1.0.
