# Supplementary material for: Genetic Diagnosis and Discovery Enabled by Large Language Models
Source: Adv Sci (Weinh). 2026 Feb 8;13(22):e18656. doi: 10.1002/advs.202518656 (PMC13088295; doi:10.1002/advs.202518656)
Supplement: Supplementary file 1 — Supporting File 1: advs74268‐sup‐0001‐SuppMat.docx. [file ADVS-13-e18656-s006.docx]

Supplemental Information

**Genetic Diagnosis and Discovery Enabled by Large Language Models**

*Supplemental Note 1: Variant selection for identifying the genetic basis for human hearing loss*. We had no information about the patients other than that they developed a hearing loss, and each patient had many VUS (range 25K – 97K). While many different computational programs can assess variant pathogenicity ^[1],[2],[3]^; most evaluate a limited data set and have low specificity, which results in overprediction of missense changes as deleterious [4]. Therefore, we developed a five-component computational process to select and rank high impact variants within genes that will subsequently be analyzed by the LLM. (i) We directly selected variants within genes that were annotated as 'deaf' or 'hearing loss' by searching three databases (database column in parenthesis): Online Mendelian Inheritance in Man [5] (MOI_condition), UniProt [6] (UniProt_diseaseNames), and ClinVar (PathogenicDB_Region) [7]. The selected genes associated with hearing (n=164) were 2.3% (range 4-14%) of the total number of genes (n=7171) selected for these 20 patients. Also, the search term can be modified when other diseases are analyzed. (ii) To ensure that we did not omit a potentially causative genetic factor, we also selected variants using the ClinVar database [8] that were annotated as Pathogenic or Likely_Pathogenic (CLNSIG column), or were either ‘reviewed by expert panel’ or a 'practice guideline' (ClinVar_CLNREVSTAT column). (iii) Since the American College of Medical Genetics and Genomics (ACMG) has developed guidelines to aide in interpreting variant pathogenicity [9], we also selected variants annotated in the ACMG database (ACMG_Rules) as strongly (PS) or very strongly (PVS) pathogenic, or the variants that contained multiple tags (i.e., containing '|'). (iv) The variants selected by these methods were filtered using Variant Effect Predictor (VEP) program [10] to select those that met the following criteria: column VEP_Impact (HIGH,MODERATE), Otherinfo_DP>10, Otherinfo_ADalt+Otherinfo_ADre>10, DANN_DannScore>0.8, and CADD_PHRED score>20. These steps resulted in the selection of an average of 377 genes for each patient. (v) The variants were ranked using the Combined Annotation–Dependent Depletion (CADD) program [11], and the selected variants with missing CADD values were placed at the bottom of the list. CADD was used to assess allelic importance because it is a machine learning-trained model that integrates over 60 different assessments into a single measure for each variant [11-12]. It assesses a range of information including allelic diversity, genome context, functional annotations, pathogenicity, disease severity, experimentally measured effects and complex trait associations. The raw score is transformed into a log_10_-derived ranked score (PHRED) that is based upon the scores obtained from assessing ~9B variants (i.e., 3 per site) in the genome. A PHRED score >20 indicates a raw score in the top 1% of all possible variants. The scaled CADD PHRED score was used to assess pathogenicity since it compares each allele to all possible variants in the genome; and it is not possible to establish a binary cutoff (pathogenic vs benign) using the raw score [11].

*Supplemental Note 2: Structural Modeling*. In figures S2-S5, Alphafold3 modelling [13] is used to predict the effect of mutations on protein structure and protein interactions. However, Alphafold3’s accuracy for predicting the interaction model for proteins with mutations has yet to be verified. Hence, we only compare the non-mutated and mutated models developed by Alphafold3 to develop hypotheses about the structural effect of mutations that are predicted to be pathogenic. The precise structure of a mutated protein does require experimental determination.

**Methods**

*Animals and Experimental Design.* All mouse procedures were approved by the Institutional Animal Care and Use Committee at the Stanford University Medical School; they were conducted in accordance with the National Institute of Health Guide for Care and Use of Laboratory Animals, Eighth Edition; and the results are reported according to the ARRIVE guidelines [14]. Male NOD/LtJ, C57BL/6 and CBA/J mice were purchased from Jackson Laboratories (Sacramento, CA, United States). CBA/J mice were used as a control since they maintain good cochlear sensitivity through most of their adulthood. Cochlear function was examined by measuring auditory brainstem responses (ABR) and distortion product otoacoustic emissions (DPOAE) at 7 weeks of age (N=7 for NOD/LtJ, N=14 for C57BL/6, and N=10 for CBA/J).

*Cochlear function testing***.** Cochlear function testing was performed using previously described methods [15]. In brief, mice were anesthetized with ketamine (120 mg/kg) and xylazine (12 mg/kg) that was administered intraperitoneally. A custom acoustic system consisting of two speakers (MF1, TDT, Alachua, FL, United States) and a microphone (ER-10B+, Ethymotic Research, Elk Grove Village, IL, United States) coupled to a probe tube was used to measure pressure near the mouse eardrum. DPOAEs were measured as ear canal pressure in response to two tones presented into the ear canal (*f1* and *f2*, with *f2/f1*=1.2) at half-octave steps, from *f2* = 5.6 to 45.2 kHz, and in 5 dB intensity increments from 10 to 80 dB sound pressure level (SPL). ABR responses to 5-ms tone pips were measured between subdermal electrodes (placed adjacent to ipsilateral pinna, at the vertex, and near the tail), amplified 10,000 times through an amplifier (Medusa4Z, TDT, Alachua, FL, United States) and filtered (0.3-3.0 kHz). For each frequency and sound level assessed, 512 responses were recorded and averaged using the BioSigRZ software run on a RZ6 Multi-I/O Processor (TDT, Alachua, FL, United States). ABR waveforms stacked from lowest to highest SPL were visually inspected to define the threshold as the first level at which a repeatable wave I was detected. In the absence of an ABR or DPOAE response, 85 dB SPL was chosen as a threshold because it was 5 dB above the highest sound pressure level tested [16].

*Whole genome variant calling and annotation for mice.* The pipeline used to identify and annotate the SNPs and structural variants in the database was previously described [17]. In summary, the raw reads of the genomic sequence for 53 inbred strains was aligned to the reference genome (GRCm38) using bwa [18] and variants were discovered using BCFtools [19]. Each variant was annotated using Ensemble Variant Effect Predictor (VEP) [20]. For this analysis, a variant was retained if it had a high-impact consequence on any transcript, as determined by VEP. To identify unique high-impact variants in the NOD/LtJ strain, we subtracted variant alleles that were also present in any of 10 other strains (A/HeJ, AKR, BALB/C, CBA, FVB, LG/J, PL/J, SJL, SM/J, SWR) that maintained normal hearing throughout much of their life [21]. VEP defines high-impact variants as those causing transcript ablation, splice acceptor/donor variants, stop gain or stop loss, frameshifts, start lost, or transcript amplification (Supplemental Data File 2). Alleles within genes encoding polymorphic genomic markers or immunoglobulin variable regions, or within splice sites were removed. Then, the 14 genes with alleles that caused a frameshift, stop loss, or stop gain were selected for analysis by Med-PaLM 2.

*Prompting strategies for Med-PaLM 2*. To assess the capability of Med-PaLM 2 to identify a causative genetic factor for previously studied biomedical traits, Med-PaLM2 was zero-shot prompted to select the most likely causative gene from a set of genes that were identified previously through computational genetic analysis of mouse GWAS data. We evaluated the model’s answers for the six biomedical traits because the correct causative gene for each phenotype was validated in previous studies. The prompts and model outputs for all 6 test examples are shown in Supplemental Data File 1.

To assess the ability of Med-PaLM 2 to facilitate novel genetic discovery, Med-PaLM 2 was used to identify murine genes with genetic variants that contribute to hearing loss. A set of 14 high impact NOD/LtJ-specific SNP alleles were identified as described above. Chain-of-Thought (CoT) and self-consistency prompting strategies were used to ask Med-PaLM 2 to output the top 5 most likely genes that could be associated with hearing loss. CoT involves augmenting a one-shot example in a prompt with a step-by-step explanation of how to move towards a final answer [22]. CoT prompts were crafted to provide clear demonstrations on how to appropriately answer the gene-phenotype association questions shown in Supplemental Data File 1. Since the problem was formulated as a multiple-choice question, a self-consistency strategy was used to determine the most consistent answer [23]. Specifically, 100 decoding outputs from the model were sampled by randomizing the input order of the candidate genes. At each inference time, Med-PaLM 2 was instructed to select the top 5 genes associated with hearing loss. The total count for the number of times that each gene was selected across 100 decodes was aggregated.

*Immunoblotting*. Brain tissues obtained from a 5-week-old male A/J, CBA/J or NOD/LtJ mice were homogenized in RIPA buffer supplemented with a protease inhibitor cocktail (Sigma P8340, 1 to 100) using a Precellys tissue homogenizer. Thirty micrograms each of protein lysate were resolved on a 4-15% gradient SDS-polyacrylamide gel and transferred to a nitrocellulose membrane. The membranes were incubated with a 1:1000 dilution of mouse monoclonal anti-µ-crystallin antibody (F-11) (Santa Cruz Biotechnology) or with a 1:4000 dilution of a mouse monoclonal anti-α-tubulin antibody (clone B 5-1-2) (Sigma Aldrich). The anti-Crym antibody is specific for an epitope that is located between amino acids 45 and 85 of the Crym protein. The membranes were then incubated with IRDye® 800CW goat anti-mouse IgG (Licor Bioscience) as the labeled secondary antibody and were scanned using a Licor Odyssey imaging system.

*Generation of KI mice.* CRISPR genome engineering was used to produce KI mice on a NOD genetic background with a reversion of the 2-bp frameshift deletion to wild type (WT) (NOD *Crym*^WT/-^ KI mice) using methods that were described in [24]. To do this, NOD females were super-ovulated by intraperitoneal injection of pregnant mare's serum gonadotropin (PMSG) and human chorionic gonadotropin (hCG). They were paired with NOD males to generate fertilized embryos and pronucleus stage embryos were collected. CRISPR/Cas9, the crRNA, ssODN and PCR primers were obtained from Integrated DNA Technologies (IDT). The crRNA region sequence of the *Crym* sgRNA (Crym-g1): ATTTGGTGAATGGGTAAAGC

The ssODN for Crym KI (that induced the reversion):

GTCATCATCACAGTCACCATGGCAACAGAGCCCATTTTATTTGGTGAATGGGTAAAGCC**A**GGGGCTCACATCAATGGTAAGTCATGCTCCCAGACTGGATCCAGGCTCTA

*Crym* PCR primers are: ACCTTCTCTGTAATCATTAAGCCATTG (Crym-F) and CATCTCCCTGCCTATACTTCTTGAG (Crym-R). HpaII was obtained from New England Biolabs and GoTaq G2 master mix for PCR was obtained from Promega.

CRISPR/Cas9, an sgRNA (Crym-g1) and a single-stranded donor oligonucleotide (ssODN) were electroporated into embryos. Healthy embryos were then transferred into the oviducts of pseudo-pregnant recipient females. The sgRNAs were designed to revert the 2-bp deletion (GG) in NOD with a 2-bp insert (AG), which restored the reading frame. A silent G to A mutation was introduced to prevent re-cutting by inactivating the PAM site of the guide RNA of the KI allele by gRNA. This mutation removed an HpaII restriction site, which was used for genotyping of the pups. The resulting pups were screened by PCR amplification of the targeted region (using the GoTaq G2 master mix for PCR obtained from Promega) followed by HpaII restriction endonuclease digestion (New England Biolabs). The presence of the HpaII-resistant 685 bp PCR amplicon indicates that the desired editing occurred. The 412 and 271 bp bands are from the PCR product of the un-reverted NOD *Crym* allele. The 685 bp amplicon was gel-purified and sequenced to confirm that AG was inserted into the region with the deletion.

*Cochlea immunohistochemistry.* Cochleae samples were prepared as described previously [25]. Cochleae were sectioned at 10 μm on a cryostat (CM3050S, Leica Instruments), mounted on glass slides and stored at −20◦C. Tissue sections were washed with PBS three times, blocked with 5% goat serum and 0.3% Triton X-100 (TX-100) in PBS for 1 h at room temperature, and incubated overnight at 4◦C with the following primary antibodies diluted in 1% goat serum and 0.1% TX-100: a 1:200 dilution of a rabbit polyclonal anti-µ-crystallin antibody (Proteintech) or with a 1:250 dilution of a chicken polyclonal anti-βⅢ tubulin antibody (Sigma-Aldrich). Sections were then incubated with species-appropriate secondary antibodies (Alexa Fluor 488 goat anti-chicken at a 1:500 dilution, Alexa Fluor 555 goat anti-rabbit at a 1:500 dilution, Alexa Fluor 647 labeled phalloidin at a 1:500 dilution) and DAPI at a 1:1000 dilution (Thermo Fisher Scientific) for 1 h at room temperature. Stained tissues were mounted in Prolong anti-fade (Thermo Fisher Scientific) with a coverslip and observed using a LSM880 confocal microscope (Carl Zeiss).

*Human Subjects***.** The study of the 20 patients with hearing loss was approved by the National Taiwan University Hospital Research Ethics Committee (202410065RINC), and informed consents were obtained from all participants and/or their legal guardians. The work on the six patients with genetic diseases was performed through the Undiagnosed Diseases Network (UDN) and was conducted with approval from the National Human Genome Research Institute’s central institutional review board (Registration number 00000014). Informed consent from the subjects were obtained under protocol 15-HG-0130. Any HIPPA related information (gender, age) was randomly altered in the data files. The LLM analyses were performed according to a protocol approved by the Stanford University School of Medicine (protocol #75996 Large Language Model Based Genetic Diagnosis and Discovery). To ensure patient privacy for their genomic sequence data, for each patient, a list with the names of the human genes, which had VUS of interest, was transferred for analysis of their relationship to the patients’ disease by the LLM. For some analyses, the CADD-PHRED score for the VUS was also made available to the LLM. However, the VUS themselves were not transferred for LLM analysis. The researchers participating in the LLM analysis did not receive any protected information about the patient (birthday, patient number, etc.), and when patient data was analyzed by the research team, each subject was referred to by a number based upon the order of entry into the study.

*Human DNA Sequencing and analysis.* The whole exome sequence data from Taiwan patients was generated using pair-end read approaches (125bp x 2 or 150bp x2) with the Illumina platform and kits, and was performed at two sites: the Genetic Resources Core Facility, Johns Hopkins University School of Medicine (TW-WES-01~05; TW-WES-10~20), and Core Instrument Center, National Health Research Institutes (TW-WES-06~09). For the genetic disease patients, exome sequencing was performed at Baylor Genetics, using previously reported methods [26]. Libraries were prepared using a paired-end pre-capture procedure with Illumina multiplexing PE adapters. Sequencing was performed using the Illumina HiSeq sequencing platform for 100 bp paired end reads. FASTQ data were aligned to the human reference genome build GRCh37 using Illumina CASAVA 1.8 software. Variant calling was performed using the Atlas-SNP and Atlas-indel systems developed in-house by Baylor College of Medicine Human Genome Sequencing Center.

A list of potential disease-causing genes was generated using Exomiser, which annotates and ranks variants from exome or genome data based on predicted pathogenicity, population frequency, mode of inheritance, and phenotype matching with known disease genes in humans and model organisms *29, 30*). The complete Exomiser analysis configuration is described in Supplemental Data File 11. Variant prioritization was performed using Exomiser (v14.1.0) with the GRCh38 (hg38) reference genome. Patient phenotypes were encoded using Human Phenotype Ontology (HPO) terms HP:0000365, HP:0008527 for deafness for the Taiwanese patient cohort as a representative example. As family structure information was unavailable for these cases, no PED file was provided; therefore, Exomiser analyses were conducted using VCF files alone, following standard single-sample analysis settings. Multiple modes of inheritance were evaluated, including autosomal dominant, autosomal recessive (homozygous and compound heterozygous), X-linked dominant and recessive, and mitochondrial inheritance. Maximum minor allele frequency thresholds ranged from 0.1% to 2.0%, depending on the inheritance model. Population allele frequencies were obtained from UK10K and gnomAD (exomes and genomes), including African, American, East Asian, non-Finnish European, and South Asian populations. Pathogenicity prediction incorporated REVEL, MVP, CADD, and REMM scores. Variants were filtered to remove non-coding, UTR, intronic, intergenic, upstream/downstream, and regulatory region variants. Variants with a maximum allele frequency above 2.0% were excluded. Gene prioritization was performed using OMIM-based prioritization in combination with the hiPHIVE algorithm. Results were reported without limiting the number of ranked genes. Variant calling for copy number analysis was performed using the Illumina Dragon genome wide depth based CNV caller with custom modifications from Baylor Genetics. Structural variant (SV) calling was performed using the Illumina Manta Structural Variant Caller.

The Exomiser analysis template is below

# These are all the possible options for running Exomiser. Use this as a template for

# your own set-up.

---

analysis:

# hg19 or hg38 - ensure that the application has been configured to run the specified assembly otherwise it will halt.

genomeAssembly: hg38

vcf: /home/lwd97/exomiser-14.1.0/TW-WES-25-88/TW-WES-25_P_LP_VUS.vtable.vcf

ped:

proband:

hpoIds: ['HP:0000365', 'HP:0008527']

# These are the default settings, with values representing the maximum minor allele frequency in percent (%) permitted for an

# allele to be considered as a causative candidate under that mode of inheritance.

# If you just want to analyze a sample under a single inheritance mode, delete/comment-out the others. For AUTOSOMAL_RECESSIVE

# or X_RECESSIVE ensure *both* relevant HOM_ALT and COMP_HET modes are present.

# In cases where you do not want any cut-offs applied an empty map should be used e.g. inheritanceModes: {}

inheritanceModes: {

AUTOSOMAL_DOMINANT: 0.1,

AUTOSOMAL_RECESSIVE_HOM_ALT: 0.1,

AUTOSOMAL_RECESSIVE_COMP_HET: 2.0,

X_DOMINANT: 0.1,

X_RECESSIVE_HOM_ALT: 0.1,

X_RECESSIVE_COMP_HET: 2.0,

MITOCHONDRIAL: 0.2

}

#FULL or PASS_ONLY

analysisMode: PASS_ONLY

# Possible frequencySources:

# UK10K - http://www.uk10k.org/ (UK10K)

# gnomAD - http://gnomad.broadinstitute.org/ (GNOMAD_E, GNOMAD_G)

# note that as of gnomAD v2.1 1000 genomes, ExAC are part of gnomAD

# as of gnomAD v4 TOPMed & ESP are also included in gnomAD

frequencySources: [

UK10K,

GNOMAD_E_AFR,

GNOMAD_E_AMR,

# GNOMAD_E_ASJ,

GNOMAD_E_EAS,

# GNOMAD_E_FIN,

GNOMAD_E_NFE,

# GNOMAD_E_OTH,

GNOMAD_E_SAS,

GNOMAD_G_AFR,

GNOMAD_G_AMR,

# GNOMAD_G_ASJ,

GNOMAD_G_EAS,

# GNOMAD_G_FIN,

GNOMAD_G_NFE,

# GNOMAD_G_OTH,

GNOMAD_G_SAS

]

# Possible pathogenicitySources: (POLYPHEN, MUTATION_TASTER, SIFT), (REVEL, MVP), CADD, REMM, SPLICE_AI, ALPHA_MISSENSE

# REMM is trained on non-coding regulatory regions

# *WARNING* if you enable CADD or REMM ensure that you have downloaded and installed the CADD/REMM tabix files

# and updated their location in the application.properties. Exomiser will not run without this.

pathogenicitySources: [ REVEL, MVP, CADD, REMM ]

# this is the standard Exomiser order.

# all steps are optional

steps: [

#intervalFilter: {interval: 'chr10:123256200-123256300'},

# or for multiple intervals:

#intervalFilter: {intervals: ['chr10:123256200-123256300', 'chr10:123256290-123256350']},

# or using a BED file - NOTE this should be 0-based, Exomiser otherwise uses 1-based coordinates in line with VCF

#intervalFilter: {bed: /full/path/to/bed_file.bed},

#genePanelFilter: {geneSymbols: ['FGFR1','FGFR2']},

# geneBlacklistFilter: { },

failedVariantFilter: { },

#qualityFilter: {minQuality: 50.0},

variantEffectFilter: {

remove: [

FIVE_PRIME_UTR_EXON_VARIANT,

FIVE_PRIME_UTR_INTRON_VARIANT,

THREE_PRIME_UTR_EXON_VARIANT,

THREE_PRIME_UTR_INTRON_VARIANT,

NON_CODING_TRANSCRIPT_EXON_VARIANT,

NON_CODING_TRANSCRIPT_INTRON_VARIANT,

CODING_TRANSCRIPT_INTRON_VARIANT,

UPSTREAM_GENE_VARIANT,

DOWNSTREAM_GENE_VARIANT,

INTERGENIC_VARIANT,

REGULATORY_REGION_VARIANT

]

},

#knownVariantFilter: {}, #removes variants represented in the database

frequencyFilter: {maxFrequency: 2.0},

pathogenicityFilter: {keepNonPathogenic: true},

#inheritanceFilter and omimPrioritiser should always run AFTER all other filters have completed

#they will analyse genes according to the specified modeOfInheritance above- UNDEFINED will not be analysed.

inheritanceFilter: {},

#omimPrioritiser isn't mandatory.

omimPrioritiser: {},

#priorityScoreFilter: {minPriorityScore: 0.4},

#Other prioritisers: Only combine omimPrioritiser with one of these.

#Don't include any if you only want to filter the variants.

hiPhivePrioritiser: {},

# or run hiPhive in benchmarking mode:

#hiPhivePrioritiser: {runParams: 'mouse'},

#phivePrioritiser: {}

#phenixPrioritiser: {}

#exomeWalkerPrioritiser: {seedGeneIds: [11111, 22222, 33333]}

]

outputOptions:

outputContributingVariantsOnly: false

#numGenes options: 0 = all or specify a limit e.g. 500 for the first 500 results

numGenes: 0

# Path to the desired output directory. Will default to the 'results' subdirectory of the exomiser install directory

#outputDirectory: results

#Filename for the output files. Will default to {input-vcf-filename}-exomiser

outputFileName: TW-WES-25

#out-format options: HTML, JSON, TSV_GENE, TSV_VARIANT, VCF (default: HTML)

outputFormats: [TSV_VARIANT]

*Grounding pipeline for Genetic Diagnosis with Gemini*. For the genetic diagnosis task, Gemini was provided with a patient's clinical presentation, along with a list of selected candidate genes. Gemini ranks the candidate genes that are most likely to contain the causative genetic variant for the input clinical features. To enhance the grounding and explainability of Gemini’s genetic diagnoses, the pipeline generates multiple hypotheses explaining the potential causal relationships between each gene within the candidate gene list and the patient’s clinical features. Subsequently, for each hypothesis, a set of relevant abstracts was retrieved from the PubMed database using semantic retrieval with a gecko model [27]. These abstracts, in conjunction with the patient's conditions and the hypothesis itself, were then evaluated by the model. The evaluation involved generating a list of abstract titles that either support or oppose the hypothesis, followed by a detailed explanation of how abstract content aligned with or contradicted the proposed hypothesis.  Finally, a score ranging from 1 (strong opposition) to 5 (strong support) was assigned to each hypothesis based on the evidence presented in the retrieved abstracts.  The average of these scores across all hypotheses for a given gene constituted its final gene score. By ranking the gene scores, the pipeline prioritized the genes that most likely had causative mutations, which streamlines the diagnostic process. For this study, we employed five hypotheses per gene and retrieved 20 abstracts per hypothesis for scoring. These parameters were not subjected to further optimization. Abstract retrieval was facilitated by embedding the 27 million abstracts in our internal PubMed repository. The prompts for hypothesis generation and grounding are shown below.

**Prompt for hypothesis generation**

"""

=== Patient Information ===

Patient Case Summary: {CASE_SUMMARY}

List of Human Phenotype Ontology Terms: {HP_TERMS}

=== Gene ===

{GENE}

=== Web Search Results ===

{search_text}

=== Instructions ===

You are a helpful genetic assistant. You need to identify genes with causal mutations for these abnormalities for a patient.

You are provided with a summary of all the phenotypic abnormalities of the patient, along with a single gene that may have a mutation that might cause the abnormalities.

You are also given access to web search results related to the gene.

Your task is to generate {TOP_K} hypotheses for why the gene is causal to the patient's case.

Make sure each hypothesis is thorough, providing detailed justifications for why the gene is causal to each relevant phenotypic abnormality.

Based on the current patient information and the gene, find the top {TOP_K} possible hypotheses by filling in the following template:

{template}

"""

**Prompt for grounding**

"""

=== Patient Information ===

Patient Case Summary: {CASE_SUMMARY}

List of Human Phenotype Ontology Terms: {HP_TERMS}

=== Abstracts ===

{abstracts}

=== Task ===

You are a helpful genetic assistant analyzing a hypothesis.

You are given a summary of all the phenotypic abnormalities of a patient.

Your task is to analyze a hypothesis about why a certain gene is causal to the patient's case.

You are also given the most relevant research articles related to the given hypothesis.

=== Hypothesis ===

{hypothesis}

=== Instructions ===

When analyzing the hypothesis, follow the following instructions:

* First write a short list of up to 6 abstract titles which would either support or oppose the hypothesis. For each include your reasoning for why this abstract is relevant for the hypothesis.

* Second, write detailed aspects of how the abstracts either support or oppose the hypothesis. Try very hard to find both supporting and opposing aspects.

* Lastly, score the hypothesis based on the supporting and opposing aspects from the abstracts using a scale from 1 to 10, where a score of 1 means that the abstracts oppose the hypothesis, and a score of 10 means that the abstracts strongly support the hypothesis.

=== Evaluation of hypothesis ===

Evaluate the hypothesis by filling in the following template:

{grounding_template}

"""

Clinical confirmation of the *IRAK4* SNP and of the SV was performed at Baylor Genetics by Sanger sequencing and using a custom assay for the breakpoint junction. A long-range PCR assay was used to amplify the breakpoint junction locus and the locus for the c.364C>T SNP. These variants were also determined to be in trans by this methodology.

**Supplemental References**

[1] P. Kumar, S. Henikoff, P. C. Ng, Predicting the effects of coding non-synonymous variants on protein function using the SIFT algorithm. *Nat Protoc* **2009**, *4* (7), 1073, <https://doi.org/10.1038/nprot.2009.86>.

[2] I. Adzhubei, D. M. Jordan, S. R. Sunyaev, Predicting functional effect of human missense mutations using PolyPhen-2. *Curr Protoc Hum Genet* **2013**, *Chapter 7*, Unit7 20, <https://doi.org/10.1002/0471142905.hg0720s76>.

[3] G. M. Cooper, E. A. Stone, G. Asimenos, N. C. S. Program, E. D. Green, S. Batzoglou, A. Sidow, Distribution and intensity of constraint in mammalian genomic sequence. *Genome Res* **2005**, *15* (7), 901, <https://doi.org/10.1101/gr.3577405>.

[4] a) A. Niroula, M. Vihinen, How good are pathogenicity predictors in detecting benign variants? *PLoS Comput Biol* **2019**, *15* (2), e1006481, <https://doi.org/10.1371/journal.pcbi.1006481>; b) Y. Choi, G. E. Sims, S. Murphy, J. R. Miller, A. P. Chan, Predicting the functional effect of amino acid substitutions and indels. *PLoS One* **2012**, *7* (10), e46688, <https://doi.org/10.1371/journal.pone.0046688>.

[5] J. S. Amberger, C. A. Bocchini, A. F. Scott, A. Hamosh, OMIM.org: leveraging knowledge across phenotype-gene relationships. *Nucleic Acids Res* **2019**, *47* (D1), D1038, <https://doi.org/10.1093/nar/gky1151>.

[6] C. UniProt, UniProt: the Universal Protein Knowledgebase in 2023. *Nucleic Acids Res* **2023**, *51* (D1), D523, <https://doi.org/10.1093/nar/gkac1052>.

[7] M. J. Landrum, J. M. Lee, M. Benson, G. Brown, C. Chao, S. Chitipiralla, B. Gu, J. Hart, D. Hoffman, J. Hoover, W. Jang, K. Katz, M. Ovetsky, G. Riley, A. Sethi, R. Tully, R. Villamarin-Salomon, W. Rubinstein, D. R. Maglott, ClinVar: public archive of interpretations of clinically relevant variants. *Nucleic Acids Res* **2016**, *44* (D1), D862, <https://doi.org/10.1093/nar/gkv1222>.

[8] M. J. Landrum, S. Chitipiralla, G. R. Brown, C. Chen, B. Gu, J. Hart, D. Hoffman, W. Jang, K. Kaur, C. Liu, V. Lyoshin, Z. Maddipatla, R. Maiti, J. Mitchell, N. O'Leary, G. R. Riley, W. Shi, G. Zhou, V. Schneider, D. Maglott, J. B. Holmes, B. L. Kattman, ClinVar: improvements to accessing data. *Nucleic Acids Res* **2020**, *48* (D1), D835, <https://doi.org/10.1093/nar/gkz972>.

[9] S. Richards, N. Aziz, S. Bale, D. Bick, S. Das, J. Gastier-Foster, W. W. Grody, M. Hegde, E. Lyon, E. Spector, K. Voelkerding, H. L. Rehm, A. L. Q. A. Committee, Standards and guidelines for the interpretation of sequence variants: a joint consensus recommendation of the American College of Medical Genetics and Genomics and the Association for Molecular Pathology. *Genet Med* **2015**, *17* (5), 405, <https://doi.org/10.1038/gim.2015.30>.

[10] S. E. Hunt, B. Moore, R. M. Amode, I. M. Armean, D. Lemos, A. Mushtaq, A. Parton, H. Schuilenburg, M. Szpak, A. Thormann, E. Perry, S. J. Trevanion, P. Flicek, A. D. Yates, F. Cunningham, Annotating and prioritizing genomic variants using the Ensembl Variant Effect Predictor-A tutorial. *Hum Mutat* **2022**, *43* (8), 986, <https://doi.org/10.1002/humu.24298>.

[11] P. Rentzsch, D. Witten, G. M. Cooper, J. Shendure, M. Kircher, CADD: predicting the deleteriousness of variants throughout the human genome. *Nucleic Acids Res* **2019**, *47* (D1), D886, <https://doi.org/10.1093/nar/gky1016>.

[12] M. Kircher, D. M. Witten, P. Jain, B. J. O'Roak, G. M. Cooper, J. Shendure, A general framework for estimating the relative pathogenicity of human genetic variants. *Nat Genet* **2014**, *46* (3), 310, <https://doi.org/10.1038/ng.2892>.

[13] J. Abramson, J. Adler, J. Dunger, R. Evans, T. Green, A. Pritzel, O. Ronneberger, L. Willmore, A. J. Ballard, J. Bambrick, S. W. Bodenstein, D. A. Evans, C. C. Hung, M. O'Neill, D. Reiman, K. Tunyasuvunakool, Z. Wu, A. Zemgulyte, E. Arvaniti, C. Beattie, O. Bertolli, A. Bridgland, A. Cherepanov, M. Congreve, A. I. Cowen-Rivers, A. Cowie, M. Figurnov, F. B. Fuchs, H. Gladman, R. Jain, Y. A. Khan, C. M. R. Low, K. Perlin, A. Potapenko, P. Savy, S. Singh, A. Stecula, A. Thillaisundaram, C. Tong, S. Yakneen, E. D. Zhong, M. Zielinski, A. Zidek, V. Bapst, P. Kohli, M. Jaderberg, D. Hassabis, J. M. Jumper, Accurate structure prediction of biomolecular interactions with AlphaFold 3. *Nature* **2024**, *630* (8016), 493, <https://doi.org/10.1038/s41586-024-07487-w>.

[14] C. Kilkenny, W. J. Browne, I. C. Cuthill, M. Emerson, D. G. Altman, Improving bioscience research reporting: the ARRIVE guidelines for reporting animal research. *PLoS Biol* **2010**, *8* (6), e1000412, <https://doi.org/10.1371/journal.pbio.1000412>.

[15] a) R. Seist, L. D. Landegger, N. G. Robertson, S. Vasilijic, C. C. Morton, K. M. Stankovic, Cochlin Deficiency Protects Against Noise-Induced Hearing Loss. *Front Mol Neurosci* **2021**, *14*, 670013, <https://doi.org/10.3389/fnmol.2021.670013>; b) S. Early, M. A. Saad, S. Mallidi, A. Mansour, R. Seist, T. Hasan, K. M. Stankovic, A fluorescent photoimmunoconjugate for imaging of cholesteatoma. *Sci Rep* **2022**, *12* (1), 19905, <https://doi.org/10.1038/s41598-022-22072-9>.

[16] J. Chen, L. D. Landegger, Y. Sun, J. Ren, N. Maimon, L. Wu, M. R. Ng, J. W. Chen, N. Zhang, Y. Zhao, X. Gao, T. Fujita, S. Roberge, P. Huang, R. K. Jain, S. R. Plotkin, K. M. Stankovic, L. Xu, A cerebellopontine angle mouse model for the investigation of tumor biology, hearing, and neurological function in NF2-related vestibular schwannoma. *Nat Protoc* **2019**, *14* (2), 541, <https://doi.org/10.1038/s41596-018-0105-7>.

[17] Z. Fang, G. Peltz, An Automated Multi-Modal Graph-Based Pipeline for Mouse Genetic Discovery. *Bioinformatics* **2022**, *38* (13), 3385, <https://doi.org/10.1093/bioinformatics/btac356>.

[18] H. Li, R. Durbin, Fast and accurate short read alignment with Burrows-Wheeler transform. *Bioinformatics* **2009**, *25* (14), 1754, <https://doi.org/btp324> [pii]

10.1093/bioinformatics/btp324.

[19] H. Li, B. Handsaker, A. Wysoker, T. Fennell, J. Ruan, N. Homer, G. Marth, G. Abecasis, R. Durbin, S. Genome Project Data Processing, The Sequence Alignment/Map format and SAMtools. *Bioinformatics* **2009**, *25* (16), 2078, <https://doi.org/10.1093/bioinformatics/btp352>.

[20] W. McLaren, L. Gil, S. E. Hunt, H. S. Riat, G. R. Ritchie, A. Thormann, P. Flicek, F. Cunningham, The Ensembl Variant Effect Predictor. *Genome Biol* **2016**, *17* (1), 122, <https://doi.org/10.1186/s13059-016-0974-4>.

[21] Q. Y. Zheng, K. R. Johnson, L. C. Erway, Assessment of hearing in 80 inbred strains of mice by ABR threshold analyses. *Hear Res* **1999**, *130* (1-2), 94, <https://doi.org/10.1016/s0378-5955(99)00003-9>.

[22] J. Wei, X. Wang, D. Schuurmans, M. Bosma, F. Xia, E. Chi, Q. V. Le, D. Zhou, Chain-of-thought prompting elicits reasoning in large language models. *Advances in Neural Information Processing Systems* **2022**, *35*, 24824.

[23] X. Wang, J. Wei, D. Schuurmans, Q. Le, E. Chi, S. Narang, A. Chowdhery, D. Zhou, Self-consistency improves chain of thought reasoning in language models. *arXiv preprinnt* **2022**, *arXiv:2203.11171*.

[24] J. R. Bagley, Y. Tan, W. Zhu, Z. Cheng, S. Takeda, Z. Fang, A. Arslan, M. Wang, Y. Guan, L. Jiang, R. Jian, F. Gu, I. Parada, D. Prince, J. D. Jentsch, G. Peltz, Neuron Navigator 1 (Nav1) regulates the response to cocaine in mice. *Commun Biol* **2023**, *6* (1), 1053, <https://doi.org/10.1038/s42003-023-05430-9>.

[25] R. Seist, M. Tong, L. D. Landegger, S. Vasilijic, H. Hyakusoku, S. Katsumi, C. E. McKenna, A. S. B. Edge, K. M. Stankovic, Regeneration of Cochlear Synapses by Systemic Administration of a Bisphosphonate. *Front Mol Neurosci* **2020**, *13*, 87, <https://doi.org/10.3389/fnmol.2020.00087>.

[26] K. Splinter, D. R. Adams, C. A. Bacino, H. J. Bellen, J. A. Bernstein, A. M. Cheatle-Jarvela, C. M. Eng, C. Esteves, W. A. Gahl, R. Hamid, H. J. Jacob, B. Kikani, D. M. Koeller, I. S. Kohane, B. H. Lee, J. Loscalzo, X. Luo, A. T. McCray, T. O. Metz, J. J. Mulvihill, S. F. Nelson, C. G. S. Palmer, J. A. Phillips, 3rd, L. Pick, J. H. Postlethwait, C. Reuter, V. Shashi, D. A. Sweetser, C. J. Tifft, N. M. Walley, M. F. Wangler, M. Westerfield, M. T. Wheeler, A. L. Wise, E. A. Worthey, S. Yamamoto, E. A. Ashley, N. Undiagnosed Diseases, Effect of Genetic Diagnosis on Patients with Previously Undiagnosed Disease. *N Engl J Med* **2018**, *379* (22), 2131, <https://doi.org/10.1056/NEJMoa1714458>.

[27] J. Lee, Z. Dai, X. Ren, b. Chen, D. Cer, J. R. Cole, K. Hui, M. Boratko, D. Kapadia, W., Y. Luan, S. M. Karthik Duddu, G. H. Abrego, W. Shi, N. Gupta, A. Kusupati, P. Jain, S. R. Jonnalagadda, M. Chang, I. Naim, Gecko: Versatile Text Embeddings Distilled from Large Language Models. *ArXiv* **2024**, *arXiv:2403.20327v1*, <https://doi.org/https://doi.org/10.48550/arXiv.2403.20327>.

[28] R. E. Sorge, T. Trang, R. Dorfman, S. B. Smith, S. Beggs, J. Ritchie, J. S. Austin, D. V. Zaykin, H. V. Meulen, M. Costigan, T. A. Herbert, M. Yarkoni-Abitbul, D. Tichauer, J. Livneh, E. Gershon, M. Zheng, K. Tan, S. L. John, G. D. Slade, J. Jordan, C. J. Woolf, G. Peltz, W. Maixner, L. Diatchenko, Z. Seltzer, M. W. Salter, J. S. Mogil, Genetically determined P2X7 receptor pore formation regulates variability in chronic pain sensitivity. *Nat Med* **2012**, *18* (4), 595, <https://doi.org/10.1038/nm.2710>.

[29] G. Liao, J. Wang, J. Guo, J. Allard, J. Cheng, A. Ng, S. Shafer, A. Puech, J. D. McPherson, D. Foernzler, G. Peltz, J. Usuka, In Silico Genetics: Identification of a Functional Element Regulating H2-Ea Gene Expression. *Science* **2004**, *306* (5696), 690.

[30] Y. Guo, P. Weller, E. Farrell, P. Cheung, B. Fitch, D. Clark, S. Y. Wu, J. Wang, G. Liao, Z. Zhang, J. Allard, J. Cheng, A. Nguyen, S. Jiang, S. Shafer, J. Usuka, M. Masjedizadeh, G. Peltz, In silico pharmacogenetics of warfarin metabolism. *Nat Biotechnol* **2006**, *24* (5), 531, <https://doi.org/10.1038/nbt1195>.

[31] M. Fornerod, M. Ohno, M. Yoshida, I. W. Mattaj, CRM1 is an export receptor for leucine-rich nuclear export signals. *Cell* **1997**, *90* (6), 1051, <https://doi.org/10.1016/s0092-8674(00)80371-2>.

**Table S1**. This table shows the candidate gene sets analyzed by Med-PaLM 2. The murine candidate genes, which had allelic patterns that correlated with the pattern of phenotypic responses of the inbred strains for the indicated phenotype, for each referenced study are listed. The experimentally proven causative genetic factor was correctly identified by the PaLM-Med2 response. The queries and responses are shown in supplemental data file 1.

| **Phenotype** | **Gene Candidates** | **Correct**  **Answer (Ref)** |
| --- | --- | --- |
| Cataract | *Miox, Nid1, Trappc1, Pi4ka, Adgrv1, Slc8b1* | *Nid1* [17] |
| Diabetes | *Krtap5-1, Act1, Act2, Act3, Actb, Abra, Tlr5, Myh1, Myh2, Myh8, Unc45a, Cdc102a* | *Tlr5* [17] |
| Chronic Pain | *P2rx7, Arntl, Pttg1, Kcnk2, Clcn4-2,*  *Mhrt, Mh7b, Cttnb1, Dact, Cdh2* | *P2rx7* [28] |
| Albino Skin | *Grm5, Fzdr, Dlg2, Tyr, Polc4, Sec11a,*  *Ap3b2, Rab38, Tmem135, Cedc81,*  *17rn6, Fcd, Picalm, Cdc83, Nrs155* | *Tyr [29]* |
| Warfarin  Metabolism | *Igsf8, Kcnj9, Kcnj10, Slc30a1, Icnh1, Masp2, Itpr1, Grin2b, Ctsc, Art5, Trpc2, Rrm1, Adam9, Lpl, Mmp2, Nnmt, Acy1,Gabrg2, Ctsl, Adprtl2, Abhd4, Mmp14, Psmb5, cacna1i, Tmprss2,Sncaip, Aldh7a1, Cyp2c55, Abcc2, Gabre* | *Cyp2c55* [30] |
| Aromatic  Hydrocarbon  Response | *Ahr, Dnpep, Dusp2, Vdr, Ren1,*  *Cyp2b19, Pola1, Esr2* | *Ahr*  [29] |

**Table S2.** The *Cdh23^753(A/G)^* alleles present in 54 inbred strains are shown. Inbred strains with early onset hearing loss are in bold, and virtually all have the *Cdh23^753A^*; but many strains with *Cdh23^753A^* alleles do not develop early onset hearing loss. I/LnJ mice may have been incorrectly labeled as having an early onset hearing loss in [21]. Examination of I/LnJ data [21] revealed that they were only evaluated at 12 weeks of age, their hearing loss was relatively mild, and there was significant variability in the measured thresholds.

**Table S3**. *Crym* was identified by Med-PaLM 2 as the gene whose high impact SNP alleles were most likely to cause spontaneous hearing loss in NOD/LtJ mice. Fourteen genes with high impact SNP alleles were identified by comparing NOD/LtJ genomic sequence with 10 strains that did not manifest age-related hearing loss. Med-PaLM 2 analyzed the gene list 100 times after the gene order was randomly permutated. The number of times each indicated gene was among the top 5 genes identified by Med-PaLM 2 as associated with hearing loss is shown. *Tmem242* was the 2^nd^ most frequently identified gene, but it was not directly associated with hearing loss. Tmem242 is required for assembly of the mitochondrial ATP synthase complex, and mitochondrial abnormalities (but not *Tmem242* itself) are associated with hearing loss syndrome. The *Med1, Mast4* and *Stk38* associations resulted from false associations (Mast cells, Med center, etc.) with variants of the gene name.

Gene #

***Crym***  98

*Tmem242* 90

*Mast4* 75

*Med1* 57

*Stk38* 53

*Sdc2* 45

*Eppk1* 15

*Spertl* 4

*Fcgr1* 4

*Ifi27l2b* 1

*Stxbp4* 1

Zfp629 1

Il23r 1

Armt1 1

**Table S4.** The causative alleles identified for subjects with hearing loss (**A**) or rare genetic diseases (**B**) that are discussed in this paper are shown using the Human Genome Variation Society (HGVS) nomenclature. Two variants in *POL3A* were identified in Genetic disease patient 6.

**A. Hearing Loss**

**#** **HGVS**

1 *MYH14:ENST00000642316.2:c.2893G>A:p.(Glu965Lys)*

2 *ACTG1:ENST00000573283.7:c.721G>A:p.(Glu241Lys)*

3 *GJB2:ENST00000382848.5:c.428G>A:p.(Arg143Gln)*

4 *TYR:ENST00000263321.6:c.230_232dup:p.(Arg77_Glu78insGly)*

5 *PTPN11:ENST00000351677.7:c.1403C>T:p.(Thr468Met)*

6 *GJB2:ENST00000382848.5:c.235del:p.(Leu79Cysfs*3)*

7 *TECTA:ENST00000392793.6:c.5825A>G:p.(Tyr1942Cys)*

8 *KCNQ4:ENST00000347132.10:c.853G>A:p.(Gly285Ser)*

9 GJB2:ENST00000382848.5:c.571T>C:p.(Phe191Leu)

10 *CEP250:ENST00000397527.6:c.665G>A:p.(Arg222His)*

*CEP250:ENST00000397527.6:c.4154G>A:p.(Arg1385His)*

11 *P2RX2:ENST00000643471.2:c.137G>A:p.(Arg46His)*

12 *GSDME:ENST00000645220.1:c.1454T>G:p.(Leu485Arg)*

13 *COL11A1:ENST00000370096.9:c.5094C>A:p.(Phe1698Leu)*

14 *MYO3A:ENST00000642920.2:c.1104_1105delCGinsTA:p.(Val369Ile)*

15 *MYO7A:ENST00000409709.9:c.4757A>G:p.(Asn1586Ser)*

16 *TMC1:ENST00000297784.10:c.2050G>C:p.(Asp684His)*

17 *POU4F3:ENST00000646991.2:c.281C>T:p.(Thr94Ile)*

18 *CEACAM16:ENST00000587331.7:c.865G>A:p.(Gly289Arg)*

19 Unknown

20 *MYO6:ENST00000369977.8:c.3530G>A:p.(Arg1177His)*

**B. Rare Genetic Disease**

**# HGVS**

1 *IRAK4:ENST00000448290.6:c.364C>T:p.(Gln122*)*

2 *ODC1:ENST00000234111.9:c.1313_1316del:p.(Pro438Argfs*9)*

3 *CDKL5:ENST00000623535.2:c.2828_2829del:p.(Arg943Asnfs*11)*

4 *ADSS1:ENST00000330877.2:c.781G>A:p.(Asp261Asn)*

5 *RHOBTB2:ENST00000251822.6:c.1382G>A:p.(Arg461His)*

6 *POLR3A:ENST00000372371.3:c.1771-7C>G:p.?*

*POLR3A:ENST00000372371.3:c.1400C>T:p.(Ser467Leu)*

**Table S5**. The likely causative genes for hearing loss were identified for 19 of 20 patients analyzed by the biomedical LLM pipeline. The identified causative gene (and variant allele) and age of onset for hearing loss are shown for each patient. Also shown are the clinical experts’ assessment of the genetic candidates (concur, tentative concur, or not agree) and their comments, and subsequent follow up. The clinical experts agreed or tentatively agreed with 18 of the AI pipeline’s findings; and disagreed with the predictions for two patients (10 and 15); but pipeline output was used by the experts to identify an alternative candidate for patient 15. Of importance, concurrence does not imply that a definitive cause was identified by the AI pipeline but indicates that it identified the most likely candidate factor for a patient at that time. As indicated in Table 1, other candidates were subsequently found for patients 13, 14, and 20. Tentative concurrence indicates that additional evaluation of parents (or other data) could be pursued to confirm causality for the candidate or to find other candidates.

**Gene Expert Age of Expert**

**Var Assessment Onset Comment and Follow Up**

1 *MYH14*  Concur 33 Heterozygous at this locus

*Glu965Lys*

2 *ACTG1* Concur 3 Heterozygous at this locus

*Glu241Lys*

3 *GJB2* Concur 0.33 This variant was in the father’s genome

*Arg143Gln* confirming an autosomal dominant inheritance pattern.

4 *TYR* Concur 10 The patient had deafness and albinism. The patient’s father

*Arg422Gln*  had *Arg422GLN* and only deafness. They Identified a 2nd

variant to form a compound heterozygote

*TYR (NM_000372.5): c.230_232dup (p.Arg77_Glu78insGly)*

But are also seeking other genetic factors.

5 *PTPN11* Concur 0.66 Heterozygous at this locus

*Thr468Met*

6 *GJB2* Concur 2 Homozygous for this mutation. This mutation was present

*Leu79CysfsTer3*  as heterozygote in maternal and paternal genomes.

7 *TECTA* Concur 0.5 Identified a 2^nd^ variant to form a compound heterozygote

*Tyr1942Cys TECTA (NM_005422.4): c.5488G>A, p.Val1830Met*

8 *KCNQ4* Concur 5

*Gly285Ser*

9 *GJB2* Concur 4 Identified 2^nd^ variant to form a compound heterozygote

*Phe191Leu GJB2 (NM_004004.6): c.109G>A, p.Val37Ile*

10 *CEP250* Not 0.5 *Arg222His* is a benign variant, so parents

*Arg1385His A*gree should be evaluated to identify a 2^nd^ variant

*Arg222His* May have found another candidate

**Gene Expert Age of Expert**

**Var Assessment Onset Comment and Follow Up**

11 *P2RX2* Concur 0.6 This mutation was de novo, it was not present in either

*Arg46His* paternal or maternal genome.

12 *GSDME* Concur 0.5 Unaffected sibling does not have this allele

*Leu485Arg* Recommended evaluation of unaffected parents.

13 *COL11A1* Tentative 14 Recommended evaluation of parents to further

*Phe1698Leu* Concur assess the mutation and search for other variants.

Subsequent analysis identified other

candidates that are being pursued (*BSND Thr6Ala)*.

14 *MYO3A* Tentative 2 Recommend evaluation of parents to assess mutation

*Val369Ile* Concur Proband’s unaffected mother was found to have this allele.

15 *MYO7A*  Not 1 Evaluated other high ranked genes by LLM and Identified

*Asn1586Ser* Agree EYA4 (DFNA10) variant (*Val437Ile*) as better alternative

16 *TMC1* Concur 24 Can be associated with AD (DFNA36) or AR (DFNB7)

*Asp684His* deafness. It should be viewed as a carried variant.

Subsequent analysis revealed that the un-affected father

does not have this allele. No better variants identified yet.

17 *POU4F3* Tentative 17 Patient had mild to moderate deafness. Felt likely, but other

*Thr94Ile* Concur possible variants should be sought. Recommend evaluation

of parents, but this was not possible.

18 *CEACAM16* Concur 0.8. Felt most likely, but recommend evaluation

*Gly289Arg* of unaffected parents to determine if *de novo*

19 UNKNOWN Concur 0.33 No AD variant identified, but found possible

low impact splicing variant as alternative

20 *MYO6* Concur 0.25 Felt most likely, but recommend evaluation

*Leu1283ValfsTer39* of unaffected parents to assess mutation. The proband’s

unaffected mother was found to have this allele, so other

variants subsequently sought.

**Figure S1.** Cochlear cryostat sections from 10-week-old CBA/J mice that were stained with an anti-CRYM antibody (magenta), DAPI (blue) and phalloidin (yellow). **(A)** Low magnification images show that CRYM is expressed in the spiral ganglion neurons (SGNs), Organ of Corti (OC, rectangles) and lateral wall (LW). **(B)** Magnified images of the OC in both apical and basal turns show that CRYM is expressed in the supporting cells (Deiters’ cells [DCs], outer and inner pillar cells [OPCs and IPCs]), but not outer and inner hair cells (OHCs and IHCs). **(C)** Magnified images of the lateral wall in both apical and basal turns show that CRYM is expressed in the root cells (RC) and cells (arrows) of stria vascularis (SV). Scale bars: **A**, 200μm; **B**, 20μm; **C**, 50μm.


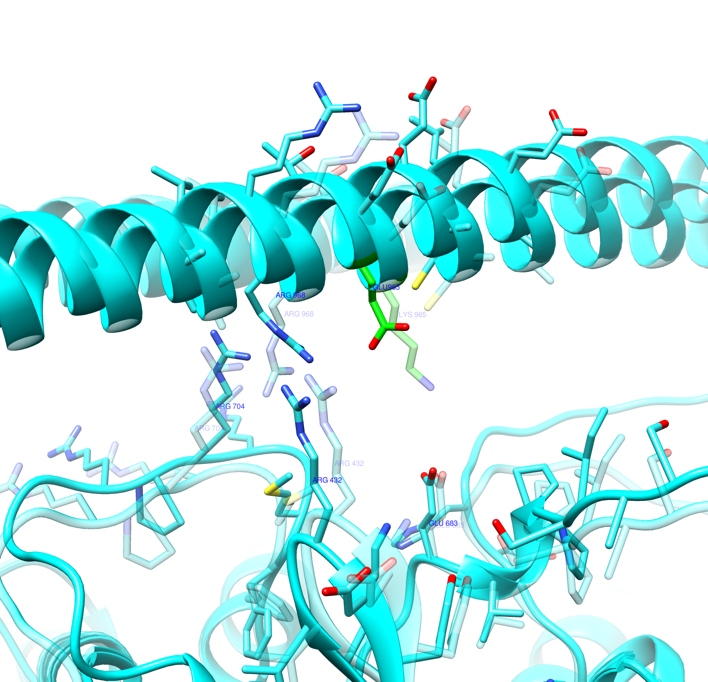

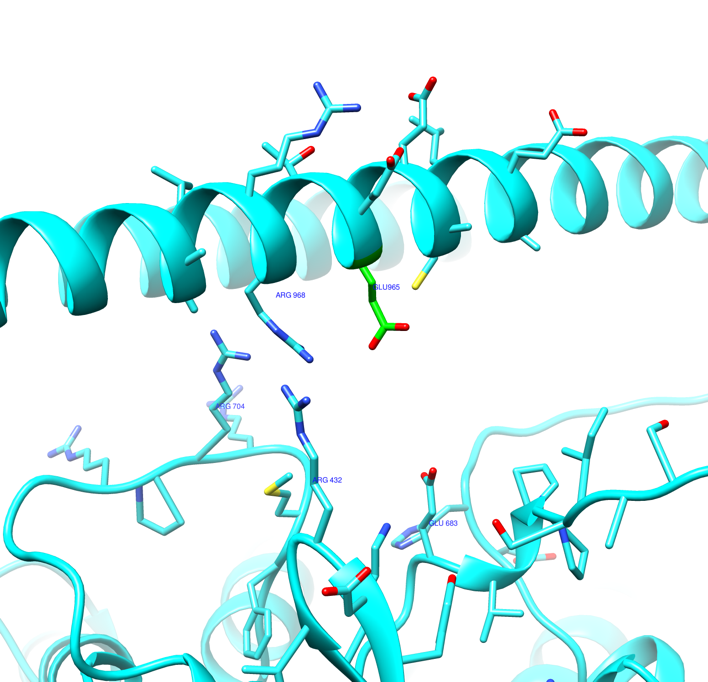

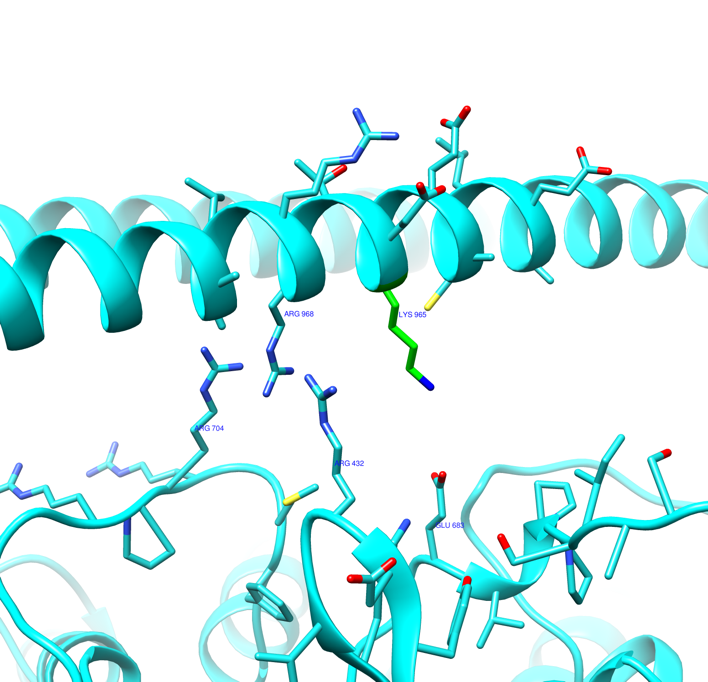


**Figure S2.**The effect of the *Glu965Lys* mutation on MYH14 structure in the region surrounding the mutation site. The Alphafold3 [13] predicted structures of MYH14 with Glu965 (marked in green) (**Top left**) or Lys965 (marked in green) (**Top right**); or a combined figure superimposing the MYH14 Glu965 and MYH14 Lys965 structures, which are shown above the MYH14 motor domain (**Bottom**). The *Glu965Lys* mutation changes the acidic amino acid at position 965 to a basic amino acid. This change could cause the amino acid at position 965 to move away from Arg968 and consequently from Glu683, which could cause the helix containing Glu965 (upper) to move closer to the myosin motor domain (lower). By this mechanism, the *Glu965Lys* mutation would significantly alter protein-protein interactions between the motor domain and the alpha helical region of MYH14.

**
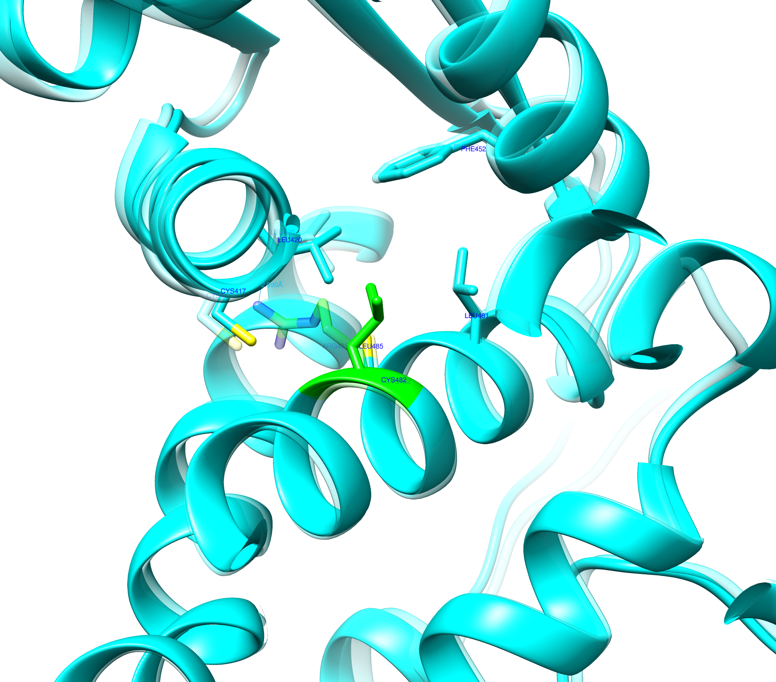
**
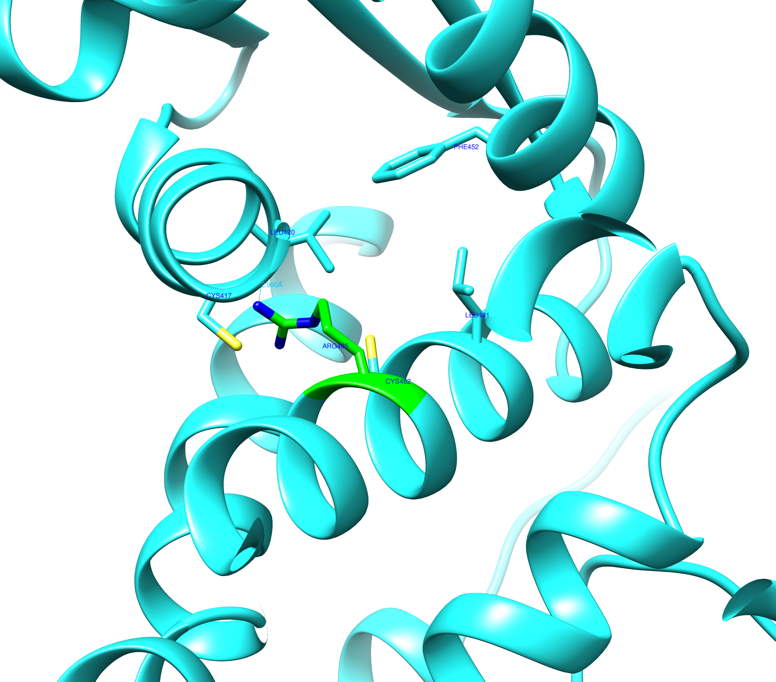

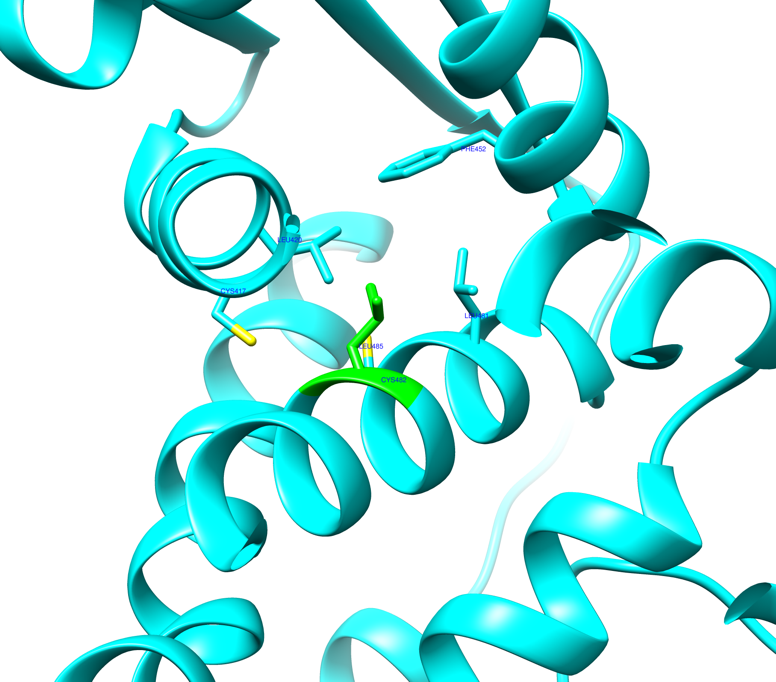


**Figure S3**. The structural effect of the *GSDME Leu485Arg* mutation on the region surrounding the mutation site. The Alphafold3 [13] predicted structures of GSDME with Leu485 (marked in green, Top left) or Arg485 (also marked in green, Top right); or a combined figure superimposing the GSDME Leu485 and Arg485 structures (Bottom). The *Leu485Arg* mutation changes the nonpolar amino acid at position 485 to a polar amino acid, which reduces the stability of the α-helix that it resides in. GSDME Arg485 forms a new hydrogen bond with the oxygen atom on the main chain of Leu416 (marked in blue), which displaces the helix containing Leu416, and this structural change is likely to alter GSDME function.


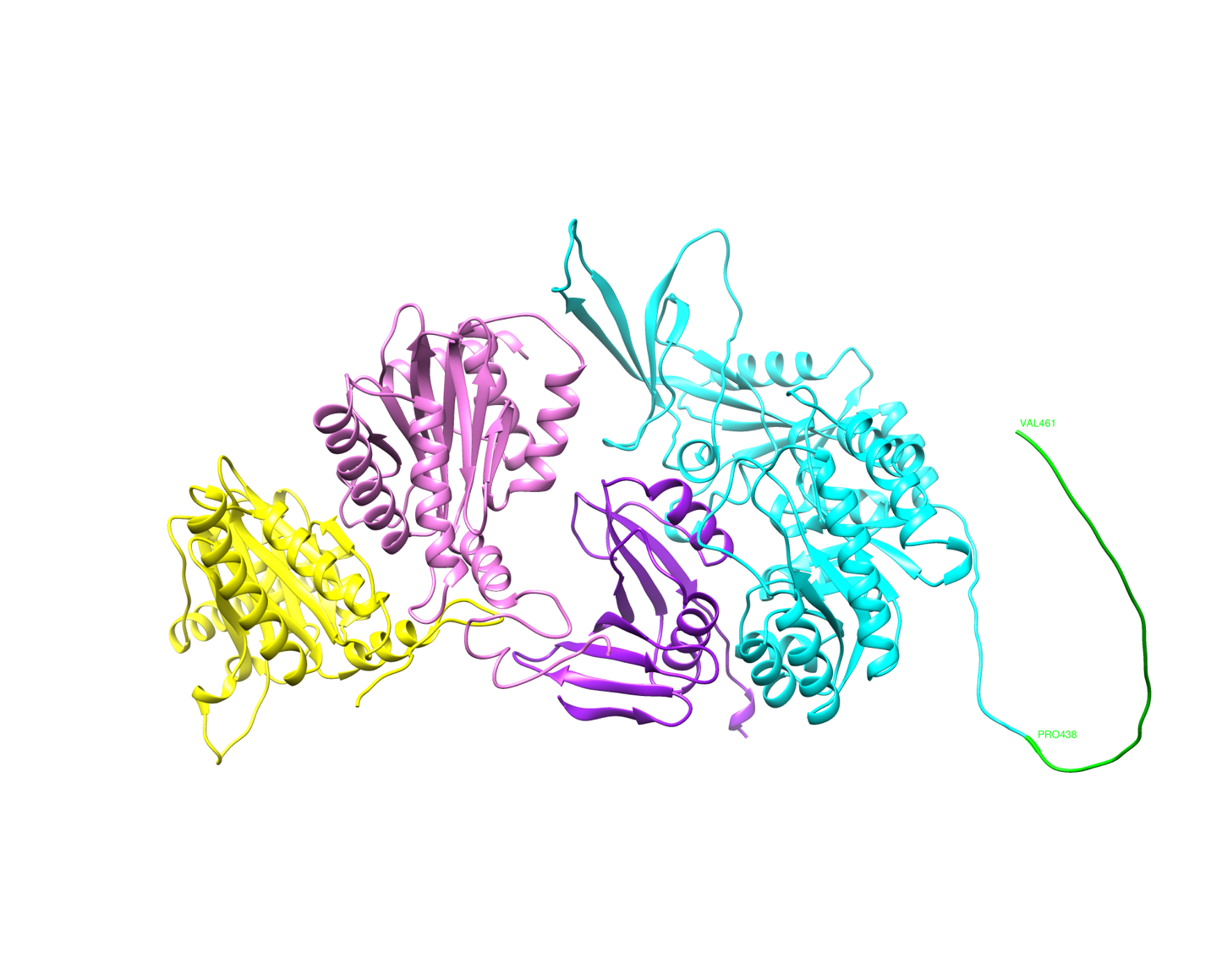


**Figure S4**. The *Pro438Argfs*9* frameshift mutation effects the C-terminal region of the ODC1 protein, which could be required for interaction with Ornithine decarboxylase antizyme 1 (Oaz1) and Proteasome subunits alpha type-6 and type-7 (PSMA6, PSMA7). The Alphafold3 [13] predicted structures of ODC1(blue) and the region affected by the *Pro438Argfs*9* mutation region marked in green; Oaz1 without its unstable N terminal region is shown in purple; and proteasome subunits PSMA6 and PSMA7 are shown in yellow and pink, respectively. Alphafold3 predicts that amino acids 438-461 of ODC1 have a low pIDDT value, which means that their structure can change dynamically. Hence, despite the binding of Oaz1, PSMA6, and PAMA7 to ODC1, the C terminal region of mutated ODC1 may not be able to form a valid structure that can interact with other domains. However, the Pro438Argfs*9 mutation will drastically change the sequence and length of the loop region at the C terminus of ODC1, which may reduce its ability to be recognized and degraded by the proteasome.


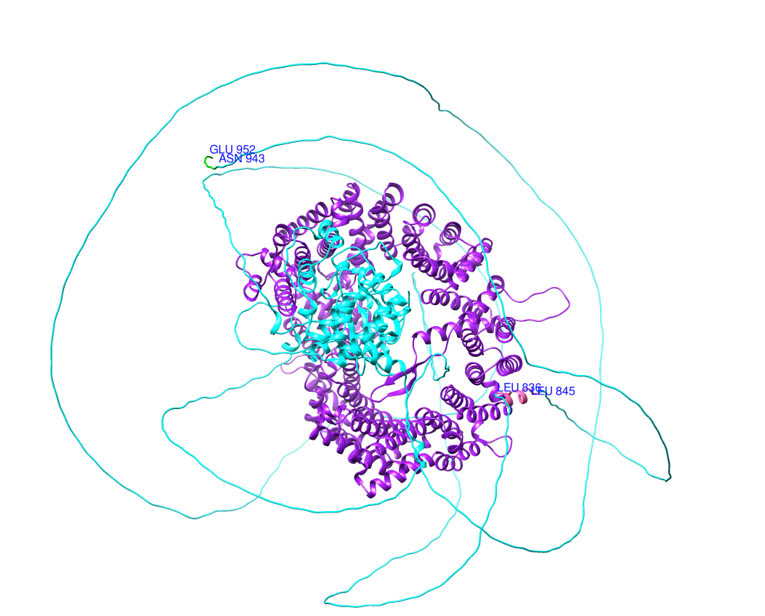

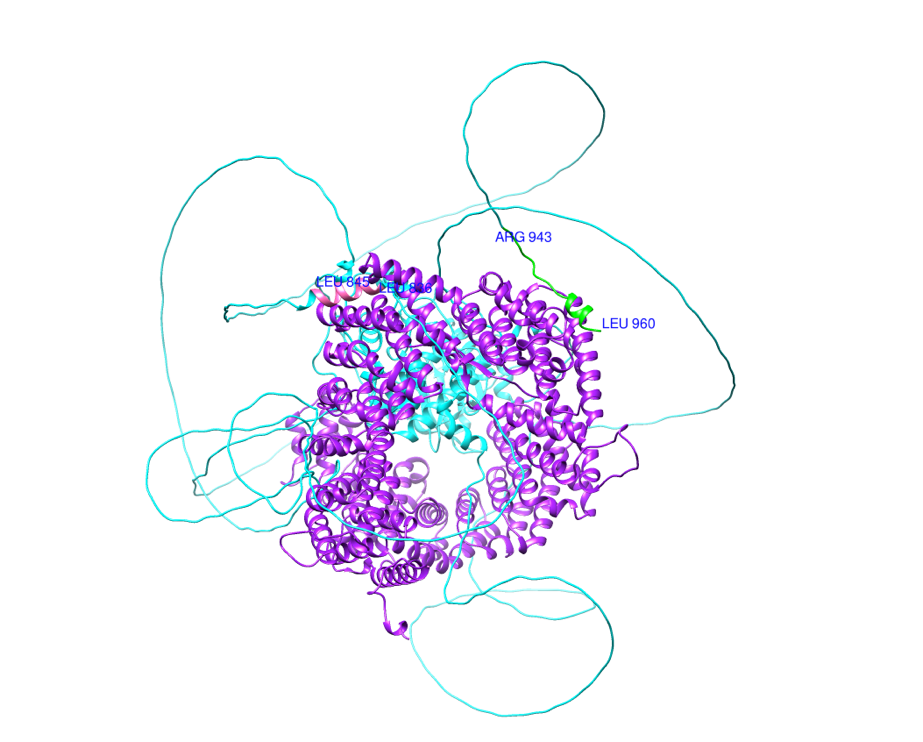

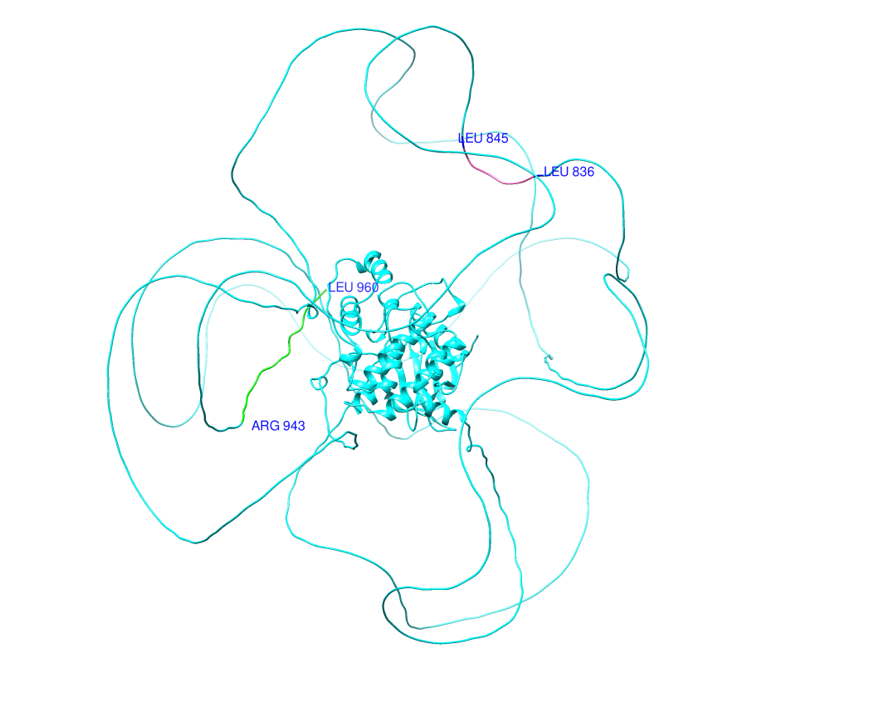


**Figure S5**. The effect of the CDKL5 *Arg943Asnfs*11* mutation on its interaction with a nuclear export receptor (Chromosome region maintenance 1 (CRM1). The three Alphafold3 [13] predicted structures are superimposed on the amino acids 1-305 of CDKL5, which form a verified secondary structure. Since CRM1-mediated nuclear export is dependent upon recognition of a nuclear export signal (NES), which is homologous to amino acids 836 to 845 (LKSLRKLLHL) of CDKL5 [31] (colored pink). The 836-845 (pink) and 943-960 (green) regions of CDKL5 monomer (Top Left) are predicted to be unstable; but when CDKL5 interacts with CRM1, α-helices are formed in both regions, which may help CRM1 (purple) to recognize the NES that is required for transport of CDKL5 (Top Right). However, the α-helix at the C terminus of CDKL5 disappears in the *Arg943Asnfs*11* CDKL5 protein and the length and stability of the 836-845 α-helix decreases. This could reduce the CDKL5 recognition by CRM1, which would reduce the amount of cytoplasmic CDLK5.
