# Supplementary material for: Genetic Diagnosis and Discovery Enabled by Large Language Models
Source: Adv Sci (Weinh). 2026 Feb 8;13(22):e18656. doi: 10.1002/advs.202518656 (PMC13088295; doi:10.1002/advs.202518656)
Supplement: Supplementary file 2 — Supporting File 2: advs74268‐sup‐0002‐SupplementalDataFile1.docx. [file ADVS-13-e18656-s011.docx]

**Supplemental Data File 1**

Med-PaLM 2 input and output for mouse genes associated with five phenotypes

**Candidate gene set 1: Cataract**

**Input prompt:** You are a helpful genetic assistant. The following is a question about genetic discovery. Which of the following genes is most likely associated with phenotype Cataract.

Gene candidates*: Miox, Nid1, Trappc1, Pi4ka, Adgrv1, Slc8b1*

**Med-PaLM 2 output:**

Of the genes listed, the most likely candidate for association with cataract is **Nid1**. Nid1 encodes nidogen-1, a protein that is a major component of the extracellular matrix in the eye. Mutations in Nid1 have been shown to cause a variety of eye disorders, including cataracts. For example, a study of families with autosomal dominant cataracts found that mutations in Nid1 were responsible for the condition in some of the families. The other genes listed are not known to be associated with cataracts. Miox encodes a protein that is involved in the metabolism of iodine. Trappc1 encodes a protein that is involved in the transport of proteins. Pi4ka encodes a protein that is involved in the production of phosphatidylinositol 4-phosphate. Adgrv1 encodes a protein that is involved in the development of blood vessels. Slc8b1 encodes a protein that is involved in the transport of calcium. While it is possible that mutations in any of these genes could contribute to the development of cataracts, there is no evidence to suggest that they are directly involved in the condition. Therefore, the most likely candidate for association with cataract is Nid1.

**Candidate gene set 2: Diabetes**

**Input prompt:** You are a helpful genetic assistant. The following is a question about genetic discovery. Which of the following genes is most likely associated with phenotype Diabetes.

Gene candidates: *Krtap5-1, Act1, Act2, Act3, Actb, Abra, Tlr5*, *Myh1, Myh2, Myh8, Unc45a, Cdc102a*

**Med-PaLM 2 output:**

Of the genes listed, the most likely candidate for association with diabetes is **Tlr5**. Tlr5 is a Toll-like receptor that is involved in the innate immune system. It is known to play a role in the recognition of bacterial flagellin, a component of bacterial flagella. Activation of Tlr5 can lead to the production of inflammatory cytokines, which can contribute to the development of insulin resistance. Insulin resistance is a condition in which the body's cells become less responsive to insulin. This can lead to high blood sugar levels, which is a hallmark of diabetes. Studies have shown that mice lacking Tlr5 are protected from developing insulin resistance. Additionally, polymorphisms in the Tlr5 gene have been associated with an increased risk of developing type 2 diabetes in humans. The other genes listed are not known to be directly involved in the development of diabetes. However, it is possible that they could play a role in the development of complications associated with diabetes. For example, Krtap5-1 is a gene that is involved in the formation of keratin, a protein that is found in hair, skin, and nails. People with diabetes are at increased risk of developing skin problems, so it is possible that Krtap5-1 could play a role in these complications. Overall, the evidence suggests that Tlr5 is the most likely candidate gene for association with diabetes. However, it is important to note that genetic factors are only one part of the puzzle when it comes to understanding the development of diabetes. Environmental factors, such as diet and lifestyle, also play a role.

**Candidate Gene Set 3: Chronic Pain**

**Input prompt:** You are a helpful genetic assistant. The following is a question about genetic discovery. Which of the following genes is most likely associated with phenotype Chronic pain. Gene candidates: *P2rx7, Arntl, Pttg1, Kcnk2, Clcn4-2, Mhrt, Mh7b, Cttnb1, Dact, Cdh2*

**Med-PaLM 2 output:**

Of the genes listed, P2rx7 is the most likely to be associated with chronic pain. P2rx7 is a gene that encodes for a receptor protein that is involved in the transmission of pain signals. Studies have shown that mutations in the P2rx7 gene can lead to increased pain sensitivity. Additionally, P2rx7 has been shown to be upregulated in the spinal cord of patients with chronic pain. The other genes listed are not as well-studied in the context of chronic pain. However, some of them have been implicated in other pain-related conditions. For example, Arntl has been linked to migraine headaches, and Kcnk2 has been linked to neuropathic pain. Overall, the evidence suggests that P2rx7 is the most likely of the listed genes to be associated with chronic pain. However, it is important to note that chronic pain is a complex condition that is likely influenced by multiple genes. Therefore, it is possible that other genes on the list may also play a role in chronic pain. If you are interested in learning more about the genetics of chronic pain, I would be happy to provide additional information.

*Comment on this analysis*: While Med-PaLM 2 correctly identified the experimentally verified gene (*P2rx7)* ^8^ in response to the chronic pain query, it also mentioned two other genes whose potential relationship to chronic pain we had not previously considered: (i) *Kcnk2* is a potassium channel that effects multiple types of pain responses ^9-11^; and (ii) *Arntl,* which is a major circadian clock regulator ^12^, because maladaptive pain is affected by circadian rhythm disruption ^13^. Although the connection between *Arntl* and chronic pain is tenuous, the hypothesis that *Kcnk2* alleles could impact chronic pain is a creative and intriguing one.

**Candidate Gene Set 4: Albino Skin**

**Input prompt:** You are a helpful genetic assistant. The following is a question about genetic discovery. Which of the following genes is most likely associated with phenotype Albino skin.

Gene candidates: *Grm5, Fzdr, Dlg2, Tyr, Polc4, Sec11a, Ap3b2, Rab38, Tmem135, Cedc81, 17rn6, Fcd, Picalm, Cdc83, Nrs155*

**Med-PaLM 2 output:**

The gene most likely associated with the albino skin phenotype is **Tyr**. The Tyr gene encodes the tyrosinase enzyme, which is responsible for the production of melanin. Melanin is the pigment that gives skin, hair, and eyes their color. Mutations in the Tyr gene can lead to a lack of melanin production, resulting in albinism. The other genes listed are not known to be associated with albinism. However, some of them may play a role in other aspects of skin pigmentation. For example, the Grm5 gene is involved in the production of melanocytes, which are the cells that produce melanin. Mutations in the Grm5 gene can lead to a condition called piebaldism, which is characterized by patches of white skin. If you are concerned about a genetic condition, it is important to speak with a doctor or genetic counselor. They can provide you with more information about the condition and its inheritance pattern. They can also order genetic testing to confirm a diagnosis.

**Candidate Gene Set 5: Warfarin Metabolism**

**Input prompt:** You are a helpful genetic assistant. The following is a question about genetic discovery. Which of the following genes is most likely associated with phenotype Warfarin metabolism.

Gene candidates: *Igsf8, Kcnj9, Kcnj10, Slc30a1, Icnh1, Masp2, Itpr1, Grin2b, Ctsc, Art5, Trpc2, Rrm1, Adam9, Lpl, Mmp2, Nnmt, Acy1, Gabrg2, Ctsl, Adprtl2, Abhd4, Mmp14, Psmb5, cacna1i, Tmprss2, Sncaip, Aldh7a1, Cyp2c55, Abcc2, Gabre*

**Med-PaLM 2 output:**

Out of the genes you listed, the most likely gene associated with warfarin metabolism is **CYP2C9**. This gene encodes an enzyme that is responsible for the metabolism of warfarin. Variations in the CYP2C9 gene can affect how quickly or slowly a person metabolizes warfarin. This can impact the dose of warfarin that a person needs to take in order to achieve the desired effect. Other genes that may be associated with warfarin metabolism include VKORC1, CYP4F2, and CYP2C19. However, CYP2C9 is the most well-studied and has the strongest association with warfarin metabolism. It is important to note that genetic factors are not the only factors that can affect warfarin metabolism. Other factors, such as age, diet, and medications, can also play a role. Therefore, it is important for people taking warfarin to be monitored closely by their healthcare provider. I hope this information is helpful. Please let me know if you have any other questions.

Comment: *Cyp2c55* is located within a haplotype block that contains murine *Cyp29*, which is the homolog of human *CYP2C29*.

**Candidate Gene Set 6: Aromatic hydrocarbon response**

**Input prompt:** You are a helpful genetic assistant. The following is a question about genetic discovery. Which of the following genes is most likely associated with phenotype Aromatic hydrocarbon response. Gene candidates: *Ahr, Dnpep, Dusp2, Vdr, Ren1, Cyp2b19, Pola1, Esr2*

**Med-PaLM 2 output:**

The gene most likely associated with the phenotype Aromatic hydrocarbon response is **Ahr**. The Ahr gene encodes the aryl hydrocarbon receptor, which is a transcription factor that regulates the expression of genes involved in the metabolism of aromatic hydrocarbons. Aromatic hydrocarbons are a class of chemicals that are found in many environmental pollutants, such as cigarette smoke and car exhaust fumes. When Ahr is activated by binding to an aromatic hydrocarbon, it can induce the expression of genes that help to detoxify these chemicals. Studies have shown that mutations in the Ahr gene can lead to altered responses to aromatic hydrocarbons. For example, people with certain mutations in the Ahr gene may be more susceptible to the harmful effects of exposure to these chemicals. The other genes listed are not as likely to be associated with the phenotype Aromatic hydrocarbon response. However, some of these genes may play a role in the metabolism of aromatic hydrocarbons or in the response to these chemicals. For example, the Cyp2b19 gene encodes an enzyme that is involved in the metabolism of some aromatic hydrocarbons. Overall, the Ahr gene is the most likely candidate gene for the phenotype Aromatic hydrocarbon response.
