# Supplementary material for: Genetic Diagnosis and Discovery Enabled by Large Language Models
Source: Adv Sci (Weinh). 2026 Feb 8;13(22):e18656. doi: 10.1002/advs.202518656 (PMC13088295; doi:10.1002/advs.202518656)

**Supplemental Data File 2**. Genes with NOD/LtJ-specific variant alleles that cause high-impact changes in the encoded proteins. The gene symbol, variant, chromosomal location, and impact of the NOD/LtJ variant are shown. Variants located within short highly polymorphic sequences that are used as genotyping markers, immunoglobulin variable region genes, or those affecting splice sites were removed from further analysis; while the 14 genes analyzed by Med-PaLM 2 are shown in bold.


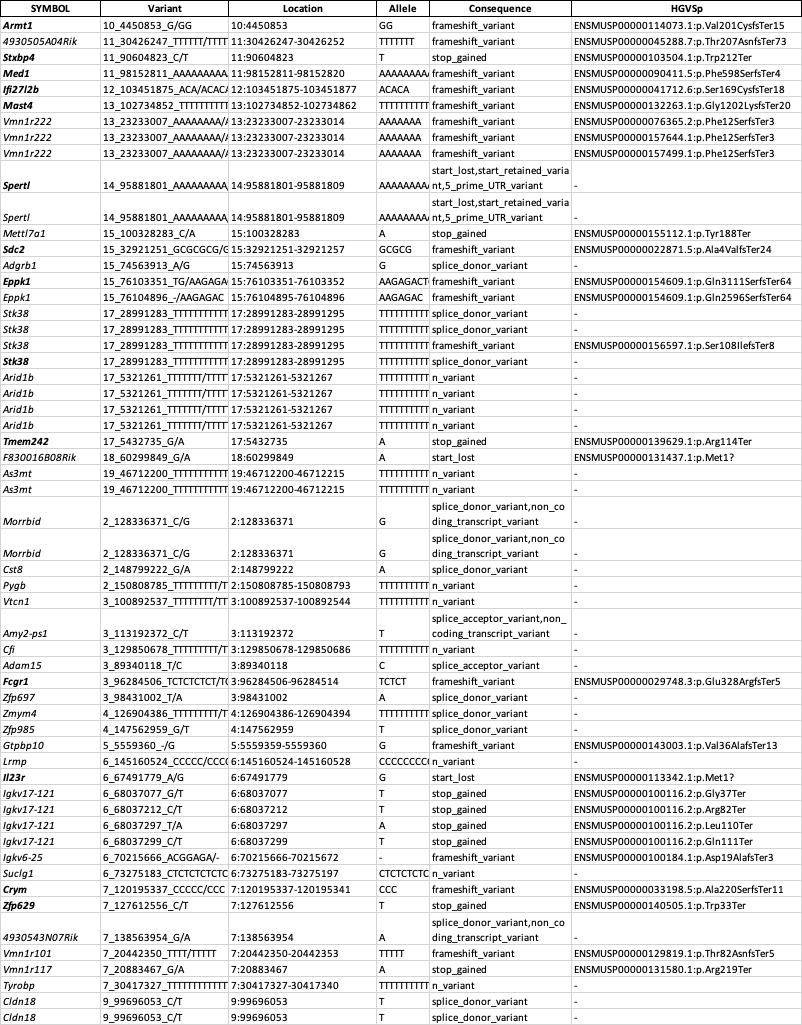

Supplement: Supplementary file 3 — Supporting File 3: advs74268‐sup‐0003‐SupplementalDataFile2.docx. [file ADVS-13-e18656-s003.docx]
