# Supplementary material for: Genetic Diagnosis and Discovery Enabled by Large Language Models
Source: Adv Sci (Weinh). 2026 Feb 8;13(22):e18656. doi: 10.1002/advs.202518656 (PMC13088295; doi:10.1002/advs.202518656)
Supplement: Supplementary file 4 — Supporting File 4: advs74268‐sup‐0004‐SupplementalDataFile3.docx. [file ADVS-13-e18656-s010.docx]

**Supplemental Data File 3**

**Chain of thought (COT) prompt and Med-PaLM 2 identification**

**of murine genes for hearing loss**

*COT prompt*: You are a helpful genetic assistant. You need to identify genes with causal mutations for hearing loss or deafness. You are provided with a set of genes that contain causal genetic factors in mice that might cause hearing loss or deafness. You need to determine which of the following genes have causal mutations that are directly associated with hearing loss or deafness in mice or humans. Output the top 5 options in the final answer.

(1) Zfp629 (2) Sdc2 (3) Gtpbp10 (4) Adgrb1 (5) Mettl7a1 (6) Il23r (7) Crym (8) Med1 (9) Stk38 (10) Fcgr1 (11) Armt1 (12) Mast4 (13) Spertl (14) Ifi27l2b (15) Tmem242 (16) Stxbp4 (17) Eppk1.

**Med-PaLM 2 output:** Based on the available evidence, the top 5 genes in the list that are most likely to have causal mutations that are directly associated with hearing loss or deafness in mice or humans are:

**Crym**: Crym encodes a protein called α-crystallin, which is a major structural component of the lens. Mutations in Crym can lead to cataracts, which can cause hearing loss.

**Med1**: Med1 encodes a protein that is involved in the development of the inner ear. Mutations in Med1 can lead to sensorineural hearing loss.

**Mast4**: Mast4 encodes a protein that is involved in the transmission of sound signals from the inner ear to the brain. Mutations in Mast4 can lead to nonsyndromic hearing loss.

**Tmem242**: Tmem242 encodes a protein that is involved in the development of the inner ear. Mutations in Tmem242 can lead to sensorineural hearing loss.

**Stk38**: Stk38 encodes a protein that is involved in the development of the inner ear. Mutations in Stk38 can lead to sensorineural hearing loss.

It is important to note that hearing loss is a complex condition that is likely influenced by multiple genes. Therefore, it is possible that other genes on the list may also play a role in hearing loss. Newer studies might have identified more direct links or clarified associations for the genes mentioned. Always refer to recent literature for the most up-to-date scientific evidence.
