# Supplementary material for: Genetic Diagnosis and Discovery Enabled by Large Language Models
Source: Adv Sci (Weinh). 2026 Feb 8;13(22):e18656. doi: 10.1002/advs.202518656 (PMC13088295; doi:10.1002/advs.202518656)
Supplement: Supplementary file 8 — Supporting File 8: advs74268‐sup‐0008‐SupplementalDataFile7.docx. [file ADVS-13-e18656-s002.docx]

**Supplemental Data File 7**

*Hypothesis generation prompt:* You are a helpful genetic assistant. You need to identify genes with causal mutations for these abnormalities for a patient. You are provided with a summary of all the phenotypic abnormalities of the patient, along with a single gene that may have a mutation that might cause the abnormalities. You are also given access to web search results related to the gene. Your task is to generate 5 hypotheses for why the gene is causal to the patient’s case. Make sure each hypothesis is thorough, providing detailed justifications for why the gene is causal to each relevant phenotypic abnormality. Based on the current patient information and the gene, find the top 5 possible hypotheses.

***Case Summary for Genetic Patient 1***: The patient is now a **-year-old male/female who presented with a history of unexplained central nervous system inflammatory disease.  Additional history included idiopathic pulmonary fibrosis, bilateral uveitis, and pericarditis with myocarditis. The patient had toxoplasmosis at age 9. Pulmonary symptoms were noted early in life with a diagnosis of pulmonary fibrosis at age 13, and an episode of aseptic meningitis at this age.  Uveitis was recognized at age 18 along with myocarditis and pericarditis.  At age 31 s/he developed seizures and was noted to have enhancing parenchymal lesions on MRI of the brain and multifocal leptomeningitis.  The evaluation included a brain biopsy, which showed encephalitis.

The laboratory evaluation has included negative CBC, PT/PTT/INR, BMP, ESR, CRP, TSH/T4, ACE, iron, cANCA, pANCA, cortisol, antiPL, Gliadin, dsDNA, RNP, ssA/B, TPO, SCL-70, MPO, TTG, MMA, RPR, HIV1/2, HBV Ag, HCV, VZV, VDRL, paraneoplastic antibody panel, Lyme serologies, Whipple, toxoplasmosis IgM^-^, IgG^+^ and H pylori antibody.

*List of Human Phenotype Ontology Terms*: HP:0001701 Pericarditis HP:0009830 Peripheral neuropathy HP:0012819 Myocarditis HP:0001250 Seizure HP:0010562 Keloids HP:0012051 Reactive hypoglycemia HP:0001187 Hyperextensibility of the finger joints HP:0001382 Joint hypermobility HP:0100759 Clubbing of fingers HP:0002206 Pulmonary fibrosis HP:0000218 High palate HP:0000554 Uveitis HP:0001287 Meningitis HP:0010651 Abnormal meningeal morphology

**List of Gene Candidates:**

(1) ADGRV1 (2) KIF3B (3) SEMA4C (4) ARL5C (5) BCAS3 (6) BCL11A (7) HIP1 (8) MACF1 (9) ORAI2 (10) BCL3 (11) NEK8 (12) ACSM1 (13) ABHD15 (14) ABCA7 (15) SPRR1B (16) VWCE (17) HRCT1 (18) BCAR1 (19) TRIP10 (20) ZNF674 (21) DNAAF1 (22) MAP4K2 (23) SLC47A2 (24) ADAM15 (25) ZNF776 (26) FDFT1 (27) HLA-A (28) GREB1 (29) PRSS22 (30) UNC13C (31) PKD1 (32) DDX11 (33) MRPS5 (34) APOB (35) RGS4 (36) RGMA (37) SCN10A (38) VIPR2 (39) SLC4A7 (40) PPP1R3A (41) MAP2K4 (42) GPANK1 (43) MYH10 (44) HIST1H1C (45) TNFSF12 (46) PGM2L1 (47) ANKLE2 (48) RP11-293B20.2 (49) MAS1 (50) AFDN (51) FAM83G (52) IL17RD (53) MAD1L1 (54) EP400 (55) MUC4 (56) PHLPP1 (57) UTP20 (58) SRBD1 (59) IGHV3-38 (60) TMPRSS12 (61) SLC24A2 (62) MGAT3 (63) NAA15 (64) CACNA1H (65) NECTIN2 (66) FBXO17 (67) GDF6 (68) SNAPC1 (69) PPP6R1 (70) ZNF713 (71) PLEKHB2 (72) NEDD4L (73) PKN1 (74) TMCC3 (75) SGSM1 (76) CASD1 (77) GLI2 (78) CEP131 (79) AHNAK (80) DRAXIN (81) CKMT2 (82) MIGA2 (83) CLUH (84) SLC22A10 (85) IRAK4 (86) CRIPAK (87) ALOX5AP (88) OBSCN (89) CEP72 (90) ZKSCAN5 (91) PTPRF (92) SAMD3 (93) GPRIN3 (94) STK10 (95) TALDO1 (96) PDE4DIP (97) RNF103 (98) PEX12 (99) DIP2B (100) ANKRD16 (101) PCED1A (102) OAZ1 (103) TARBP2 (104) BAGE2 (105) NOTCH2 (106) CASZ1 (107) NUDT4B (108) CHRNA10 (109) WDFY3 (110) PNMA5 (111) GABRD (112) CDC42EP4 (113) IGHG1 (114) FBXL4 (115) DYSF (116) LRRK1 (117) LRRC75B (118) CPNE7 (119) MUC2 (120) FADS6 (121) PANK4 (122) EEF1AKNMT (123) ZNF420 (124) XKR8 (125) AFAP1L1 (126) SORCS2 (127) MIRLET7BHG (128) FLCN (129) FBH1 (130) SETD5 (131) TFR2 (132) AEBP1 (133) BRAT1 (134) DTX1 (135) SULT1A2 (136) ATXN3 (137) SLC36A2 (138) HS3ST2 (139) SORL1 (140) TMPRSS11F (141) DLGAP5 (142) WDPCP (143) HSPG2 (144) DUOX2 (145) NBPF10 (146) LNP1 (147) LRIF1 (148) FAT4 (149) KLHL15 (150) MC1R (151) DSCAML1 (152) ACKR4 (153) TRIOBP (154) EXOC4 (155) ATRX (156) PALLD (157) FAM193B (158) MRNIP (159) AC018696.2 (160) LMNB1 (161) KLHL31 (162) NOX4 (163) ASS1 (164) CDH23 (165) TP53BP1 (166) CDK5RAP2 (167) SUSD1 (168) EPPK1 (169) MAP1A (170) FRG1JP (171) CIC (172) DENND1A (173) JMJD7-PLA2G4B (174) TEX35 (175) MCPH1 (176) PIAS4 (177) PAMR1 (178) VIT (179) SMARCD3 (180) FANK1 (181) AMIGO3 (182) MMRN2 (183) RASAL2 (184) ASCC2 (185) ELN (186) KRBA1 (187) PRKAG1 (188) FETUB (189) NKD1 (190) CRB1 (191) ANKRD13A (192) ZDHHC3 (193) NEPRO (194) ST8SIA4 (195) SREBF1 (196) SF3B3 (197) FZD1 (198) ABCB5 (199) KCNT1 (200) RUVBL2 (201) OR52N5 (202) HLA-J (203) NRK (204) HRNR (205) CRIM1 (206) SAMD1 (207) ZNF606 (208) TTC28 (209) DHX38 (210) PCDHB10 (211) GJD3 (212) ASXL3 (213) MRGPRE (214) DCP2 (215) QSOX1 (216) AIFM3 (217) KCNK6 (218) C16orf58 (219) PTCD1 (220) UBASH3A (221) RABEP2 (222) NUDT4 (223) MTSS1 (224) TCF3 (225) PIDD1 (226) TARS2 (227) OPTC (228) CDKL2 (229) ALS2CR12 (230) ARHGEF11 (231) PKHD1 (232) RANBP3L

*Med-Gemini output:*

1. Loss-of-function mutations in IRAK4 lead to impaired TLR/IL-1R signaling, increasing susceptibility to infections like toxoplasmosis (experienced by the patient at age 9) and impairing immune regulation, contributing to the development of autoinflammatory manifestations such as uveitis, pericarditis, myocarditis, and CNS inflammation. The chronic inflammation could then lead to pulmonary fibrosis.
2. A hypomorphic IRAK4 mutation results in partial impairment of TLR/IL-1R signaling, leading to an inadequate initial immune response to infections, potentially contributing to the early onset of pulmonary symptoms and later diagnosis of pulmonary fibrosis. This partial impairment could also dysregulate the immune system, leading to autoinflammatory diseases like uveitis, pericarditis, myocarditis, and CNS inflammation manifesting as seizures and encephalitis.
3. A dominant-negative IRAK4 mutation interferes with the function of wild-type IRAK4, leading to a more severe impairment of TLR/IL-1R signaling than a simple loss-of-function mutation. This could explain the early-onset and severe nature of the patient’s various inflammatory conditions, including pulmonary fibrosis, uveitis, pericarditis, myocarditis, and CNS manifestations.
4. An IRAK4 mutation specifically affects its kinase domain, disrupting downstream signaling pathways involved in both innate and adaptive immunity. This could lead to a combined immunodeficiency with increased susceptibility to infections (toxoplasmosis) and immune dysregulation, leading to autoinflammatory manifestations in multiple organs, including the lungs (pulmonary fibrosis), eyes (uveitis), heart (pericarditis, myocarditis), and brain (encephalitis, seizures).
5. The IRAK4 mutation affects its interaction with other adaptor proteins in the TLR/IL-1R signaling pathway, leading to aberrant downstream signaling and an imbalance between pro- and anti-inflammatory responses. This could explain the complex clinical picture with both infectious and autoinflammatory manifestations.

***----------------------------------------------------------------------------------------------------------------------------***

***Grounding and scoring for genetic diagnosis with Med Gemini***

*Scoring prompt:* You are a helpful genetic assistant analyzing a hypothesis. You are given a summary of all the phenotypic abnormalities of a patient. Your task is to analyze a hypothesis about why a certain gene is causal to the patient’s case. You are also given the most relevant research articles related to the given hypothesis.

When analyzing the hypothesis, follow the following instructions:

1. Write detailed aspects of how the abstracts either support or oppose the hypothesis.
2. Score the hypothesis based on the supporting and opposing aspects from the abstracts using a scale from 1 to 5, where a score of 1 means that the abstracts oppose the hypothesis, and a score of 5 means that the abstracts strongly support the hypothesis.

Patient Case Summary: {case summary}

List of Human Phenotype Ontology Terms: {human phenotype terms}

Abstracts: {abstracts}

Hypothesis: Loss-of-function mutations in IRAK4 lead to impaired TLR/IL-1R signaling, increasing susceptibility to infections like toxoplasmosis (experienced by the patient at age 9) and impairing immune regulation, contributing to the development of autoinflammatory manifestations such as uveitis, pericarditis, myocarditis, and CNS inflammation. The chronic inflammation could then lead to pulmonary fibrosis.

*Med-Gemini output:*

Supporting/opposing evidence from abstracts

- Abstract: Listeria Monocytogenes Meningoencephalitis Due to IRAK4 Deficiency., published in Journal of clinical immunology (2021-10-01). Supporting evidence: The patient in this abstract suffered recurrent infections and developed meningoencephalitis due to IRAK4 deficiency, directly supporting the increased susceptibility to infections and CNS involvement proposed in the hypothesis.
- Abstract: Impaired innate immunity in mice deficient in interleukin-1 receptor-associated kinase 4 leads to defective type 1 T cell responses, B cell expansion, and enhanced susceptibility to infection with Toxoplasma gondii., published in Infection and immunity (2012-12-01). Supporting evidence: This article demonstrates that IRAK4 is necessary for an effective immune response against T. gondii, thus directly supporting the hypothesis by showing its role in increased susceptibility to and impaired immunity against infections.
- Abstract: Intraocular Inflammation Associated with IRAK4 Deficiency., published in Ocular immunology and inflammation (2023-04-21). Supporting evidence: The presence of uveitis, seizures, and leptomeningeal enhancement in a patient with IRAK4 deficiency strongly supports the link between IRAK4 deficit and autoinflammatory manifestations, specifically targeting the eye and CNS, as observed in our patient.
- Abstract: Activated PI3 Kinase Delta Syndrome Revealed by Vasculitis and Disseminated Toxoplasmosis., published in Journal of clinical immunology (2022-04-01). Opposing evidence: While this abstract illustrates a link between immune deficiency (APDS-1) and disseminated toxoplasmosis and also mentions a previously reported case of APDS2 with a similar clinical presentation, neither patient presented with autoinflammatory conditions such as uveitis, pericarditis, myocarditis, and CNS inflammation, which are key features of our patient, and it doesn’t directly implicate IRAK4.
- Abstract: Interleukin-1 receptor associated kinase 4 deficiency: a case report and literature review, published in Zhonghua er ke za zhi = Chinese journal of pediatrics (2021-10-02). Opposing evidence: Although this review acknowledges rare cases of autoimmune diseases in IRAK4 deficiency, they primarily emphasize recurrent bacterial infection as the core phenotype. This focus leans against the hypothesis’s assertion about the prominence of autoinflammatory manifestations in our patient’s presentation.

Score: 4/5

___________________________________________________________________________

The HPO terms and genes with VUS that were evaluated by the LLM for **Genetic Disease Patients 2 to 6** are listed in Supplemental Data File 6, and the LLM output for each patient is shown in Supplemental Data File 8. The prompt text used for analysis of genetic patients 2 to 6 are shown below. For some cases, the text was altered so as not to reveal the sex of the patient.

**Case Summary for Genetic Patient 2:** A young boy/girl with history of global developmental delay since infancy. S/He was delivered by C-section at 38 weeks due to decreased fetal movement and non-reassuring fetal heart tones. Polyhydramnios was noted at 24 weeks gestation. S/He additionally has a history of short stature, sagittal synostosis (dolichocephaly), unilateral choanal atresia, hypotonia, and 3 small VSDs. S/He has been evaluated for atypical movements that have been considered as possibly athetosis. S/He also has a history of bilateral undescended testes and was noted to be hypotonic in the newborn period. S/He has sparse hair and eyebrows since birth. S/He has 11 paired ribs. A brain MRI showed multiple small areas of T2 hyperintensity in the cortical white matter. His/her exam was notable for sparse hair, down-slanted palpebral fissures, facial hypotonia, overlapping toes, small 2^nd^ and 5^th^ nails on the hands, and low axial tone.

**Case Summary for Genetic Patient 3**: A young boy/girl with a history of infantile onset developmental epileptic encephalopathy with associated intellectual disability and autism spectrum disorder. Seizures were first noted at 7 months of age. S/he walked at 13 months of age and developed a pincer grasp at age two. S/he is non-verbal. He/Her exam has been notable for a high palate, long eyelashes, a café au lait spot, joint hypermobility, small hands and feet and small head. MRI of the brain at age 7 months was normal.

**Case Summary for Genetic Patient 4**: An adult with a history of muscular weakness of the proximal arms and legs beginning at age 24 that has been progressive. EMG was read as showing possible myositis. Treatment with prednisone did not result in improvement. CK has been elevated to 2400. Echocardiogram showed an ejection fraction of 44%. Muscle biopsy at age 28 showed possible myotilinopathy and dysferlinopathy. MRI at age 30 showed atrophy of the proximal muscles of the leg. At age 33 s/he developed weakness of the facial muscles and began to have difficulty rising from a seated position.

**Case Summary for Genetic Patient 5**: A young boy/girl with global developmental delay first noted in early infancy. Myoclonic epilepsy was diagnosed at 14 months of age. S/he also has a history of central hypotonia and gastrointestinal symptoms including reflux and constipation. S/he is non-verbal and can sit with assistance. S/He rolled over at 15 months of age.

**Case Summary for Genetic Patient 6:** A young girl with a history of dysmorphic features, hypotonia and developmental delay as well as developmental regression. She had head control at two months and started taking solids at 6 months. Subsequently, she lost these skills and has been g-tube dependent. She has also been noted to have exotropia, lack of tear production, late eruption of teeth with early deciduous tooth loss, a laryngeal cleft and pulmonary insufficiency with oxygen dependence after multiple pneumonias. She had premature adrenarche at age 3 and has had two episodes of pancreatitis. MRI has shown increased T2 signal in the basal ganglia and decreased white matter volume. She experienced a febrile seizure in toddlerhood.

Her exam is notable for synophyrs, low anterior hairline, patch of hair at her right temple, an area of coarser hair at posterior scalp, overlapping toes, prominent lower lip, prominent mandible, mild pectus excavatum and long eye lashes.
