# Supplementary material for: Genetic Diagnosis and Discovery Enabled by Large Language Models
Source: Adv Sci (Weinh). 2026 Feb 8;13(22):e18656. doi: 10.1002/advs.202518656 (PMC13088295; doi:10.1002/advs.202518656)
Supplement: Supplementary file 10 — Supporting File 10: advs74268‐sup‐0010‐SupplementalDataFile9.docx. [file ADVS-13-e18656-s004.docx]

**THE FOUR PROMPTS AND OUTPUT FROM FOUR LLMS**

**Prompt 1** **(Mouse Cataract):**

# You are a helpful genetic assistant. The following is a question about genetic discovery. Which of the following genes is most likely associated with phenotype Cataract.

Gene candidates*: Miox, Nid1, Trappc1, Pi4ka, Adgrv1, Slc8b1*

# **Open AI GPT 4.5 Response**:

The gene **Miox** (Myo-inositol oxygenase) is most likely associated with the phenotype **Cataract** among the given candidates.

**Reasoning:** Mutations in the **Miox** gene have been specifically linked to congenital cataracts in several genetic studies and databases. MIOX plays a critical role in the metabolism of myo-inositol, and disruptions in this pathway have been implicated in lens transparency maintenance and cataract formation

In contrast, the other listed genes (**Nid1, Trappc1, Pi4ka, Adgrv1, Slc8b1**) are typically

associated with other phenotypes or disorders:

**Nid1:** Typically related to basement membrane defects, developmental abnormalities.
**Trappc1:** Associated with trafficking and vesicle transport; mutations commonly cause neurological phenotypes.
**Pi4ka:** Related to phosphoinositide metabolism; mutations are usually neurological or developmental.
**Adgrv1:** Commonly associated with Usher syndrome, characterized by hearing loss and retinitis pigmentosa.
**Slc8b1:** Involved in sodium-calcium exchange; mutations more commonly cause neurological or cardiac phenotypes.

Thus, **Miox** is the strongest candidate for cataracts.

# **Open AI o3-mini-high Response:**

The gene most likely associated with a cataract phenotype is **Nid1**.

**Explanation: Nid1** encodes Nidogen-1, a crucial component of the basement membrane. Since the lens capsule is a specialized basement membrane, defects in its structure can contribute to cataract formation.

The other gene candidates (Miox, Trappc1, Pi4ka, Adgrv1, Slc8b1) are not primarily linked with lens structural integrity. For instance, Adgrv1 is more commonly associated with sensory disorders (like Usher syndrome), and the others have different cellular functions not directly tied to lens transparency.

Thus, based on its role in basement membrane composition and the structure of the lens capsule, Nid1 is the strongest candidate for being associated with cataract.

link, particularly concerning diabetic cataracts, due to its role in metabolic pathways affected by glucose levels and oxidative stress. However, the evidence for *Nid1* involves a specific genetic mutation identified as causing the cataract phenotype.

# **Deepseek R1 Response:**

The gene most likely associated with cataracts among the candidates provided is Nid1.

Key reasoning:

- Nid1 (Nidogen 1) is a critical component of the extracellular matrix and basement membranes, which are essential for maintaining the structural integrity of the lens. Disruption of basement membrane proteins can lead to lens defects, such as improper cell organization or protein aggregation, directly contributing to cataract formation.
- Experimental evidence, including studies in knockout mice, has shown that loss of Nid1 results in structural abnormalities in the lens and cataract development.
- Other genes like Slc8b1 (calcium homeostasis) or Miox (inositol metabolism) may indirectly influence cataract pathways, but Nid1 has a more established and direct role in lens biology and cataract pathology.

Conclusion: Nid1 is the strongest candidate based on its role in basement membrane integrity and experimental data linking its dysfunction to cataracts.

**Claude Sonnet 3.7 Response**:

Based on the gene candidates you've provided, ADGRV1 is most likely associated with cataracts.

ADGRV1 (Adhesion G Protein-Coupled Receptor V1) has been linked to several ocular conditions. It encodes a protein that's important for the development and maintenance of the inner ear and retina. Mutations in ADGRV1 have been associated with Usher syndrome, which can include retinal degeneration, and there are reports connecting it to congenital cataracts in some patients.

The other candidates have less established connections to cataracts:

- MIOX (Myo-Inositol Oxygenase) is primarily involved in kidney metabolism
- NID1 (Nidogen 1) is a basement membrane protein
- TRAPPC1 (Trafficking Protein Particle Complex 1) is involved in vesicle trafficking
- PI4KA (Phosphatidylinositol 4-Kinase Alpha) functions in lipid signaling
- SLC8B1 (Solute Carrier Family 8 Member B1) is a sodium/calcium exchanger

If you're investigating genetic causes of cataracts, ADGRV1 would be the most promising candidate from this list to explore further.

# **Prompt 2** (***Murine Genes for hearing loss):***

*COT prompt: You are a helpful genetic assistant. You need to identify genes with causal mutations for hearing loss or deafness. You are provided with a set of genes that contain causal genetic factors in mice that might cause hearing loss or deafness. You need to determine which of the following genes have causal mutations that are directly associated with hearing loss or deafness in mice or humans. Output the top 5 options in the final answer.*

*(1) Zfp629 (2) Sdc2 (3) Gtpbp10 (4) Adgrb1 (5) Mettl7a1 (6) Il23r (7) Crym (8) Med1 (9) Stk38 (10) Fcgr1 (11) Armt1 (12) Mast4 (13) Spertl (14) Ifi27l2b (15) Tmem242 (16) Stxbp4 (17) Eppk1.*

# **Claude Sonnet 3.7 Response:**

I'll carefully analyze each gene to identify which ones have causal mutations directly associated with hearing loss or deafness in mice or humans, thinking through this step-by-step.

Let me evaluate each gene:

1. Zfp629 (Zinc finger protein 629): While zinc finger proteins can be involved in gene regulation, I don't find strong evidence that Zfp629 specifically is causally linked to hearing loss.
2. Sdc2 (Syndecan-2): This gene encodes a transmembrane heparan sulfate proteoglycan. I don't see established evidence for a direct causal role in hearing loss.
3. Gtpbp10 (GTP-binding protein 10): This is involved in ribosome biogenesis, but I don't find evidence of a direct causal role in hearing loss.
4. Adgrb1 (Adhesion G protein-coupled receptor B1): This gene has roles in brain development, but I don't find clear evidence linking it directly to hearing loss.
5. Mettl7a1 (Methyltransferase-like 7A1): I don't see established evidence linking this gene directly to hearing loss.
6. Il23r (Interleukin-23 receptor): While involved in immune responses, I don't find evidence for a direct causal role in hearing loss.
7. Crym (Crystallin Mu): This gene encodes μ-crystallin, which binds thyroid hormone and is expressed in the inner ear. Mutations in CRYM have been reported to cause non-syndromic hearing loss in humans, and mouse models have shown that Crym is important for cochlear development and function.
8. Med1 (Mediator complex subunit 1): This gene is important for transcriptional regulation. Mouse models with Med1 deficiency have shown hearing impairment, suggesting a causal role in auditory function.
9. Stk38 (Serine/threonine kinase 38): I don't find strong evidence linking this gene causally to hearing loss.
10. Fcgr1 (Fc gamma receptor 1): While involved in immune function, I don't find evidence for a direct causal role in hearing loss.
11. Armt1 (Acidic residue methyltransferase 1): I don't find evidence linking this gene directly to hearing loss.
12. Mast4 (Microtubule associated serine/threonine kinase family member 4): I don't see established evidence linking this gene directly to hearing loss.
13. Spertl (Spermatogenesis and centriole associated 1): I don't find evidence linking this gene directly to hearing loss.
14. Ifi27l2b (Interferon alpha inducible protein 27 like 2B): I don't see established evidence linking this gene directly to hearing loss.
15. Tmem242 (Transmembrane protein 242): I don't find strong evidence linking this gene causally to hearing loss.
16. Stxbp4 (Syntaxin binding protein 4): I don't find evidence linking this gene directly to hearing loss.
17. Eppk1 (Epiplakin 1): This gene encodes a cytoskeletal linker protein. While expressed in various tissues, I don't find strong evidence for a direct causal role in hearing loss.

Based on my analysis, the most strongly evidenced genes with causal mutations directly associated with hearing loss or deafness are:

1. Crym - Associated with non-syndromic hearing loss in humans, with supporting mouse model evidence
2. Med1 - Mouse models with deficiency show hearing impairment

I don't find sufficient evidence to confidently identify three more genes from this list with established causal mutations for hearing loss. The evidence for the remaining genes is either lacking or not strong enough to establish a direct causal relationship with hearing loss or deafness in mice or humans.

#

# **Open AI o3-mini-high respons**e:

Based on current literature linking gene mutations to auditory dysfunction in mice and/or humans, the following five genes stand out as strong candidates:

- **Crym:** Mutations in CRYM have been reported in human cases of sensorineural hearing loss and mouse models support its role in auditory function.
- **Sdc2:** This cell‐surface proteoglycan is expressed in neural and inner ear tissues, and disruptions in its function have been implicated in auditory development defects.
- **Gtpbp10:** As a mitochondrial GTPase, GTPBP10 is thought to influence energy metabolism in high‐demand tissues like the cochlea; genetic disruptions here have been linked to hearing impairment in mouse studies.
- **Adgrb1:** This adhesion G protein–coupled receptor has been associated with vascular and neural functions in the inner ear; mutations in Adgrb1 have emerged from mutagenesis screens as a candidate for hearing loss.
- **Mast4:** Identified in mouse genetic screens, mutations in Mast4 have been connected to abnormal inner ear development and auditory deficits.

These five genes—CRYM, Sdc2, Gtpbp10, Adgrb1, and Mast4—represent the top options where causal mutations have been directly associated with hearing loss or deafness in mice or humans.

# **Open AI GPT 4.5 response**:

​Based on the available evidence, the following genes from your list have been identified to have causal mutations directly associated with hearing loss or deafness in mice or humans:​

1. **Adgrb1**: This gene encodes the adhesion G protein-coupled receptor B1 (BAI1). Studies have shown that BAI1 localizes AMPA receptors at the cochlear afferent post-synaptic density and is essential for hearing. Mice lacking full-length Adgrb1 exhibit early-onset hearing loss. ​[ScienceDirect+3NCBI+3Cell Press+3](https://www.ncbi.nlm.nih.gov/gene/107831?utm_source=chatgpt.com)
2. **Crym**: Mutations in the CRYM gene have been associated with nonsyndromic hearing loss in humans. Specifically, a missense variant (Pro51Leu) in CRYM was identified in a Chinese family, segregating with the hearing loss phenotype. ​[Nature+4PanelApp+4PMC+4](https://panelapp.genomicsengland.co.uk/panels/126/gene/CRYM/?utm_source=chatgpt.com)
3. **Med1**: While direct evidence linking Med1 mutations to hearing loss is limited, studies have indicated that mutations in Med1 can lead to sensorineural hearing loss. ​[PMC](https://pmc.ncbi.nlm.nih.gov/articles/PMC10659415/?utm_source=chatgpt.com)
4. **Stk38**: Mutations in Stk38 have been implicated in inner ear development, and such mutations can lead to sensorineural hearing loss. ​[PMC+1Nature+1](https://pmc.ncbi.nlm.nih.gov/articles/PMC10659415/?utm_source=chatgpt.com)
5. **Tmem242**: Mutations in Tmem242 have been associated with sensorineural hearing loss. ​[PMC](https://pmc.ncbi.nlm.nih.gov/articles/PMC10659415/?utm_source=chatgpt.com)

These genes have been identified through various studies as having a direct association with hearing loss or deafness in mice or humans.

# **Deepseek R1 Response**:

The top 5 genes with causal mutations directly associated with hearing loss or deafness in mice or humans from the provided list are:

Crym (7) Associated with progressive hearing loss in mice due to inner ear defects. Human homolog CRYM is linked to autosomal dominant nonsyndromic hearing loss (DFNA).

Stk38 (9) Mouse studies show Stk38 knockout leads to abnormal auditory brainstem responses, implicating its role in cochlear development via Hippo signaling.

Tmem242 (15) Mutations in TMEM242 are linked to human nonsyndromic hearing loss, and mouse models exhibit auditory deficits related to stereocilia organization.

Stxbp4 (16) Indirect evidence suggests involvement in synaptic vesicle trafficking in hair cells, though direct causal links are less established.

Eppk1 (17) Expressed in cochlear epithelial cells; structural defects in these cells could contribute to hearing loss, though specific mechanistic evidence is limited.

Final Answer: Note: Crym (7), Stk38 (9), and Tmem242 (15) have stronger evidence, while Stxbp4 (16) and Eppk1 (17) are included based on indirect or emerging studies.

#

# **Prompt 3 (Genetics Case 1):**

You are a helpful genetic assistant. You need to identify genes with causal mutations for these abnormalities for a patient. You are provided with a summary of all the phenotypic abnormalities of the patient, along with a single gene that may have a mutation that might cause the abnormalities. You are also given access to web search results related to the gene. Your task is to generate 5 hypotheses for why the gene is causal to the patient’s case. Make sure each hypothesis is thorough, providing detailed justifications for why the gene is causal to each relevant phenotypic abnormality. Based on the current patient information and the gene, find the top 5 possible hypotheses.

*Case Summary*: The patient is now a **-year-old male/female who presented with a history of unexplained central nervous system inflammatory disease. Additional history included idiopathic pulmonary fibrosis, bilateral uveitis, and pericarditis with myocarditis. The patient had toxoplasmosis at age 9. Pulmonary symptoms were noted early in life with a diagnosis of pulmonary fibrosis at age 13, and an episode of aseptic meningitis at this age. Uveitis was recognized at age 18 along with myocarditis and pericarditis. At age 31 s/he developed seizures and was noted to have enhancing parenchymal lesions on MRI of the brain and multifocal leptomeningitis. The evaluation included a brain biopsy, which showed encephalitis.

The laboratory evaluation has included negative CBC, PT/PTT/INR, BMP, ESR, CRP, TSH/T4, ACE, iron, cANCA, pANCA, cortisol, antiPL, Gliadin, dsDNA, RNP, ssA/B, TPO, SCL-70, MPO, TTG, MMA, RPR, HIV1/2, HBV Ag, HCV, VZV, VDRL, paraneoplastic antibody panel, Lyme serologies, Whipple, toxoplasmosis IgM^-^, IgG^+^ and H pylori antibody.

*List of Human Phenotype Ontology Terms*: HP:0001701 Pericarditis HP:0009830 Peripheral neuropathy HP:0012819 Myocarditis HP:0001250 Seizure HP:0010562 Keloids HP:0012051 Reactive hypoglycemia HP:0001187 Hyperextensibility of the finger joints HP:0001382 Joint hypermobility HP:0100759 Clubbing of fingers HP:0002206 Pulmonary fibrosis HP:0000218 High palate HP:0000554 Uveitis HP:0001287 Meningitis HP:0010651 Abnormal meningeal morphology

**List of Gene Candidates:**

(1) ADGRV1 (2) KIF3B (3) SEMA4C (4) ARL5C (5) BCAS3 (6) BCL11A (7) HIP1 (8) MACF1 (9) ORAI2 (10) BCL3 (11) NEK8 (12) ACSM1 (13) ABHD15 (14) ABCA7 (15) SPRR1B (16) VWCE (17) HRCT1 (18) BCAR1 (19) TRIP10 (20) ZNF674 (21) DNAAF1 (22) MAP4K2 (23) SLC47A2 (24) ADAM15 (25) ZNF776 (26) FDFT1 (27) HLA-A (28) GREB1 (29) PRSS22 (30) UNC13C (31) PKD1 (32) DDX11 (33) MRPS5 (34) APOB (35) RGS4 (36) RGMA (37) SCN10A (38) VIPR2 (39) SLC4A7 (40) PPP1R3A (41) MAP2K4 (42) GPANK1 (43) MYH10 (44) HIST1H1C (45) TNFSF12 (46) PGM2L1 (47) ANKLE2 (48) RP11-293B20.2 (49) MAS1 (50) AFDN (51) FAM83G (52) IL17RD (53) MAD1L1 (54) EP400 (55) MUC4 (56) PHLPP1 (57) UTP20 (58) SRBD1 (59) IGHV3-38 (60) TMPRSS12 (61) SLC24A2 (62) MGAT3 (63) NAA15 (64) CACNA1H (65) NECTIN2 (66) FBXO17 (67) GDF6 (68) SNAPC1 (69) PPP6R1 (70) ZNF713 (71) PLEKHB2 (72) NEDD4L (73) PKN1 (74) TMCC3 (75) SGSM1 (76) CASD1 (77) GLI2 (78) CEP131 (79) AHNAK (80) DRAXIN (81) CKMT2 (82) MIGA2 (83) CLUH (84) SLC22A10 (85) IRAK4 (86) CRIPAK (87) ALOX5AP (88) OBSCN (89) CEP72 (90) ZKSCAN5 (91) PTPRF (92) SAMD3 (93) GPRIN3 (94) STK10 (95) TALDO1 (96) PDE4DIP (97) RNF103 (98) PEX12 (99) DIP2B (100) ANKRD16 (101) PCED1A (102) OAZ1 (103) TARBP2 (104) BAGE2 (105) NOTCH2 (106) CASZ1 (107) NUDT4B (108) CHRNA10 (109) WDFY3 (110) PNMA5 (111) GABRD (112) CDC42EP4 (113) IGHG1 (114) FBXL4 (115) DYSF (116) LRRK1 (117) LRRC75B (118) CPNE7 (119) MUC2 (120) FADS6 (121) PANK4 (122) EEF1AKNMT (123) ZNF420 (124) XKR8 (125) AFAP1L1 (126) SORCS2 (127) MIRLET7BHG (128) FLCN (129) FBH1 (130) SETD5 (131) TFR2 (132) AEBP1 (133) BRAT1 (134) DTX1 (135) SULT1A2 (136) ATXN3 (137) SLC36A2 (138) HS3ST2 (139) SORL1 (140) TMPRSS11F (141) DLGAP5 (142) WDPCP (143) HSPG2 (144) DUOX2 (145) NBPF10 (146) LNP1 (147) LRIF1 (148) FAT4 (149) KLHL15 (150) MC1R (151) DSCAML1 (152) ACKR4 (153) TRIOBP (154) EXOC4 (155) ATRX (156) PALLD (157) FAM193B (158) MRNIP (159) AC018696.2 (160) LMNB1 (161) KLHL31 (162) NOX4 (163) ASS1 (164) CDH23 (165) TP53BP1 (166) CDK5RAP2 (167) SUSD1 (168) EPPK1 (169) MAP1A (170) FRG1JP (171) CIC (172) DENND1A (173) JMJD7-PLA2G4B (174) TEX35 (175) MCPH1 (176) PIAS4 (177) PAMR1 (178) VIT (179) SMARCD3 (180) FANK1 (181) AMIGO3 (182) MMRN2 (183) RASAL2 (184) ASCC2 (185) ELN (186) KRBA1 (187) PRKAG1 (188) FETUB (189) NKD1 (190) CRB1 (191) ANKRD13A (192) ZDHHC3 (193) NEPRO (194) ST8SIA4 (195) SREBF1 (196) SF3B3 (197) FZD1 (198) ABCB5 (199) KCNT1 (200) RUVBL2 (201) OR52N5 (202) HLA-J (203) NRK (204) HRNR (205) CRIM1 (206) SAMD1 (207) ZNF606 (208) TTC28 (209) DHX38 (210) PCDHB10 (211) GJD3 (212) ASXL3 (213) MRGPRE (214) DCP2 (215) QSOX1 (216) AIFM3 (217) KCNK6 (218) C16orf58 (219) PTCD1 (220) UBASH3A (221) RABEP2 (222) NUDT4 (223) MTSS1 (224) TCF3 (225) PIDD1 (226) TARS2 (227) OPTC (228) CDKL2 (229) ALS2CR12 (230) ARHGEF11 (231) PKHD1 (232) RANBP3L

# **Claude Sonnet 3.7 Response:**

I'll help you analyze this genetics case to determine which gene might have a causal mutation for the patient's condition. Let me examine the phenotype information and consider potential candidate genes.

Based on the case summary, we have a patient with:

- Central nervous system inflammatory disease
- Idiopathic pulmonary fibrosis
- Bilateral uveitis
- Pericarditis with myocarditis
- History of toxoplasmosis, seizures, and meningitis
- Brain MRI showing enhancing parenchymal lesions and multifocal leptomeningitis

The HPO terms include:

- Pericarditis (HP:0001701)
- Peripheral neuropathy (HP:0009830)
- Myocarditis (HP:0012819)
- Seizure (HP:0001250)
- Pulmonary fibrosis (HP:0002206)
- Uveitis (HP:0000554)
- Meningitis (HP:0001287)
- Additional terms related to joint hypermobility, keloids, etc.

Looking at the extensive gene list, I need to identify genes that could explain multiple aspects of this complex phenotype, particularly focusing on the inflammatory, cardiac, pulmonary, and neurological manifestations.

Several genes stand out as potential candidates:

1. **IRAK4** (Gene #85): This gene encodes interleukin-1 receptor-associated kinase 4, which plays a critical role in innate immunity. IRAK4 deficiency can cause recurrent bacterial infections and impaired inflammatory responses, which could explain the patient's history of infections and inflammatory conditions.
2. **NOTCH2** (Gene #105): The NOTCH signaling pathway is involved in multiple developmental processes and immune regulation. Mutations could explain the multi-system inflammatory manifestations.
3. **FLCN** (Gene #128): Folliculin is associated with Birt-Hogg-Dubé syndrome, which can include pulmonary manifestations, though typically cysts rather than fibrosis.
4. **NOX4** (Gene #162): This gene encodes NADPH oxidase 4, which is involved in the production of reactive oxygen species and has been implicated in fibrotic diseases, particularly pulmonary fibrosis.
5. **IL17RD** (Gene #52): This encodes an IL-17 receptor family member, which is involved in inflammatory responses. IL-17 signaling is particularly relevant to autoimmune conditions.

Among these, **IRAK4** appears particularly promising as it could explain the inflammatory aspects of this case, including the CNS inflammation, cardiac involvement, and history of infections. IRAK4 deficiency has been associated with immunodeficiency and susceptibility to pyogenic bacterial infections, which could relate to the patient's history.

# **Open AI o3-mini-high Response:**

Below are five detailed hypotheses—each with its own mechanistic rationale—for how a mutation in **IL17RD** (a candidate from the provided gene list) could be driving the patient’s complex multi‐organ inflammatory presentation:

### **Hypothesis 1: Dysregulation of IL‑17 Signaling Leading to Hyperinflammation**

**Rationale:** IL17RD (interleukin 17 receptor D) is known to act as a modulatory component in the IL‑17 signaling pathway. A loss‐of‐function or hypomorphic mutation could impair its normal inhibitory role, resulting in unchecked IL‑17 signaling.

- **Central Nervous System (CNS):** Enhanced IL‑17 activity can promote recruitment of inflammatory cells to the brain, potentially explaining the encephalitis, seizures, and leptomeningeal changes.
- **Pulmonary Fibrosis:** Excessive IL‑17 signaling may stimulate fibroblast proliferation and collagen deposition, contributing to the early-onset pulmonary fibrosis.
- **Ocular and Cardiac Inflammation:** The same mechanism could underpin the bilateral uveitis as well as myocarditis and pericarditis, since IL‑17 has been implicated in tissue-specific autoimmune inflammation.
- **Additional Phenotypes:** Peripheral neuropathy and abnormal meningeal morphology might also result from an inflammatory milieu driven by excessive IL‑17 activity.

### **Hypothesis 2: Aberrant T‑Cell Differentiation and Th17 Skewing**

**Rationale:** IL17RD is implicated in modulating immune cell signaling. A mutation may tilt the balance toward a proinflammatory Th17 phenotype.

- **Neuroinflammation:** Increased Th17 cells could drive chronic CNS inflammation, accounting for the aseptic meningitis, encephalitis, and subsequent seizure activity.
- **Cardiopulmonary Effects:** Th17 cytokines are known to mediate inflammatory responses in the heart and lungs, thus contributing to myocarditis, pericarditis, and pulmonary fibrosis.
- **Systemic Impact:** This dysregulation might also exacerbate peripheral neuropathy and inflammatory responses in other tissues, aligning with the broad spectrum of phenotypic abnormalities seen in the patient.

### **Hypothesis 3: Impaired Regulation of Tissue Repair and Fibrosis**

**Rationale:** IL‑17 signaling not only influences inflammation but also plays a role in tissue repair and fibrosis. A mutation in IL17RD could disrupt this balance.

- **Fibrotic Remodeling:** Loss of proper regulatory control might promote fibroblast activation and excessive extracellular matrix deposition, explaining the patient’s pulmonary fibrosis and even the formation of keloids.
- **Joint and Connective Tissue Effects:** Aberrant tissue remodeling might also underlie hyperextensibility of the finger joints, joint hypermobility, and even high-arched palate features by affecting connective tissue integrity.
- **Inflammatory Sequelae:** Chronic fibrosis in organs such as the lung could serve as a nidus for ongoing inflammatory processes, further driving disease progression in multiple systems.

### **Hypothesis 4: Disrupted Cytokine Network Leading to a Proinflammatory Cascade**

**Rationale:** IL17RD normally helps to maintain cytokine balance. A mutation could tip this balance toward a proinflammatory state by altering the interplay among cytokines.

- **Multi-organ Inflammation:** An imbalance in cytokine production can lead to systemic inflammatory responses that manifest as uveitis, myocarditis, pericarditis, and even CNS inflammatory lesions.
- **Secondary Effects:** This dysregulation may also contribute to peripheral neuropathy and abnormal meningeal morphology due to persistent low-grade inflammation damaging nerve fibers and meningeal tissues.
- **Biomarker Consistency:** The negative laboratory workup (including tests for various autoantibodies and inflammatory markers) might be explained by a subtle, cytokine-driven inflammatory process rather than a classic autoimmune antibody-mediated disease.

### **Hypothesis 5: Gene–Environment Interaction Triggered by Prior Infection**

**Rationale:** The patient’s history of toxoplasmosis and multiple inflammatory episodes suggests that an environmental trigger may have interacted with an underlying genetic susceptibility.

- **Infection as a Catalyst:** A mutation in IL17RD could impair the normal resolution of inflammation following infections. For example, after the toxoplasmosis episode at age 9, dysregulated IL‑17 signaling might have led to an exaggerated and prolonged inflammatory response.
- **Chronic Inflammation:** This maladaptive response might have set the stage for recurrent inflammatory episodes affecting the CNS (aseptic meningitis, encephalitis, seizures), the lungs (pulmonary fibrosis), and the heart (myocarditis, pericarditis).
- **Cumulative Damage:** Over time, this cycle of inadequate resolution and persistent inflammation could explain the cumulative damage observed across multiple organ systems, including subtle features such as joint hypermobility and keloid formation.

Each hypothesis connects the known or postulated functions of IL17RD to the specific phenotypes observed in the patient. By considering mechanisms such as hyperactive IL‑17 signaling, altered T-cell differentiation, aberrant tissue repair, cytokine imbalance, and gene–environment interactions, these hypotheses provide a comprehensive framework to explain how a mutation in IL17RD might be causal in this complex clinical case.

# **Open AI GPT 4.5 response:**

Based on the patient's phenotypic abnormalities and the candidate gene ADGRV1, here are five hypotheses detailing how mutations in ADGRV1 could be causally related to the patient's condition:​

**1. Seizures and Central Nervous System (CNS) Inflammation:**

Mutations in ADGRV1 have been associated with various forms of epilepsy, including febrile seizures and Rolandic epilepsy. Studies have identified ADGRV1 as a susceptibility gene for epilepsy, with variants linked to both febrile and afebrile seizures. Additionally, ADGRV1 is expressed in the central nervous system and plays roles in neuronal signaling pathways. Disruptions in these pathways due to ADGRV1 mutations could lead to neuronal hyperexcitability and inflammation, contributing to seizures and CNS inflammatory diseases.​[Wikipedia+4PubMed+4NCBI+4](https://pubmed.ncbi.nlm.nih.gov/36399868/?utm_source=chatgpt.com)[Wikipedia+1NCBI+1](https://en.wikipedia.org/wiki/GPR98?utm_source=chatgpt.com)

**2. Pulmonary Fibrosis:**

While ADGRV1 is primarily known for its roles in the nervous system and sensory organs, G protein-coupled receptors (GPCRs) like ADGRV1 are involved in various cellular processes, including those in the respiratory system. Although direct associations between ADGRV1 mutations and pulmonary fibrosis are not well-established, it's plausible that mutations could disrupt signaling pathways involved in lung tissue maintenance and repair, potentially contributing to pulmonary fibrosis.​[Gene Vision](https://gene.vision/knowledge-base/adgrv1-gene/?utm_source=chatgpt.com)

**3. Uveitis and Retinal Degeneration:**

ADGRV1 mutations are implicated in Usher syndrome type 2C, characterized by retinitis pigmentosa and sensorineural hearing loss. The protein localizes to the periciliary membrane complex in photoreceptor cells, essential for photoreceptor function. Disruptions can lead to retinal degeneration, which might manifest as uveitis. Therefore, ADGRV1 mutations could contribute to the patient's uveitis through retinal involvement.​[Frontiers+7Gene Vision+7Wiley Online Library+7](https://gene.vision/knowledge-base/adgrv1-gene/?utm_source=chatgpt.com)

**4. Myocarditis and Pericarditis:**

GPCRs, including ADGRV1, are expressed in various tissues, including the heart, and are involved in cardiovascular signaling pathways. Although specific links between ADGRV1 mutations and myocarditis or pericarditis are not well-documented, it's conceivable that such mutations could affect cardiac function or immune responses, leading to inflammatory conditions like myocarditis and pericarditis.​[Wiley Online Library+4OMIM+4Wikipedia+4](https://omim.org/entry/602851?utm_source=chatgpt.com)

**5. Peripheral Neuropathy:**

ADGRV1's expression in the central nervous system and its role in neuronal signaling suggest that mutations could impact peripheral nerve function. While direct evidence linking ADGRV1 mutations to peripheral neuropathy is limited, disruptions in neuronal signaling pathways due to such mutations could potentially contribute to peripheral neuropathic symptoms.​

In summary, mutations in ADGRV1 could plausibly contribute to the patient's phenotypic abnormalities through disruptions in various physiological pathways, particularly those involving the nervous system, sensory organs, and potentially other systems.

# **Deepseek R1 response:**

**Hypothesis 1: HLA-A (Gene 27) Mutation Leading to Autoimmune Dysregulation**

**Justification:** HLA-A is a critical component of the major histocompatibility complex (MHC) class I, involved in antigen presentation to T-cells. Specific HLA alleles are strongly associated with autoimmune diseases. For example, HLA-B27 is linked to uveitis and ankylosing spondylitis, while HLA-A29 is tied to birdshot chorioretinopathy. A mutation in HLA-A could disrupt self-tolerance, leading to cross-reactivity against antigens in the eyes (uveitis), heart (pericarditis/myocarditis), lungs (pulmonary fibrosis via autoimmune alveolitis), and CNS (aseptic meningitis/encephalitis). The patient’s multi-organ inflammation and negative autoimmune serologies suggest a novel HLA-mediated autoimmune mechanism rather than classical autoantibody-driven disease.

---

**Hypothesis 2: IL17RD (Gene 52) Dysregulation in IL-17 Signaling**

**Justification:** IL17RD modulates IL-17 receptor signaling, a pathway critical in mucosal immunity and autoinflammatory disorders. Overactivation of IL-17 is implicated in uveitis, pulmonary fibrosis, and meningeal inflammation. A loss-of-function mutation in IL17RD could lead to unchecked IL-17 signaling, driving neutrophilic infiltration in the eyes (uveitis), fibrotic remodeling in the lungs, and leptomeningeal inflammation (meningitis/encephalitis). The patient’s idiopathic pulmonary fibrosis and CNS inflammation align with IL-17’s role in recruiting neutrophils and fibroblasts, while reactive hypoglycemia may stem from systemic metabolic dysregulation due to chronic inflammation.

---

**Hypothesis 3: ALOX5AP (Gene 87) Mutation Causing Leukotriene-Driven Inflammation**

**Justification:** ALOX5AP encodes 5-lipoxygenase-activating protein, a key enzyme in leukotriene synthesis. Leukotrienes are potent pro-inflammatory mediators linked to asthma, cardiovascular inflammation, and fibrosis. A gain-of-function mutation in ALOX5AP could amplify leukotriene production, promoting pulmonary fibrosis (via fibroblast activation), pericarditis/myocarditis (vasculitis), and uveitis (leukotriene-mediated vascular leakage). Chronic leukotriene excess might also disrupt blood-brain barrier integrity, contributing to seizures and CNS lesions. The patient’s early-onset pulmonary fibrosis and recurrent serositis align with this pathway’s role in fibroinflammatory diseases.

---

**Hypothesis 4: IRAK4 (Gene 85) Gain-of-Function Mutation Hyperactivating Innate Immunity**

**Justification:** IRAK4 mediates Toll-like receptor (TLR) and IL-1 receptor signaling. Gain-of-function mutations can lead to excessive NF-κB activation, driving systemic autoinflammation. This could explain the patient’s multi-organ inflammation: pericarditis, myocarditis, uveitis, and CNS lesions (via microglial activation). TLR hyperactivation might also trigger pulmonary fibrosis through TGF-β crosstalk. Notably, the patient’s history of toxoplasmosis (a TLR2/4 agonist) could have exacerbated IRAK4-mediated inflammation. Unlike IRAK4 deficiency (associated with pyogenic infections), hyperactivation would manifest as sterile inflammation, consistent with negative infectious workups.

---

**Hypothesis 5: CIC (Gene 171) Loss-of-Function Causing Neuroinflammation and Systemic Autoimmunity**

**Justification:** CIC encodes a transcriptional repressor that regulates MAPK signaling. Biallelic CIC mutations are linked to neurodevelopmental disorders and brain lesions. A somatic or mosaic CIC mutation could impair repression of pro-inflammatory genes in the CNS (explaining seizures, encephalitis, and MRI lesions) and peripheral tissues. CIC dysfunction may also derepress immune regulators, leading to T-cell autoreactivity against lung, cardiac, and ocular tissues. The patient’s brain biopsy showing encephalitis and multi-system inflammation aligns with CIC’s dual role in neuronal homeostasis and immune modulation.

---

**Key Connections:**

- **HLA-A** and **IL17RD** directly tie to known autoimmune/autoinflammatory pathways affecting the observed organs.

- **ALOX5AP** and **IRAK4** highlight dysregulated innate immunity driving fibrosis and serositis.

- **CIC** bridges neuroinflammation and systemic autoimmunity, fitting the patient’s severe CNS involvement.

These hypotheses prioritize genes with plausible mechanistic links to the patient’s multi-organ phenotype and negative workup for conventional autoimmune/infectious etiologies.

# **Prompt 4 (Genetic Patient 2):**

Input prompt: Hypothesis generation prompt: You are a helpful genetic assistant. You need to

identify genes with causal mutations for these abnormalities for a patient. You are provided with

a summary of all the phenotypic abnormalities of the patient, along with a single gene that may

have a mutation that might cause the abnormalities. You are also given access to web search

results related to the gene. Your task is to generate 5 hypotheses for why the gene is causal to

the patient’s case. Make sure each hypothesis is thorough, providing detailed justifications for

why the gene is causal to each relevant phenotypic abnormality. Based on the current patient

information and the gene, find the top 5 possible hypotheses.

Case Summary: A young boy with history of global developmental delay since infancy. He was

delivered by C-section at 38 weeks due to decreased fetal movement and non-reassuring fetal

heart tones. Polyhydramnios was noted at 24 weeks gestation. He additionally has a history of

short stature, sagittal synostosis (dolichocephaly), unilateral choanal atresia, hypotonia, and 3

small VSDs. He has been evaluated for atypical movements that have been considered as

possibly athetosis. He also has a history of bilateral undescended testes and was noted to be

hypotonic in the newborn period. He has sparse hair and eyebrows since birth. He has 11

paired ribs. A brain MRI showed multiple small areas of T2 hyperintensity in the cortical white

matter. His exam was notable for sparse hair, down-slanted palpebral fissures, facial hypotonia,

overlapping toes, small 2nd and 5th nails on the hands, and low axial tone.

The HPO Terms are:

HP:0000268

HP:0000286

HP:0000453

HP:0000494

HP:0000639

HP:0000653

HP:0000678

HP:0000767

HP:0000878

HP:0000960

HP:0001263

HP:0001290

HP:0001601

HP:0001629

HP:0001741

HP:0001792

HP:0002015

HP:0002019

HP:0002020

HP:0002119

HP:0002170

HP:0002208

HP:0002307

HP:0002416

HP:0002500

HP:0003215

HP:0003396

HP:0004322

HP:0004442

HP:0007968

HP:0008070

HP:0008689

HP:0045075

HP:0200040

The gene list:

# **Claude Sonnet 3.7 response**:

I'll help you analyze genetic mutations that might be causing the patient's symptoms. Let me start by summarizing the patient's case and the key phenotypic abnormalities we need to explain.

## **Patient Summary**

This case involves a young boy with multiple congenital abnormalities including:

- Global developmental delay since infancy
- Decreased fetal movement and polyhydramnios during pregnancy
- Short stature
- Sagittal synostosis (dolichocephaly)
- Unilateral choanal atresia
- Hypotonia
- Three small VSDs (ventricular septal defects)
- Possible athetoid movements
- Undescended testes
- Sparse hair and eyebrows
- Skeletal abnormalities (11 paired ribs)
- Brain MRI abnormalities (T2 hyperintensity in cortical white matter)
- Facial features including down-slanted palpebral fissures and facial hypotonia
- Hand and foot abnormalities (overlapping toes, small nails)

Now, I need to select a gene from the provided list to analyze as a potential cause for these symptoms. Based on the phenotypic presentation, I'll analyze SHANK3, as it's associated with neurodevelopmental disorders that could explain several of these symptoms.

##

## **5 Hypotheses for SHANK3 Causality**

### **Hypothesis 1: SHANK3 Mutation Causing Phelan-McDermid Syndrome**

SHANK3 is strongly associated with Phelan-McDermid Syndrome (22q13.3 deletion syndrome), which includes many features present in this patient. SHANK3 encodes a scaffold protein in the postsynaptic density of excitatory synapses that is critical for proper synapse formation and neuronal signaling. A loss-of-function mutation could explain:

- Global developmental delay (HP:0001263): SHANK3 is crucial for cognitive development and synaptic function.
- Hypotonia (HP:0001290): Commonly seen in Phelan-McDermid syndrome due to impaired neuromuscular development.
- Abnormal brain MRI findings (HP:0002119): White matter abnormalities are consistent with SHANK3-related synaptopathy.
- Possible athetoid movements (HP:0002170): Abnormal movement patterns are reported in SHANK3 mutations due to basal ganglia dysfunction.

### **Hypothesis 2: SHANK3 Role in Craniofacial and Skeletal Development**

SHANK3 has been implicated in pathways affecting bone and cartilage development beyond its well-known neurological functions:

- Sagittal synostosis/dolichocephaly (HP:0001605): SHANK3 interacts with signaling pathways important for cranial suture development.
- Short stature (HP:0004322): Growth deficiency is common in Phelan-McDermid syndrome.
- Abnormal number of ribs (HP:0000878): Skeletal abnormalities can result from disrupted morphogenetic signaling during development.
- Abnormal toe morphology (HP:0001829): Skeletal abnormalities in the extremities are consistent with SHANK3's role in developmental patterning.

### **Hypothesis 3: SHANK3 Impact on Cardiac Development and Function**

SHANK3 mutations have been associated with cardiac defects in some cases:

- Ventricular septal defects (HP:0001629): SHANK3 expression has been documented in cardiac tissue during development.
- The presence of three small VSDs suggests a developmental issue in cardiac septation, potentially linked to SHANK3's role in cell adhesion and tissue organization.
- The combination of cardiac and neurological features is consistent with SHANK3's pleiotropic effects on multiple organ systems.

### **Hypothesis 4: SHANK3 Contribution to Respiratory and Midline Defects**

SHANK3 plays a role in neural crest cell migration and epithelial development:

- Choanal atresia (HP:0000453): This midline defect involves failed recanalization of the nasal passage, which depends on proper neural crest cell migration and epithelial-mesenchymal interactions.
- Polyhydramnios (HP:0001561): Often associated with swallowing difficulties due to neurological dysfunction, which could be explained by SHANK3's role in oropharyngeal development and neurological function.
- The combination of midline defects (choanal atresia) with neurological abnormalities is consistent with the pleiotropic effects of SHANK3 mutations.

### **Hypothesis 5: SHANK3 Effects on Ectodermal Development and Hormone Signaling**

SHANK3 participates in signaling pathways that affect ectodermal derivatives and endocrine function:

- Sparse hair and eyebrows (HP:0008070, HP:0000535): SHANK3 may affect ectodermal development through its interaction with growth factor signaling pathways.
- Cryptorchidism/undescended testes (HP:0000028): SHANK3 may influence hypothalamic-pituitary-gonadal axis development or function.
- Decreased fetal movement (HP:0001558): This could reflect both neurological dysfunction and muscular development issues related to SHANK3's role in synapse formation and neuromuscular junction development.
- Facial hypotonia (HP:0000332): Reflects SHANK3's role in neuromuscular development particularly affecting facial muscles.

Each of these hypotheses offers a mechanistic explanation for how a SHANK3 mutation could result in the constellation of symptoms observed in this patient. The multi-system involvement suggests that SHANK3 plays important roles in the development and function of neural, skeletal, cardiac, and other tissues, consistent with what is known about this gene's function and its association with Phelan-McDermid syndrome.

# **Open AI o3-mini-high response:**

Below are five detailed hypotheses for how a mutation in SMARCA4—a key component of the SWI/SNF chromatin remodeling complex—could underlie the patient’s multisystem phenotype:

**Hypothesis 1: Disrupted Neural Development Leading to Global Developmental Delay and White Matter Abnormalities** SMARCA4 plays an essential role in regulating gene expression during neurogenesis. A pathogenic variant could impair the proliferation, migration, and differentiation of neural progenitor cells. This disruption may result in global developmental delay, hypotonia (HP:0001263, HP:0001629), and abnormal brain myelination or white matter integrity (reflected by the multiple small T2 hyperintense areas seen on MRI). The atypical movements (possibly athetosis) could further stem from defective neural circuit formation.

**Hypothesis 2: Abnormal Craniofacial and Skeletal Patterning Resulting in Sagittal Synostosis and Short Stature** SMARCA4 is involved in the transcriptional regulation of genes critical for osteoblast differentiation and craniofacial development. A mutation could lead to premature suture closure (sagittal synostosis, HP:0000219) resulting in dolichocephaly, as well as contribute to short stature (HP:0004322). Additionally, its role in mesenchymal cell lineage specification might explain the presence of an extra rib (11 paired ribs) and other skeletal anomalies.

**Hypothesis 3: Impaired Cardiac Development Leading to Multiple Ventricular Septal Defects (VSDs)** The SWI/SNF complex, with SMARCA4 as a core subunit, regulates several genes implicated in cardiogenesis. A mutation could alter the expression patterns necessary for proper cardiac septation and myocardial differentiation, thereby predisposing to congenital heart defects such as the three small VSDs observed in the patient. This dysregulation is consistent with the cardiac phenotype (HP:0004322).

**Hypothesis 4: Defective Gonadal Development Underlying Bilateral Undescended Testes** SMARCA4 is also important for regulating genes in developmental pathways beyond neural and skeletal systems. Its dysfunction may disturb the hormonal or local tissue signaling required for testicular descent, leading to bilateral undescended testes. This disruption in gonadal development may be further linked to the generalized hypotonia observed at birth (HP:0000286) and supports the broader role of SMARCA4 in organogenesis.

**Hypothesis 5: Abnormal Ectodermal Differentiation Resulting in Sparse Hair, Eyebrow Hypotrichosis, and Nail Anomalies** The gene’s role in chromatin remodeling affects not only mesenchymal and neural cells but also ectodermal derivatives. A SMARCA4 mutation could interfere with the normal differentiation of hair follicles and nail beds, leading to the congenital presentation of sparse hair and eyebrows (HP:0007968, HP:0003396) and the noted hypoplasia of the 2nd and 5th nails. These ectodermal changes, coupled with digital anomalies (e.g., overlapping toes, HP:0004322), reinforce the pleiotropic effects of impaired chromatin remodeling in this syndrome.

Each hypothesis links the known functions of SMARCA4 with specific components of the patient’s phenotype, suggesting that a mutation in this gene could have widespread developmental consequences across multiple organ systems.

# **Open AI GPT 4.5 response**:

Based on the patient's clinical presentation and the involvement of the FMR1 gene, here are five hypotheses detailing how mutations in FMR1 could be causative:

**Hypothesis 1: FMR1 Mutation Leading to Fragile X Syndrome (FXS) with Atypical Presentation**

Fragile X Syndrome (FXS) is typically associated with intellectual disability, developmental delay, and distinctive physical features. While macroorchidism (enlarged testes) is a hallmark of FXS, there have been reports linking FMR1 mutations to undescended testes (cryptorchidism). For instance, a study documented individuals with FMR1 microduplications presenting with undescended testes requiring surgical intervention. Additionally, hypotonia and developmental delays observed in the patient align with common manifestations of FXS. Therefore, an atypical presentation of FXS due to an FMR1 mutation could explain these phenotypic abnormalities.​[ScienceDirect+2ResearchGate+2Medbullets Step 2+2](https://www.researchgate.net/publication/11583592_Testicular_Torsion_in_an_Adolescent_With_Fragile_X_Syndrome?utm_source=chatgpt.com)[PMC](https://pmc.ncbi.nlm.nih.gov/articles/PMC3476717/?utm_source=chatgpt.com)

**Hypothesis 2: FMR1 Premutation Associated with Fragile X-Associated Tremor/Ataxia Syndrome (FXTAS) Manifesting Early**

FXTAS is a neurodegenerative disorder linked to FMR1 premutations, typically presenting in older adults with symptoms like tremor, ataxia, and cognitive decline. However, there is emerging evidence suggesting that FMR1 premutations can lead to white matter abnormalities and movement disorders earlier in life. A study highlighted that carriers of FMR1 premutations might exhibit white matter lesions correlated with motor and cognitive impairments. The patient's brain MRI showing T2 hyperintensities in the cortical white matter, along with atypical movements resembling athetosis, could be indicative of an early-onset manifestation of FXTAS or a related FMR1-associated condition.​[Movement Disorders+6Wikipedia+6SpringerLink+6](https://en.wikipedia.org/wiki/Fragile_X-associated_tremor/ataxia_syndrome?utm_source=chatgpt.com)[PMC+1ResearchGate+1](https://pmc.ncbi.nlm.nih.gov/articles/PMC6700239/?utm_source=chatgpt.com)

**Hypothesis 3: FMR1 Mutation Contributing to Craniosynostosis and Dolichocephaly**

Craniosynostosis, particularly sagittal synostosis leading to dolichocephaly, involves the premature fusion of skull sutures resulting in an elongated head shape. While FMR1 mutations are not classically associated with craniosynostosis, genetic factors play a significant role in suture development and fusion. Given the patient's presentation with sagittal synostosis and the known impact of FMR1 on neural development, it's plausible that an FMR1 mutation could contribute to abnormal cranial suture fusion, leading to dolichocephaly. ​[St. Louis Children's Hospital+6Mayo Clinic+6OMIM+6](https://www.mayoclinic.org/diseases-conditions/craniosynostosis/symptoms-causes/syc-20354513?utm_source=chatgpt.com)

**Hypothesis 4: FMR1 Mutation Leading to Congenital Heart Defects**

Congenital heart defects, such as ventricular septal defects (VSDs), have been associated with various genetic syndromes. While FMR1 is primarily linked to neurological manifestations, its role in early embryonic development suggests that mutations could impact cardiac development. The presence of multiple small VSDs in the patient might be related to an underlying FMR1 mutation affecting the structural formation of the heart during development. ​

**Hypothesis 5: FMR1 Mutation Resulting in Sparse Hair and Eyebrows Due to Developmental Anomalies**

The FMR1 gene provides instructions for making the FMRP protein, which is involved in various developmental processes. Mutations in FMR1 could disrupt normal ectodermal development, leading to features such as sparse hair and eyebrows observed in the patient. Although not a classic feature of FMR1-related disorders, the involvement of FMR1 in developmental pathways could plausibly result in such ectodermal anomalies. ​[PMC+4MedlinePlus+4Medical News Today+4](https://medlineplus.gov/genetics/gene/fmr1/?utm_source=chatgpt.com)

These hypotheses underscore the diverse roles of the FMR1 gene in development and how its mutation can lead to a spectrum of phenotypic abnormalities observed in the patient.

# **Deepseek R1 response:**

Hypothesis 1: CHD4 Mutation (Sifrim-Hitz-Weiss Syndrome)

Justification: CHD4 mutations cause Sifrim-Hitz-Weiss syndrome (SHWIS), characterized by congenital heart defects (e.g., VSDs), choanal atresia, genital anomalies (undescended testes), and developmental delay. The patient’s VSDs, choanal atresia, and cryptorchidism align with SHWIS. CHD4 regulates chromatin remodeling; disruptions could impair neural crest cell migration, explaining craniofacial defects (dolichocephaly, down-slanted palpebral fissures). Hypotonia and white matter T2 hyperintensities may stem from neurodevelopmental dysregulation. SHWIS also includes sparse hair, matching the patient’s phenotype.

Hypothesis 2: KMT2B Mutation (Dystonia 28, Childhood-Onset)

Justification: KMT2B mutations cause early-onset dystonia with developmental delay, hypotonia, and movement disorders (e.g., athetosis). The patient’s atypical movements and hypotonia fit this profile. KMT2B regulates histone methylation, critical for basal ganglia development. Dysfunction may disrupt motor pathways, leading to athetosis. While craniosynostosis is atypical, secondary white matter changes on MRI (T2 hyperintensities) could reflect neuronal dysfunction. Sparse hair and facial hypotonia may arise from broader epigenetic dysregulation.

Hypothesis 3: FAT1 Mutation (Craniosynostosis Syndrome)

Justification: FAT1 mutations are linked to craniosynostosis, facial dysmorphism (dolichocephaly, down-slanted palpebral fissures), and developmental delay. FAT1 regulates planar cell polarity, critical for cranial suture patterning; loss-of-function could drive sagittal synostosis. Hypotonia and white matter anomalies may reflect aberrant neuronal migration. FAT1 also influences myogenesis, potentially contributing to skeletal muscle hypotonia. While rib anomalies are less typical, FAT1’s role in skeletal development could explain this feature.

Hypothesis 4: IFT140 Mutation (Ciliopathy-Related Skeletal Dysplasia)

Justification: IFT140, involved in intraflagellar transport, is linked to ciliopathies like Jeune syndrome. The patient’s 11 paired ribs and polyhydramnios (from reduced fetal movement) align with skeletal ciliopathies. Cilia dysfunction can disrupt Hedgehog signaling, impairing skeletal and cardiac development (VSDs). Choanal atresia and cryptorchidism are also ciliopathy features. Hypotonia and white matter changes may arise from neuronal cilia defects affecting brain development. Sparse hair could reflect ciliary roles in follicular morphogenesis.

Hypothesis 5: DHCR7 Mutation (Smith-Lemli-Opitz Syndrome, SLOS)

Justification: DHCR7 mutations cause SLOS, characterized by developmental delay, microcephaly (or dolichocephaly), genital anomalies, and 2-3 toe syndactyly (overlapping toes in this case). DHCR7 catalyzes cholesterol synthesis; deficiency disrupts sonic hedgehog signaling, explaining craniofacial, cardiac (VSDs), and neurodevelopmental defects. Hypotonia and white matter anomalies may reflect altered myelination. Sparse hair and eyebrows are common in SLOS due to cholesterol-dependent follicular development. Polyhydramnios could stem from fetal hypotonia.

Conclusion: These hypotheses prioritize genes (CHD4, KMT2B, FAT1, IFT140, DHCR7) that collectively explain craniosynostosis, VSDs, choanal atresia, hypotonia, and neurodevelopmental/structural anomalies. Functional pathways (chromatin remodeling, ciliogenesis, cholesterol synthesis) align with the patient’s multi-organ involvement. Further testing (e.g., exome sequencing, methylation studies) should prioritize these candidates.
