# Supplementary material for: Genetic Diagnosis and Discovery Enabled by Large Language Models
Source: Adv Sci (Weinh). 2026 Feb 8;13(22):e18656. doi: 10.1002/advs.202518656 (PMC13088295; doi:10.1002/advs.202518656)
Supplement: Supplementary file 11 — Supporting File 11: advs74268‐sup‐0011‐SupplementalDataFile10.docx. [file ADVS-13-e18656-s001.docx]

**Supplemental Data File 10**

**Members of the Undiagnosed Diseases Network**

Maria T. Acosta

David R. Adams

Ben Afzali

Ali Al-Beshri

Eric Allenspach

Aimee Allworth

Raquel L. Alvarez

Justin Alvey

Ashley Andrews

Euan A. Ashley

Carlos A. Bacino

Guney Bademci

Ashok Balasubramanyam

Dustin Baldridge

Erin Baldwin

Elsa Balton

Michael Bamshad

Deborah Barbouth

Pinar Bayrak-Toydemir

Anita Beck

Alan H. Beggs

Edward Behrens

Gill Bejerano

Hugo J. Bellen

Jimmy Bennett

Jonathan A. Bernstein

Gerard T. Berry

Stephanie Bivona

Elizabeth Blue

Devon Bonner

Nicholas Borja

Lorenzo Botto

Steven Boyden

Lauren C. Briere

Elizabeth A. Burke

Lindsay C. Burrage

Manish J. Butte

Russell Butterfield

Peter Byers

William E. Byrd

Kaitlin Callaway

John Carey

George Carvalho

Thomas Cassini

Sirisak Chanprasert

Hsiao-Tuan Chao

Ivan Chinn

Gary D. Clark

Terra R. Coakley

Laurel A. Cobban

Joy D. Cogan

Matthew Coggins

F. Sessions Cole

Brian Corner

Rosario I. Corona

William J. Craigen

Andrew B. Crouse

Vishnu Cuddapah

Precilla D’Souza

Hongzheng Dai

Nitsuh K. Dargie

Kahlen Darr

Surendra Dasari

Joie Davis

Margaret Delgado

Esteban C. Dell'Angelica

Katrina Dipple

Daniel Doherty

Naghmeh Dorrani

Jessica Douglas

Emilie D. Douine

Dawn Earl

Lisa T. Emrick

Christine M. Eng

Cecilia Esteves

Kimberly Ezell

Elizabeth L. Fieg

Paul G. Fisher

Brent L. Fogel

Jiayu Fu

William A. Gahl

Rebecca Ganetzky

Emily Glanton

Ian Glass

Page C. Goddard

Joanna M. Gonzalez

Andrea Gropman

Meghan C. Halley

Rizwan Hamid

Neal Hanchard

Kelly Hassey

Nichole Hayes

Frances High

Anne Hing

Fuki M. Hisama

Ingrid A. Holm

Jason Hom

Martha Horike-Pyne

Alden Huang

Yan Huang

Anna Hurst

Wendy Introne

Gail P. Jarvik

Suman Jayadev

Orpa Jean-Marie

Vaidehi Jobanputra

Oguz Kanca

Yigit Karasozen

Shamika Ketkar

Dana Kiley

Gonench Kilich

Eric Klee

Shilpa N. Kobren

Isaac S. Kohane

Jennefer N. Kohler

Bruce Korf

Susan Korrick

Deborah Krakow

Elijah Kravets

Seema R. Lalani

Christina Lam

Brendan C. Lanpher

Ian R. Lanza

Kumarie Latchman

Kimberly LeBlanc

Brendan H. Lee

Kathleen A. Leppig

Richard A. Lewis

Pengfei Liu

Nicola Longo

Joseph Loscalzo

Richard L. Maas

Ellen F. Macnamara

Calum A. MacRae

Valerie V. Maduro

AudreyStephannie Maghiro

Rachel Mahoney

May Christine V. Malicdan

Rong Mao

Ronit Marom

Gabor Marth

Beth A. Martin

Martin G. Martin

Julian A. Martínez-Agosto

Shruti Marwaha

Allyn McConkie-Rosell

Ashley McMinn

Matthew Might

Mohamad Mikati

Danny Miller

Ghayda Mirzaa

Breanna Mitchell

Paolo Moretti

Marie Morimoto

John J. Mulvihill

Lindsay Mulvihill

Mariko Nakano-Okuno

Stanley F. Nelson

Serena Neumann

Thomas J. Nicholas

Donna Novacic

Devin Oglesbee

James P. Orengo

Rebecca Overbury

Laura Pace

Stephen Pak

J. Carl Pallais

Neil H. Parker

LéShon Peart

Leoyklang Petcharet

John A. Phillips III

Filippo Pinto e Vairo

Jennifer E. Posey

Lorraine Potocki

Barbara N. Pusey Swerdzewski

Aaron Quinlan

Daniel J. Rader

Ramakrishnan Rajagopalan

Deepak A. Rao

Anna Raper

Wendy Raskind

Adriana Rebelo

Chloe M. Reuter

Lynette Rives

Lance H. Rodan

Martin Rodriguez

Jill A. Rosenfeld

Elizabeth Rosenthal

Francis Rossignol

Maura Ruzhnikov

Marla Sabaii

Jacinda B. Sampson

Timothy Schedl

Lisa Schimmenti

Kelly Schoch

Daryl A. Scott

Elaine Seto

Vandana Shashi

Emily Shelkowitz

Sam Sheppeard

Jimann Shin

Edwin K. Silverman

Giorgio Sirugo

Kathy Sisco

Tammi Skelton

Cara Skraban

Carson A. Smith

Kevin S. Smith

Lilianna Solnica-Krezel

Ben Solomon

Rebecca C. Spillmann

Andrew Stergachis

Joan M. Stoler

Kathleen Sullivan

Shamil R. Sunyaev

Shirley Sutton

David A. Sweetser

Virginia Sybert

Holly K. Tabor

Queenie Tan

Arjun Tarakad

Herman Taylor

Mustafa Tekin

Willa Thorson

Cynthia J. Tifft

Camilo Toro

Alyssa A. Tran

Rachel A. Ungar

Adeline Vanderver

Dave Viskochil

Tiphanie P. Vogel

Colleen E. Wahl

Melissa Walker

Nicole M. Walley

Jennifer Wambach

Michael F. Wangler

Patricia A. Ward

Daniel Wegner

Monika Weisz Hubshman

Corrine K. Welt

Mark Wener

Tara Wenger

Monte Westerfield

Matthew T. Wheeler

Jordan Whitlock

Lynne A. Wolfe

Heidi Wood

Kim Worley

Shinya Yamamoto

Zhe Zhang

Stephan Zuchner
